# Ten new species of *Selaginella* (Lycopodiopsida, Selaginellaceae) from northern South America, primarily Venezuela, with a new status and combination, and distributional updates

**DOI:** 10.3897/phytokeys.276.191365

**Published:** 2026-06-11

**Authors:** Iván A. Valdespino, Christian A. López, Jorge I. Ceballos

**Affiliations:** 1 Departamento de Botánica, Facultad de Ciencias Naturales, Exactas y Tecnología, Universidad de Panamá, Apartado Postal 0824–00073, Panamá, Panama Departamento de Botánica, Facultad de Ciencias Naturales, Exactas y Tecnología, Universidad de Panamá Panamá Panama; 2 Sistema Nacional de Investigación (SNI), SENACYT, Panamá, Panama Department of Integrative Biology, University of Texas at Austin Austin United States of America; 3 Smithsonian Tropical Research Institute, Apartado Postal 0843-03092, Panamá, Panama Facultad de Ciencias y Tecnología, Universidad Tecnológica de Panamá Panamá Panama; 4 Department of Integrative Biology, University of Texas at Austin, 2415 Speedway #C0930, Austin, Texas 78712, USA Smithsonian Tropical Research Institute Panamá Panama; 5 Facultad de Ciencias y Tecnología, Universidad Tecnológica de Panamá, Panamá, Panama Sistema Nacional de Investigación (SNI), SENACYT Panamá Panama

**Keywords:** Cataniapo river, Cerro de la Neblina, Guayana Highlands, Idioblasts, Mawarinuma river, Taxonomy, Cerro de la Neblina, Idioblastos, Río Cataniapo, Río Mawarinuma, Taxonomía, Tierras Altas de Guayana

## Abstract

As part of the Selaginellaceae treatment on the “Lycophytes and Ferns of Venezuela,” led by Alan R. Smith (UC), ten new *Selaginella* species from northern South America, primarily found in Venezuela, are described. These include *S. cataniapensis* Valdespino, **sp. nov**., *S. cultellifolia* Valdespino & C.López, **sp. nov**., (also present in Colombia), *S. guaramacalensis* Valdespino & C.López, **sp. nov**., *S. liesneri* Valdespino, **sp. nov**., *S. mawarinumensis* Valdespino & C.López, **sp. nov**., *S. monoloba* A.R.Sm. ex C.López, Valdespino & Mostacero, **sp. nov**. (also present in Colombia), *S. mostaceroi* Valdespino & C.López, **sp. nov**., *S. plagiochiloides* Valdespino & C.López, **sp. nov**., *S. tricula* A.R.Sm. ex Valdespino & C.López, **sp. nov**., and *S. turingiana* Valdespino, **sp. nov**. Additionally, *S. anemosyra* Valdespino, **nom. et stat. nov**., is described and recognized at the species level, based on *S. flabellata* var. latifrons A.Braun. The new species are diagnosed using comparative morphology and contrasted with morphologically similar and closely related species. For each taxon, we provide illustrations from digitized herbarium material and scanning electron micrographs, along with preliminary conservation assessments using the IUCN categories and criteria.

## Abstract (es)

Como parte del tratamiento de las Selaginellaceae para “Lycophytes and Ferns of Venezuela”, liderado por Alan R. Smith (UC), se describen diez nuevas especies de *Selaginella* del norte de América del Sur, principalmente de Venezuela. Estas incluyen *S. cataniapensis* Valdespino, **sp. nov**., *S. cultellifolia* Valdespino & C.López, **sp. nov**. (también presente en Colombia), *S. guaramacalensis* Valdespino & C.López, **sp. nov**., *S. liesneri* Valdespino, **sp. nov**., *S. mawarinumensis* Valdespino & C.López, **sp. nov**., *S. monoloba* A.R.Sm. ex C.López, Valdespino & Mostacero, **sp. nov**. (también presente en Colombia), *S. mostaceroi* Valdespino & C.López, **sp. nov**., *S. plagiochiloides* Valdespino & C.López, **sp. nov**., *S. tricula* A.R.Sm. ex Valdespino & C.López, **sp. nov**., y *S. turingiana* Valdespino, **sp. nov**. Además, se describe y se reconoce a nivel de especie *S. anemosyra* Valdespino, **nom. et stat. nov**., basada en *S. flabellata* var. latifrons A.Braun. Las nuevas especies se diagnostican mediante la morfología comparativa y se contrastan con especies morfológicamente similares y estrechamente relacionadas. Para cada taxón, proporcionamos ilustraciones de material de herbario digitalizado y micrografías electrónicas de barrido, junto con evaluaciones preliminares de conservación basadas en las categorías y criterios de la UICN.

## Introduction

Ferns, lycophytes, and extinct seedless relatives (commonly called Pteridophytes) are a diverse group of plants with about 12,000 species (Smith et al. 2006; Moran 2008; PPG I 2016; PteridoPortal 2026). Among seedless vascular plants, *Selaginella* (Lycopodiopsida – Selaginellaceae) is the largest lycophyte genus, with 600–800 species, and it has a worldwide distribution, especially in tropical ecosystems (Jermy 1990; Valdespino 1993, 2015a, 2016; Mickel et al. 2004; Valdespino et al. 2015; PPG I 2016; Zhou and Zhang 2015; Weststrand and Korall 2016). There are approximately 350 *Selaginella* species in the Americas (Valdespino et al. 2018; Valdespino 2020), of which approximately 300 are native to the Neotropics. Alston et al. (1981) provided the most recent treatment of *Selaginella* for South America, identifying 133 species and 6 intraspecific taxa, including 53 confirmed for Venezuela. Smith (1985), in his unpublished “Pteridophytes of Venezuela, An Annotated Checklist,” listed 1,059 species and 24 varieties of ferns and lycophytes for Venezuela, including 76 *Selaginella* taxa, some of which were regarded as tentative new species. Recent work recognizes 1,155 fern and lycophyte species nationwide in Venezuela (Hokche et al. 2008), of which 88 correspond to *Selaginella* species currently reported for the country (Mostacero 2008). Further *Selaginella* species were later described, suggesting that at least 100 species are present in the country (Valdespino 2015b, 2016, 2017a, 2017b, 2017c, 2020; Valdespino et al. 2018).

Over the past forty years, extensive research on South American *Selaginella* has greatly expanded our knowledge of the genus, particularly regarding its diversity, taxonomy, and nomenclature, in countries such as Brazil, Colombia, Ecuador, and Venezuela (e.g., Steyermark 1986; Smith 1990; Valdespino 1992, 2015a, 2017a, 2017b, 2017c, 2020; Valdespino and Zimmer 2016; Valdespino et al. 2018, 2022a, 2022b, 2024a; Valdespino and López 2019; Vega-Betancur et al. 2023), while Smith (1995) treated the *Selaginella* species from the Venezuelan Guayana Highlands. In Colombia, a neighboring country of Venezuela, Murillo Aldana and Murillo (2017) documented 91 *Selaginella* taxa; this number has since increased to at least 94 species (Valdespino 2020; Valdespino et al. 2022a, 2022b, 2024b; Vega-Betancur et al. 2023).

We are currently preparing the taxonomic treatment of Selaginellaceae for the “Lycophytes and Ferns of Venezuela,” under the leadership of Alan R. Smith. As part of our broader collaborative effort on the taxonomy of *Selaginella* in the Neotropics and based on revisions of herbarium specimens and comparative analyses of macro- and micromorphological characters, we determined that several collections represent previously undescribed species. Here we describe ten new species of *Selaginella*, mostly from Venezuela: *S. cataniapensis* Valdespino, *S. cultellifolia* Valdespino & C.López (also found in Colombia), *S. guaramacalensis* Valdespino & C.López, *S. liesneri* Valdespino, *S. mawarinumensis* Valdespino & C.López, *S. monoloba* A.R.Sm. ex C.López, Valdespino & Mostacero (also found in Colombia), *S. mostaceroi* Valdespino & C.López, *S. plagiochiloides* Valdespino & C.López, *S. tricula* A.R.Sm. ex Valdespino & C.López, and *S. turingiana* Valdespino. We also elevate *Selaginella flabellata* (L.) Spring var. latifrons A.Braun at the species level as *S. anemosyra* Valdespino, and provide a new description for this species.

## Material and methods

Herbarium specimens from A, B, BM, BR, COL, F, G, GH, K, MA, MEXU, MICH, MO, NCU, NY, P, PH, PMA, TEX, UC, US, VEN, WIS, WVA, and YU (codes follow Thiers 2026) were examined and analyzed as described in Valdespino et al. (2024b). Digitized herbarium images available through the PteridoPortal (2026) were also reviewed. Conservation assessments were conducted in accordance with the IUCN Standards and Petitions Committee (2022) guidelines, incorporating information on threats and estimating the extent of occurrence (EOO) and area of occupancy (AOO) using GeoCAT (Bachman et al. 2011), with AOO calculated on the basis of a 2 × 2 km grid.

## Taxonomic treatment

### Selaginella anemosyra

Taxon classificationPlantaeSelaginellalesSelaginellaceae

Valdespino, nom. et stat. nov.

FB2E9289-8A0C-51D9-8BB7-686D72BBD103

urn:lsid:ipni.org:names:77381267-1

Figs 1, 2, 3, 4

≡ Selaginella flabellata (L.) Spring var. latifrons A.Braun, Ann. Sci. Nat. Bot. Ser. 5, 3: 278. 1865. Type. VENEZUELA. [Aragua]: Colonia Tovar, [10°25'00"N, 67°17'00"W, alt. 1800 m, 1856–7], *A. Fendler 493* (lectotype, designated by Alston et al. (1981: 256): BM-digital image! [BM000936516]; isolectotypes: B! [B 20 0130337], BR! [BR00000696516], G-digital images! [G00349357, G00349358], GH!, K-digital image! [K000677537], NY! [NY00029565], MO! [MO-1807677], PH-digital image! [PH00624914], YU! [YU.000624]).

#### Description.

***Plants*** terrestrial. ***Stems*** erect, stramineous, 21–84 cm in height, 2–4 mm diam., non-articulate, not flagelliform, stoloniferous, 1–3-branched. ***Rhizophores*** ventral and lateral, borne on the lower-most part of the stems and throughout stolons, (0.3) 0.5–1.0 mm diam. ***Leaves*** on main stems seemingly monomorphic and strongly appressed along the main stems up to 1.0–3.0 cm below the first or fourth branches, thereafter becoming heteromorphic throughout, coriaceous, those below the first or fourth branches deltate, ovate-deltate or broadly ovate, 2.0–4.0 × 2.0–3.0 mm; apices acute to apiculate, each 0.1–0.3 mm long; bases centrally truncate and raised with an inner and outer lobes or truncate to truncate-oblique, the outer bases often tufted with 2–5 short cilia or these absent due to abrasion, the inner bases glabrous, without distinct auricles; margins light green, the inner margins shortly ciliate along proximal 1/8, otherwise denticulate distally, the outer margins entire, except for the distal most portion denticulate, or the inner and outer margins entire along proximal 2/3 and denticulate on distal 1/3; upper surfaces deep green, corrugate to corrugate-striate, the lower surfaces shiny and light green to silvery green, slightly striate; apices acute to short-attenuate, 0.1–0.3 mm long, denticulate marginally and tipped by 1–3 teeth. ***Lateral leaves*** along the main stems distant, ascending at 45° to the stems, thereafter spreading and almost perpendicular to stems distally, oblong, 7.0–10 × 2.4–3.5 mm; bases truncate at center, the acroscopic portion lobed and strongly overlapping the stems, short- to long-ciliate, the basiscopic portion geniculate, free from the stems, entire; acroscopic margins on the upper surfaces narrowly hyaline, continuously bordered by a band 1 or 2 cells wide, the cells elongate, laevigate, and straight-walled, on the lower surfaces narrowly hyaline, continuously bordered by a band 2–4 cells wide, the cells elongate, laevigate, and straight-walled, short- to long-ciliate along proximal 1/3, otherwise entire, except denticulate on the distal 1/4, the basiscopic margins greenish, bordered by a band of 2 elongate cells, entire along proximal 2/3, otherwise denticulate along distal 1/3; apices falcate, acute to broadly acute, denticulate at tips with 3–6 teeth; upper surfaces comprising irregularly shaped, somewhat roundish, rectangular to quadrangular, sinuate-walled cells (often difficult to ascertain due to waxy cuticle), with many, evenly distributed, obscure, short quadrangular to round, or elongate, papillate idioblasts, each idioblast with the cell lumen with 8–16 papillae, without stomata, the lower surfaces comprising elongate, sinuate-walled, and laevigate cells, and elongate, straight-walled, and papillate idioblasts throughout, the papillae 10–20 in one row on each cell lumen, stomata on 3–10 rows along the midribs on distal 3/4 of the leaf laminae. ***Median leaves*** on the main stems after first branches imbricate and ascending, those along proximal third of the branches broadly ovate, thereafter and distally broadly ovate-orbicular to orbicular, midribs strongly arcuate, with the inner halves 1/2 narrower than the outer halves, 3.0–4.5 × 2.5–3.5 mm; bases obliquely truncate, the inner bases glabrous, the outer bases tufted with 4–6 short cilia, without auricles; margins narrowly hyaline, entire, except on distal most portion on the leaves below the third branches where they are denticulate, thereafter entire throughout; apices acute to short-attenuate, 0.2–0.5 mm long, marginally denticulate, tipped by 1–4 teeth; upper surfaces comprising irregularly quadrangular or elongate, sinuate-walled cells surrounded by interconnecting on a reticulate pattern or solitary, elongate, straight-walled, and papillate cells, the papillae 4–15 on each cell lumen, irregularly arranged, without elongate idioblasts, stomata in 1–7 rows along the midribs on central 1/4–3/4 of the leaf laminae, the lower surfaces comprising elongate, sinuate-walled cells, without elongate idioblasts, with few submarginal stomata on the outer halves of the leaf laminae. ***Axillary leaves*** on the main stems after first branches lanceolate, 2.4–5.0 × 0.7–2.2 mm, with the inner leaf halves slightly narrower than the outer leaf halves; inner bases rounded, the outer bases subcordate, each with a distinct, truncate center, with an inner, smaller, and an outer, larger, rounded lobe, the lobes short to long-ciliate; inner margins short- to long-ciliate along proximal 1/3, otherwise entire, except denticulate on distal 1/3, the outer margins long-ciliate along proximal 1/3–1/2, otherwise entire or denticulate along distal 1/4; apices gradually tapering, acute, tipped by 2–4 small teeth; both surfaces as in the lateral leaves. ***Strobili*** terminal on branch tips, solitary or bifurcate, quadrangular, 0.5–3.0 cm long. ***Sporophylls*** monomorphic, without a laminar flap, each with a faintly developed and seemingly glabrous keel along the midribs, ovate-lanceolate, 1.4–1.7 × 0.5–1.0 mm (the dorsal sporophylls usually 0.2 to 0.3 mm shorter than the ventral sporophylls); bases rounded; margins narrowly hyaline, bordered along distal 3/4 by a band 1 or 2 cells wide, these comprised of elongate, sinuate-walled, and papillate idioblasts, denticulate to serrulate; apices long-attenuate, 0.3–0.5 mm long, tipped by 2–3 teeth; stomata along the midribs on the upper surfaces; ***dorsal sporophylls*** greenish with the upper surfaces comprised of elongate, straight- to sinuate-walled, and laevigate cells, intermixed with elongate, straight-walled, and papillate idioblasts, except for the half that imbricates with the ventral sporophylls that is greenish- to silvery hyaline and composed of few elongate, sinuate-walled, and laevigate cells, intermixed with many elongate, straight-walled, and papillate idioblasts, the lower surfaces comprised of elongate, sinuate-walled, laevigate cells; ***ventral sporophylls*** with the upper and lower surfaces, silvery green, comprising cells as in the upper and lower surfaces of the dorsal sporophylls. ***Megasporangia*** in two ventral rows; ***megaspores*** white, the proximal faces rugulate-reticulate, reticulae ill-defined, open, and marginally delimited by low ridges, without an equatorial flange, the microstructure perforate, foveolate, and echinate, the distal faces reticulate, reticulae closed, delimited by low ridges, with the microstructure perforate, foveolate, and echinate, 275–325 µm diam. ***Microsporangia*** in two dorsal rows; ***microspores*** light orange, the proximal faces rugulate with echinate microstructure, the distal faces broadly baculate or occasionally broadly capitate, each baculum or caput and the rest of the surface with echinate microstructure, 19–27 µm diam.

**Figure 1.**
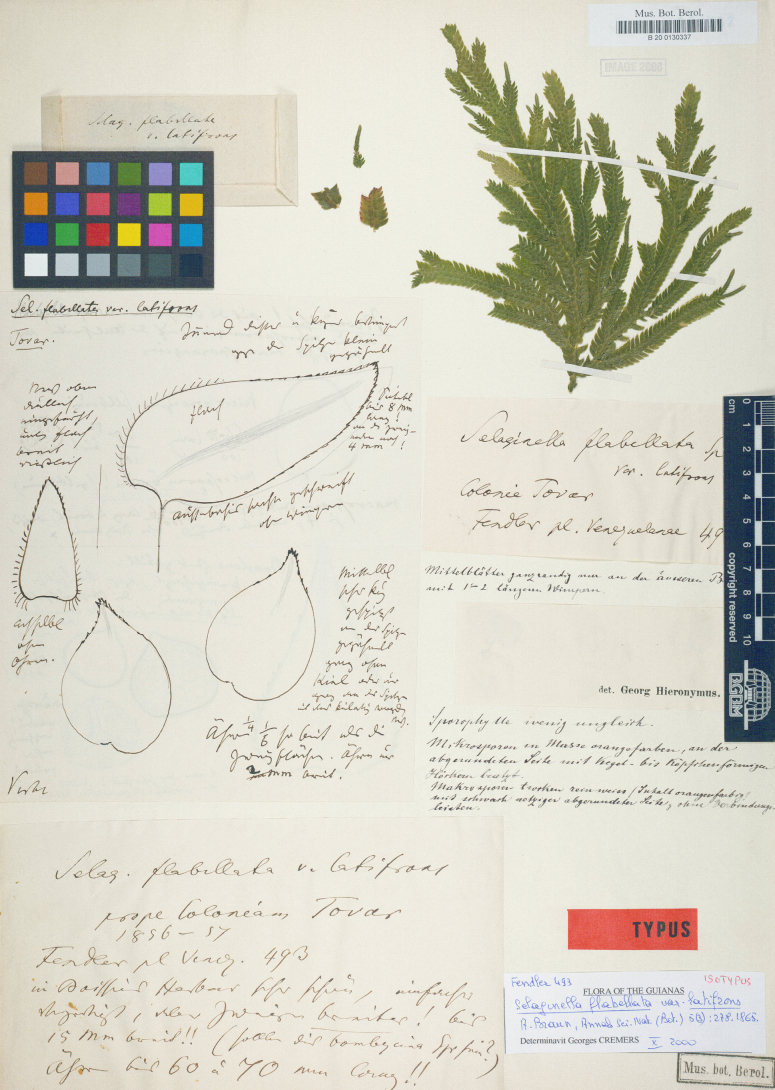
Isotype of *Selaginella anemosyra* Valdespino. Digitized image courtesy of the herbarium of the Berlin Botanical Garden and Botanical Museum, *A. Fendler 493*, B.

**Figure 2.**
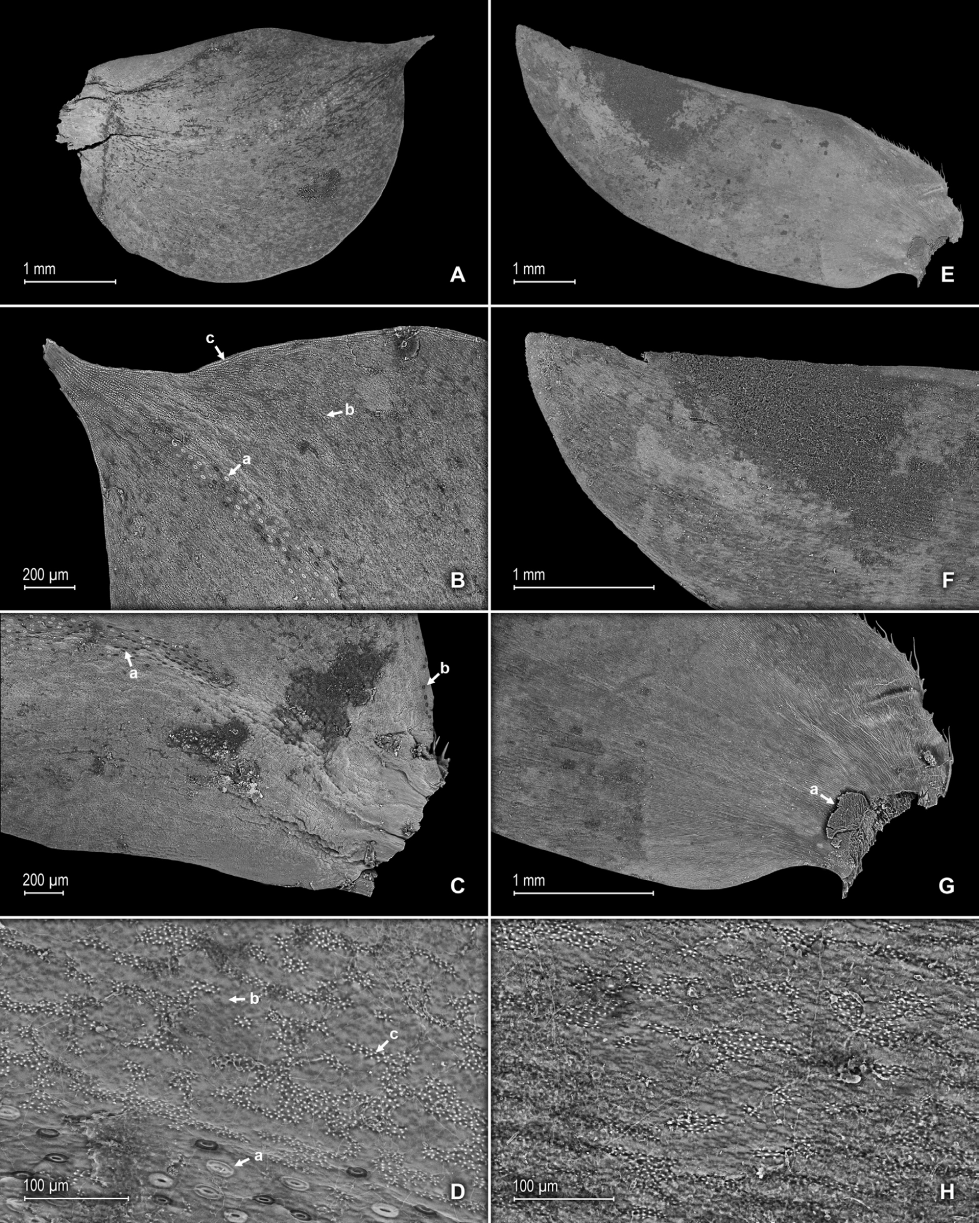
*Selaginella anemosyra* Valdespino. **A**. Median leaf, upper surface; **B**. Close-up of the distal portion and apex of the median leaf, upper surface; note stomata along the midrib (a), elongate, straight-walled, and papillate idioblasts on the leaf lamina (b) and margins (c); **C**. Close-up of the proximal portion and base of the median leaf, upper surface; note stomata along the midrib (a) and submarginally along the proximal portion of the outer margin (b); **D**. Close-up of the mid-section of the median leaf, upper surface; note stomata along the midrib (a), quadrangular or elongate, sinuate-walled, and laevigate cells (b), and elongate, straight-walled, and papillate cells (c); **E**. Lateral leaf, upper surface; **F**. Close-up of the distal portion and apex of the lateral leaf, upper surface; **G**. Close-up of the proximal portion and base of the lateral leaf, upper surface; note the ligule (a); **H**. Close-up of the mid-section of the lateral leaf, upper surface. **A–H** from *Ll. Williams & A.H.G. Alston 172*, US.

**Figure 3.**
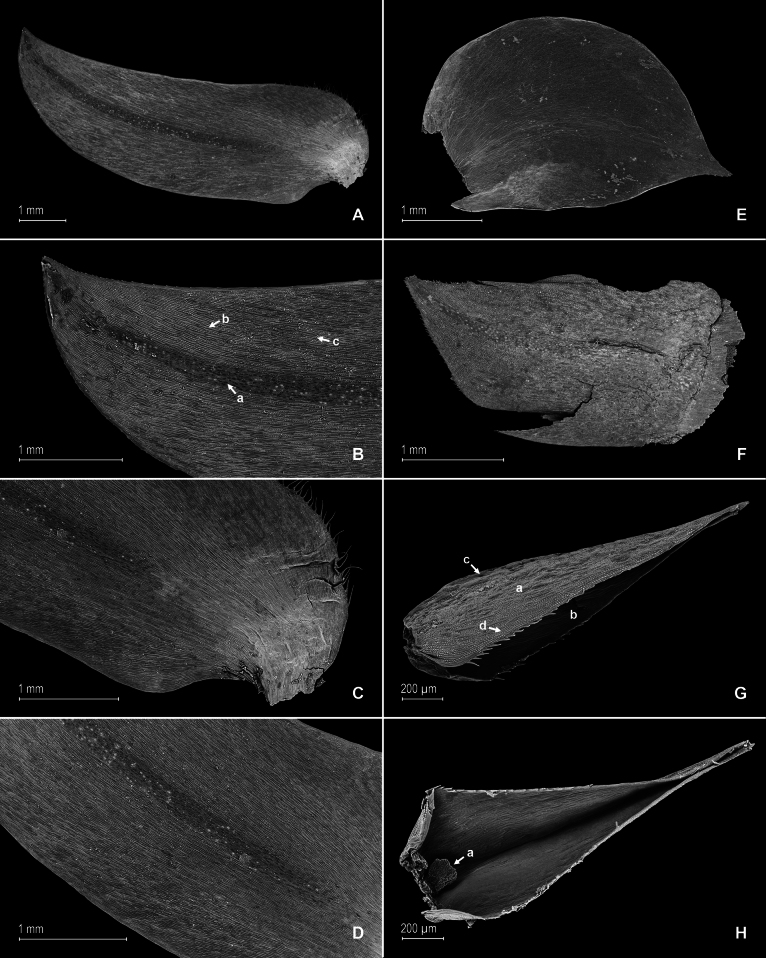
*Selaginella anemosyra* Valdespino. **A**. Lateral leaf, lower surface; **B**. Close-up of the distal portion and apex of the lateral leaf, lower surface; note stomata along the midrib (a), elongate, sinuate-walled, and laevigate cells (b), and elongate, straight-walled, and papillate idioblasts (c); **C**. Close-up of the proximal portion and base of the lateral leaf, lower surface; note stomata and features of the leaf lamina as in **B**; **D**. Close-up of the mid-section of the lateral leaf, lower surface; note stomata and features of the leaf lamina as in **B**; **E**. Median leaf, lower surface; **F**. Leaf along the main stem before branching, upper surface; **G**. Dorsal sporophyll, upper surface; showing half of the lamina that imbricates with the ventral sporophyll (a), lower surface of the sporophylls (b), stomata along the midrib (c), and elongate, straight-walled, and papillate idioblasts (d); **H**. Dorsal sporophylls, lower surface; note the ligule (a). **A–H** from *Ll. Williams & A.H.G. Alston 172*, US.

**Figure 4.**
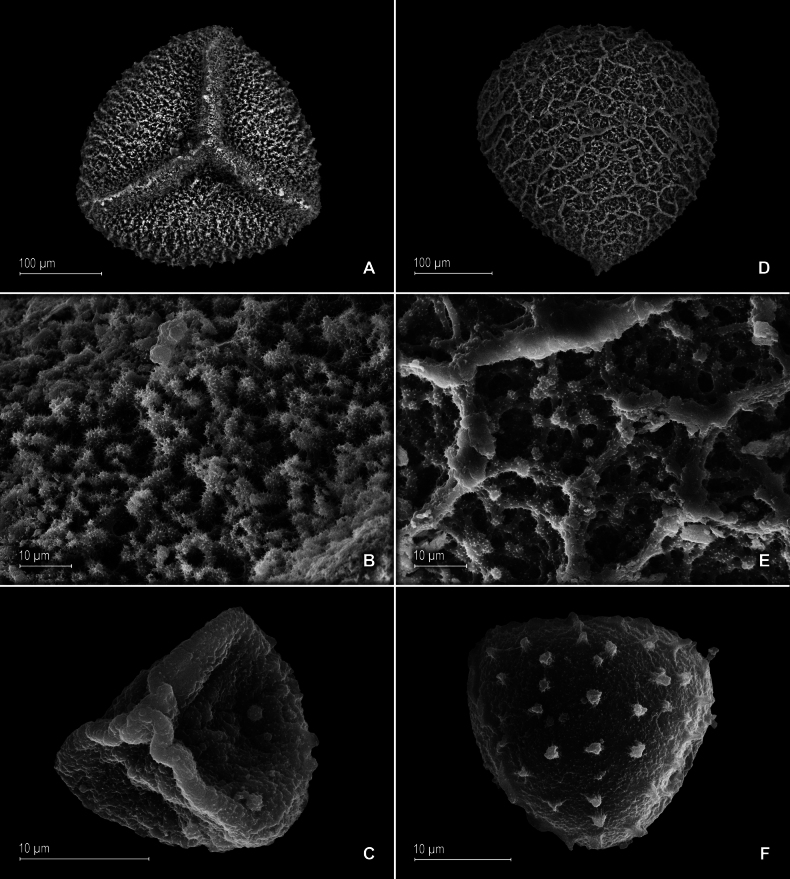
*Selaginella anemosyra* Valdespino. **A**. Megaspore, proximal face; **B**. Close-up of the megaspore, proximal face (same spore as in **A**); **C**. Microspore, proximal face; **D**. Megaspore, distal face; **E**. Close-up of the megaspore, distal face (same spore as in **D**); **F**. Microspore, distal face. **A–F** from *R.W. Long & K.J. Norstog 3547*, MO.

#### Habitat and distribution.

On steep slopes in dense, montane cloud forests at ca. 1110 m, presumably endemic to the State of Aragua, Venezuela.

#### Etymology.

The name is coined from the Gr. “*anemosyri*s,” a kind of fan, referring to the fan-shaped architecture of the stem’s leafy branches.

#### Conservation assessment.

*Selaginella anemosyra* is currently known from five collections, including the type, from the Caribbean coastal region of Venezuela, and appears to be restricted to the state of Aragua. However, the identity of this species has historically been confused with *S. flabellata*, so additional records may come to light as herbarium revision and targeted collecting continue. Nonetheless, based on the currently available material and ongoing habitat degradation in the region, we assess the species as Endangered (EN) B1ab(iii)+2ab(iii). Based on GeoCAT the species has an Extent of Occurrence (EOO) of 1,127.658 km^2^ and an Area of Occupancy (AOO) of 20 km^2^, both of which fall within Endangered thresholds under criterion B. Furthermore, there is evidence of ongoing decline in habitat extent/quality in the region, including historically high deforestation in southern Aragua (e.g., large forest losses reported for 1972–1990) (Torres-Lezama et al. 2010).

#### Additional specimen examined.

**Venezuela** • **Aragua**: Parque Nacional Aragua, near El Portachuelo, [ca. 10°12'23"N, 67°14'16"W], alt. 1100 m, 2 Dec. 1938, *Ll. Williams & Alston 172* (US), • 12 Dec. 1938, *Ll. Williams 10775* (F), Dec. 1939, *Ll. Williams 13603* (F); • Rancho Grande National Park [Henri Pittier National Park], ca. 20–30 km NW of Maracay, [10°22'48"N, 67°37'08"W, alt. 0–2436 m], 7 Oct. 1971, *R.W*. *Long & K.J. Norstog 3547* (MO, USF-digital image).

#### Discussion.

*Selaginella anemosyra* is characterized by its fern-like habit with erect stems and flabellate branches in outline, median leaves on the main stems with obliquely truncate bases, and its outer bases tufted with short hairs, as in *S. flabellata*. *Selaginella anemosyra* differs from *S. flabellata* by its flabellate (vs. pinnate) branches in outline, the main stems above the first branches (including lateral leaves) measuring 10–19 (vs. 4–8) mm across, lateral leaves oblong (vs. ovate to ovate-deltate), median leaves along proximal third branches broadly ovate, thereafter and distally broadly ovate-orbicular to orbicular (vs. broadly elliptic) with the outer halves twice as wide as the inner halves (vs. outer and inner leaf halves almost equal in width).

*Selaginella anemosyra* is based on *S. flabellata* var. latifrons, a taxon described from Venezuela, South America. According to Crabbe and Jermy (1973: 139), Alston had already regarded this entity as a distinct species by 1938. Apparently, he planned to name it after Conrad V. Morton from the Smithsonian National Herbarium (US) with the herbarium name “*S. mortoniana*,” since the epithet “latifrons” was unavailable because it was preoccupied by *S. latifrons* Hort. ex Williams [= *S. breynii* Spring] from South America and *S. latifrons* Warb. from the Philippines (Reed 1965–1966; 141). Crabbe and Jermy (1973: 139) did not classify *S. flabellata* var. latifrons as a separate species but instead regarded it as a variety of *S. flabellata* from the Lesser Antilles and named a different taxon with the name proposed by Alston. However, we believe that Alston was correct in considering the Venezuelan taxon sufficiently distinct from *S. flabellata*. Therefore, the variety is elevated to species rank as *S. anemosyra*.

### Selaginella cataniapensis

Taxon classificationPlantaeSelaginellalesSelaginellaceae

Valdespino sp. nov.

9A64B7E9-0FC4-5A07-9AB5-2B0ED6892626

urn:lsid:ipni.org:names:77381274-1

**Figure 5.**
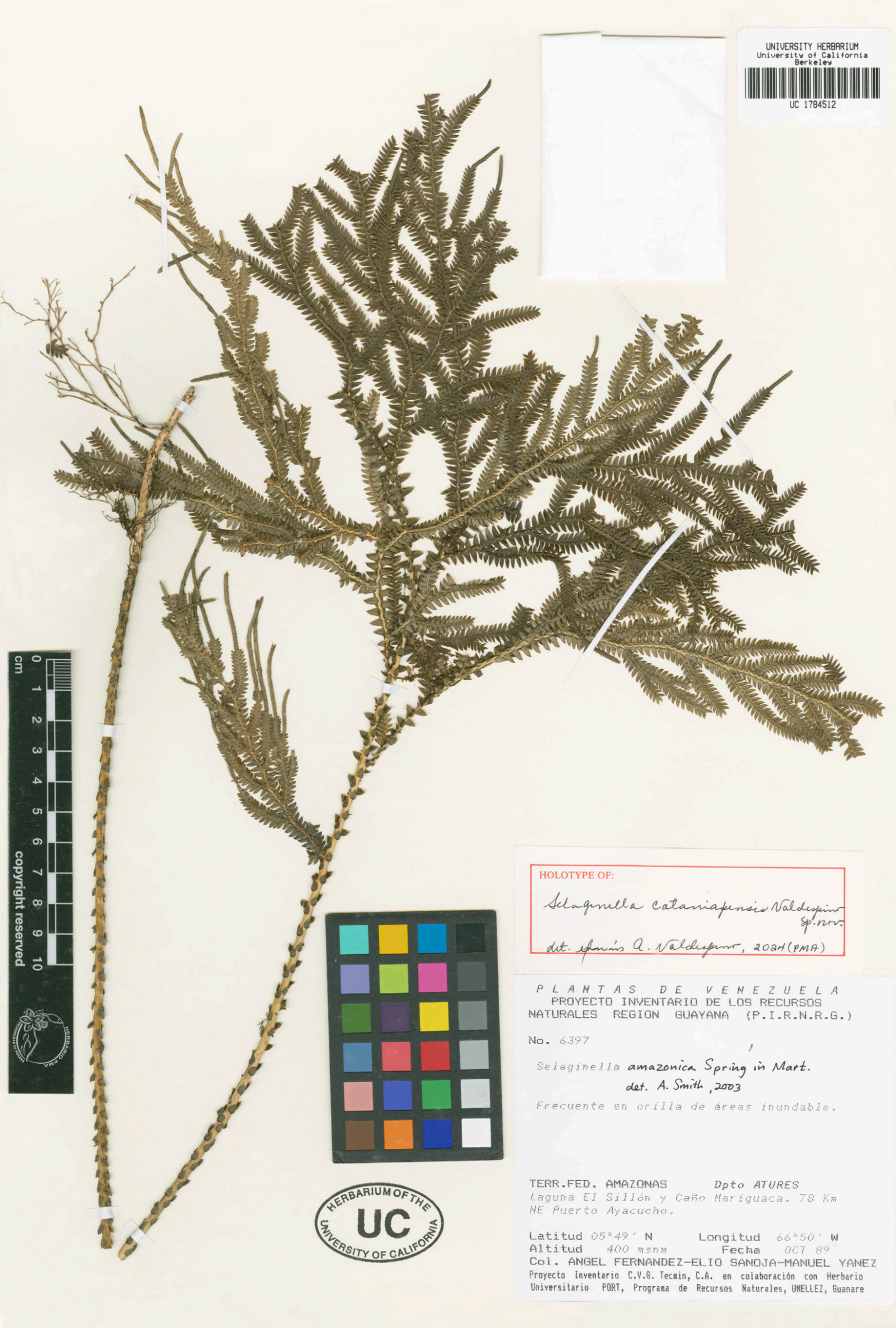
Holotype of *Selaginella cataniapensis* Valdespino. Digitized image courtesy of the herbarium of the University of Panama (PMA), from a loaned specimen from the herbarium of the University of California, *A. Fernández et al. 6397*, UC.

**Figure 6.**
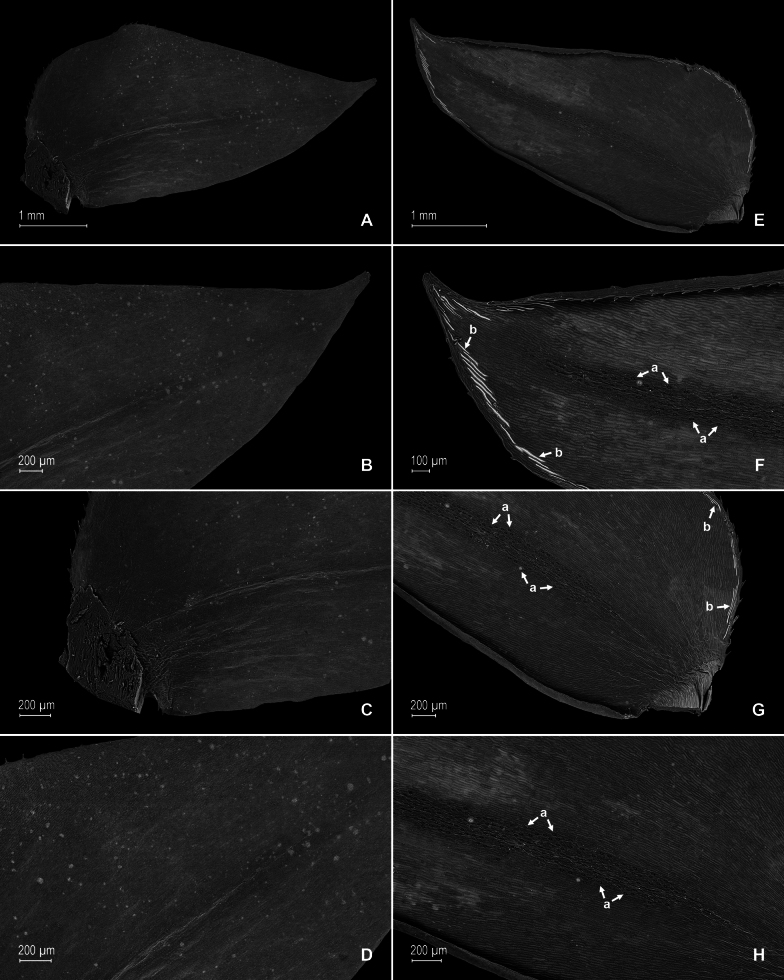
*Selaginella cataniapensis* Valdespino. **A**. Lateral leaf, upper surface; **B**. Close-up of the distal portion and apex of the lateral leaf, upper surface (same leaf as in **A**); **C**. Close-up of the proximal portion and base of the lateral leaf, upper surface (same leaf as in **A**); **D**. Close-up of the mid-section of the median leaf, upper surface (same leaf as in **A**); **E**. Lateral leaf, lower surface; **F**. Close-up of the distal portion and apex of the lateral leaf, lower surface (same leaf as in **E**); note stomata (a) and submarginal, straight-walled idioblasts (b); **G**. Close-up of the proximal portion and base of lateral leaf, lower surface (same leaf as in **E**); note stomata (a) and submarginal, straight-walled idioblasts (b); **H**. Close-up of mid-section of the lateral leaf, lower surface (same leaf as in **E**); note stomata (a). **A–H** from the holotype, *A. Fernández et al. 6397*, UC.

**Figure 7.**
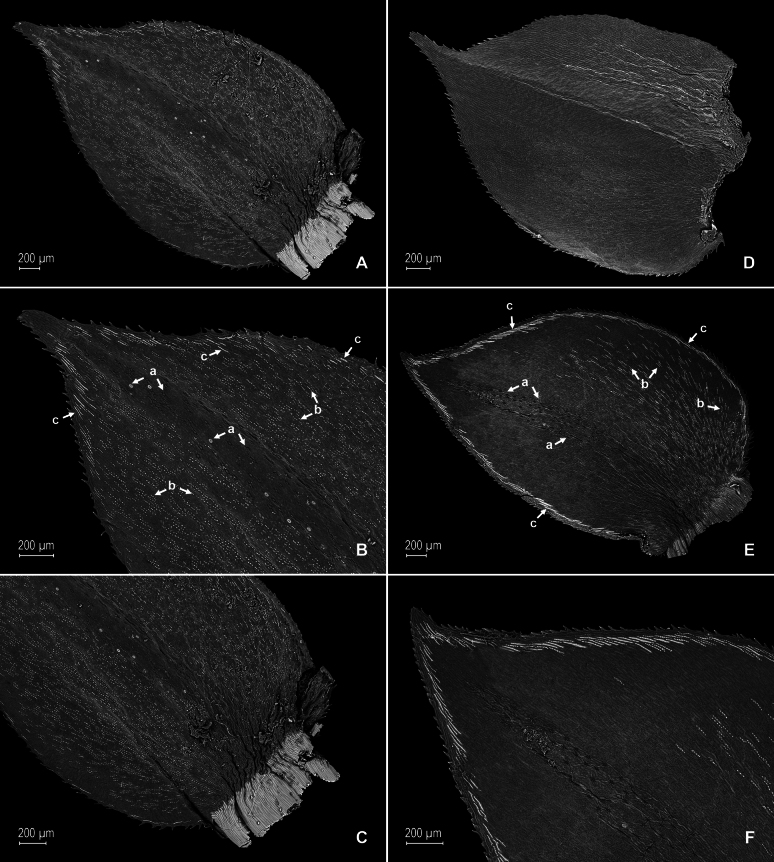
*Selaginella cataniapensis* Valdespino. **A**. Median leaf, upper surface; **B**. Close-up of the distal portion and apex of the median leaf, upper surface (same leaf as in **A**); note stomata (a) along the midrib and elongate, straight-walled, and papillate idioblasts on the leaf lamina (b) and submarginally (c); **C**. Close-up of the proximal portion and base of the median leaf, upper surface (same leaf as in **A**); note stomata along the midrib and idioblasts as in **B**; **D**. Median leaf, lower surface; **E**. Lateral leaf from the main stem below the first branches, lower surface; note stomata along the midrib (a) and elongate, straight-walled, and papillate idioblasts on acroscopic half of the lamina (b), and similar idioblasts submarginally (c); **F**. Close-up of the lateral leaf, distal portion, lower surface (same leaf as in **E**); note stomata and idioblasts on leaf lamina and margins as in **E**. **A–F** from the holotype, *A. Fernández et al. 6397*, UC.

**Figure 8.**
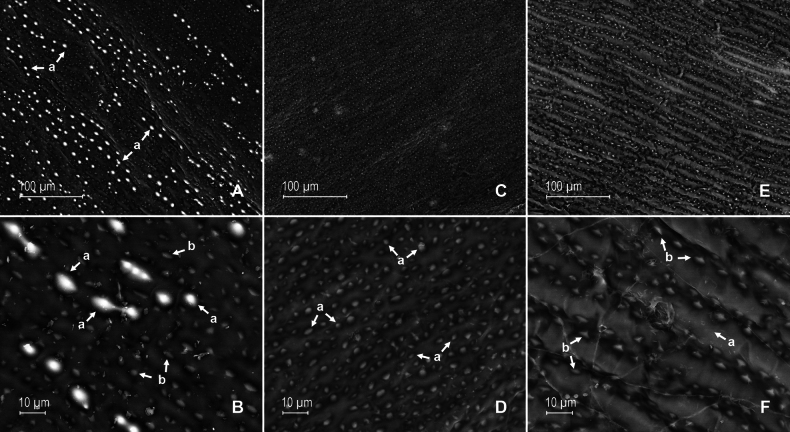
*Selaginella cataniapensis* Valdespino. **A**. Close-up of the median leaf, upper surface; note elongate to quadrangular, straight-walled, and papillate idioblasts (a); **B**. Close-up of the median leaf, upper surface (same leaf as in **A**); note elongate to quadrangular, straight-walled, and papillate idioblasts (a) and the cell margins bordered by pit-like structures (b); **C**. Close-up of the lateral leaf, upper surface; **D**. Close-up of the lateral leaf, upper surface (same leaf as in **C**); note cell margins bordered by pit-like structures (a); **E**. Close-up of the lateral leaf, lower surface; **F**. Close-up of the lateral leaf, lower surface (same leaf as in **E**); note elongate, sinuate-walled, and laevigate cells (a) and the cell margins bordered by pit-like structures (b). **A–F** from the holotype, *A. Fernández et al. 6397*, UC.

**Figure 9.**
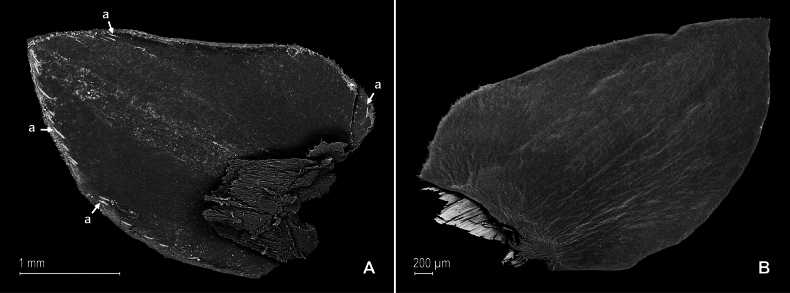
*Selaginella cataniapensis* Valdespino. **A**. Axillary leaf, lower surface; note submarginal, elongate, straight-walled, and papillate idioblasts (a); **B**. Axillary leaf, upper surface. **A, B** from a paratype, *A. Castillo 2046*, MO.

**Figure 10.**
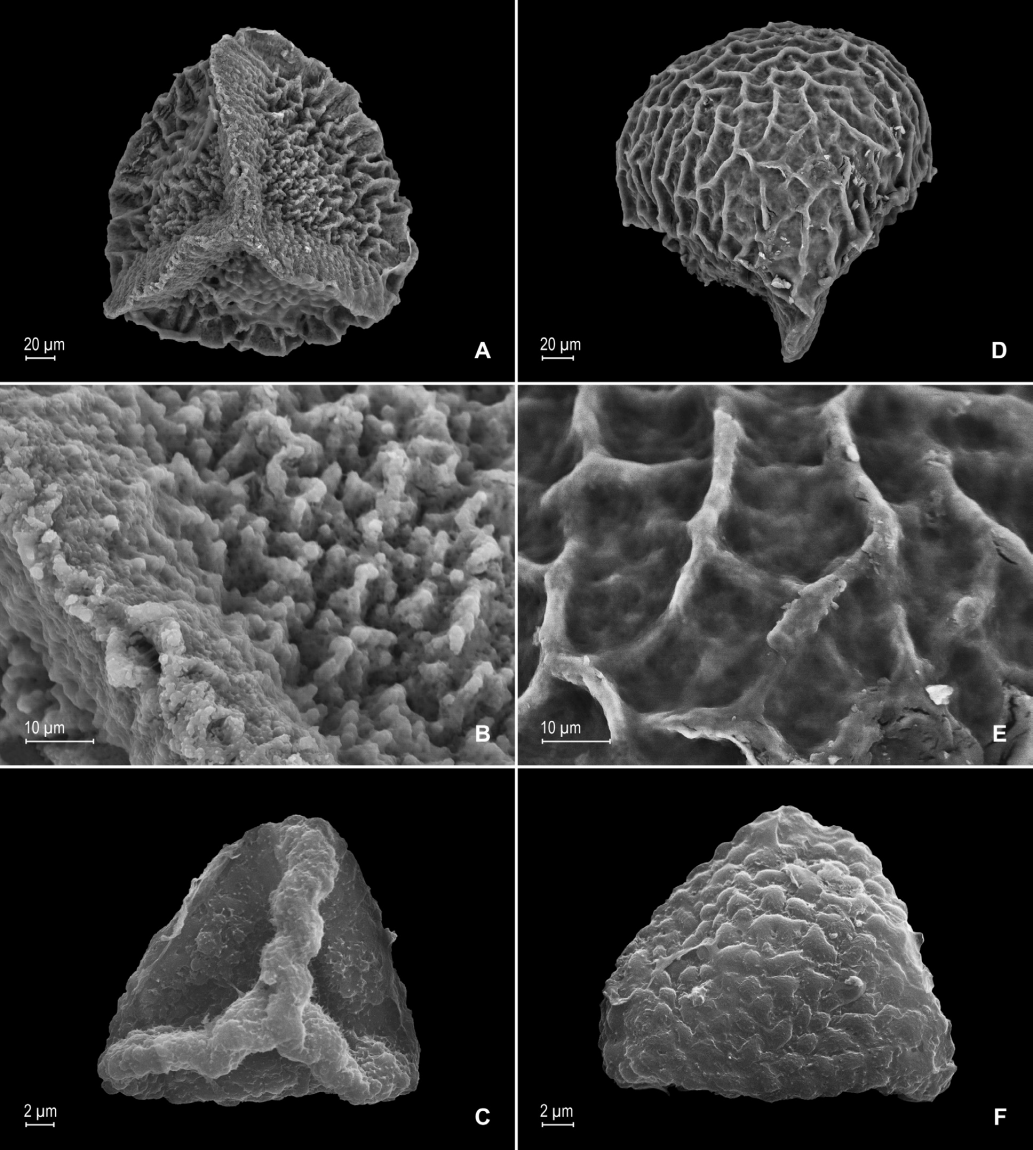
*Selaginella cataniapensis* Valdespino. **A**. Megaspore, proximal face; **B**. Close-up of the megaspore, proximal face (same spore as in **A**); **C**. Microspore, proximal face; **D**. Megaspore, distal-equatorial face; **E**. Close-up of the megaspore, distal-equatorial face (same spore as in **D**); **F**. Microspore, distal face. **A–F** from the paratype, *E. Foldats & J. Velazco 9188*, UC.

Figs 5, 6, 7, 8, 9, 10

#### Diagnosis.

*Selaginella cataniapensis* differs from *S. amazonica* Spring by its chartaceous (vs. coriaceous) leaves, fully heteromorphic 28–45 (vs. 6–10) cm below the first branches, lateral leaves after the first branches ovate-oblong (vs. deltate), with the bases truncate on the central portion and plane toward margins (vs. prominent), the basiscopic bases geniculate (vs. rounded), acute to attenuate (vs. acute) apices, and the lower surfaces smooth to slightly striate (vs. corrugate). It is further distinct by its axillary leaf bases rounded to truncate and plane (vs. subcordate with the inner bases cordate and the outer bases rounded with the central portion truncate and prominently raised).

#### Type.

**Venezuela** • **Amazonas** [**Bolívar**]: Depto. [Mpio.] Atures [Cedeño], Laguna El Sillón y Caño Mariguaca, 78 km NE of Puerto Ayacucho, 05°49'N, 66°50'W, alt. 400 m, Oct. 1989, *A. Fernández, E. Sanoja & M. Yanez 6397* (holotype: UC! [UC1784512]; isotypes: MA-digital image! [Cat. No. MA-01–00732931], VEN [Herb. No. 410705]).

#### Description.

***Plants*** terrestrial. ***Stems*** erect, stramineous, 69–75 cm in height, 2.0–3.5 mm diam., non-articulate, not flagelliform, stoloniferous, 1–4-branched. ***Rhizophores*** axillary, dorsal, ventral, and lateral (often all present on a single individual), borne on the lower-most portion of the stems, stout, 0.4–1.0 mm diam. ***Leaves*** on main stems seemingly monomorphic and strongly appressed along the main stems up to 9–16 cm, thereafter becoming obviously heteromorphic throughout, chartaceous, deltate, 1.7–3.0 × 1.6–2.0 mm; bases truncate and glabrous, without auricles; margins light green, denticulate to serrulate; apices acute to shortly attenuate, 0.2–0.4 mm long, denticulate marginally and tipped by 1–3 teeth; upper surfaces deep green, corrugate to corrugate-striate, the lower surfaces shiny and light green to silvery green, smooth to slightly striate. ***Lateral leaves*** on main stems from 28–45 cm below and above the first branches, spreading and perpendicular at 90°–120° to the stems, distant, oblong or broadly ovate-oblong, 3.9–5.6 × 2.0–2.5 mm; acroscopic bases rounded and basiscopic bases geniculate, glabrous; margins greenish on the upper surfaces, bordered by quadrangular to round, laevigate cells, on the lower surfaces margins greenish, bordered by elongate, sinuate-walled, and laevigate cells and submarginally bordered by a discontinuous band 1–3 cells wide of elongate, hyaline, straight-walled, and papillate idioblasts, dentate to denticulate throughout on acroscopic and basiscopic margins; apices falcate, acute to attenuate, denticulate at tips with 1–3 teeth; upper surfaces comprising irregularly shaped, somewhat roundish, rectangular to quadrangular, sinuate-walled, and laevigate cells with the cell margins bordered by 7–10 pit-like structures, without stomata, the lower surfaces comprising elongate, sinuate-walled, and laevigate cells, with cell margins similar to those of the upper surfaces but with 9–26 pit-like structures, with stomata on 1–7 rows along the midribs on central 4/6 of the leaf laminae. ***Median leaves*** alternate to slightly imbricate distally and ascending, those along the proximal portion of the stems deltate, thereafter broadly ovate, ovate-deltate or ovate-elliptic, 3.0–3.7 × 2.0–2.2 mm; bases obliquely truncate, glabrous, without auricles; upper surface margins greenish and submarginally with an unevenly distributed or interrupted row of elongate, straight-walled, and papillate idioblasts with 4–10 papillae in each cell lumen, denticulate to serrulate, the lower surface margins with evenly distributed, submarginal, elongate, straight-walled, and papillate idioblasts, the papillae solitary on each lumen and intermixed with elongate, straight-walled, and papillate cells in 1–4 rows, each lumen with 3–12 papillae; apices acute to short-attenuate, 0.1–0.4 mm long or 1/6–1/8 the length of the laminae, marginally denticulate, tipped by 1–3 teeth; upper surfaces comprising irregularly quadrangular or elongate, sinuate-walled cells with the margins bordered by 6–10 pit-like structures, intermixed with elongate, straight-walled, and papillate idioblasts, the papillae in 1 row, each cell lumen composed of 2–10 papillae, stomata in 1–4 rows along the distal 1/4–2/3 of the leaf laminae along the midribs and few on proximal most portion of the leaf lamina on the outer base, the lower surfaces comprising elongate, sinuate-walled, and laevigate cells, without elongate idioblasts. ***Axillary leaves*** on the main stems beyond the first branches ovate to ovate-lanceolate, 2.6–4.0 × 1.5–2.2 mm, with the inner and outer leaf halves about the same width; bases rounded to truncate; margins denticulate to serrulate throughout; apices acute, tipped by a small tooth; both surfaces as in the lateral leaves, except the lower surfaces with sparsely distributed, elongate, straight-walled, and papillate idioblasts, the papillae arranged in one row on the cell lumen. ***Strobili*** terminal on branch tips, quadrangular, 0.5–5.0 cm long. ***Sporophylls*** monomorphic, with a laminar flap, with a glabrous keel along the midribs, the dorsal sporophylls ovate to ovate-lanceolate, the ventral sporophylls lanceolate, 1.0–1.5 × 0.5–1.2 mm; bases rounded; margins narrowly hyaline, continuously bordered by a band 2–4 cells wide, the cells elongate, straight-walled, and papillate idiblasts, denticulate to serrulate; apices long-attenuate, each 0.3–0.5 mm long, tipped by 1 or 2 tooth-like projections; ***dorsal sporophylls*** with the upper and lower surfaces as in the vegetative leaves, except for the half that imbricates with the ventral sporophylls that is greenish to silvery hyaline and composed of elongate, straight-walled, and papillate idioblasts; ***ventral sporophylls*** with both surfaces silvery green to hyaline, comprising elongate, sinuate- to straight-walled, and papillate idioblasts. ***Megasporangia*** on two ventral rows or along proximal 2/3 of two ventral rows; ***megaspores*** white, whitish to light cream, the proximal faces rugulate to rugulate-reticulate marginally, reticulae ill-defined, delimited by low ridges, with an equatorial flange, the microstructure smooth, perforate, and foveolate, the distal faces reticulate, reticulae closed and open, delimited by low ridges, each rugose, the microstructure smooth and perforate, 185–226 µm diam. ***Microsporangia*** along two dorsal rows or also on distal 1/3 of two ventral rows; ***microspores*** light orange, the proximal faces rugulate with the microstructure echinate, the distal faces rugulate with the microstructure smooth and with sparsely distributed microechinae, 18–24 µm diam.

#### Habitat and distribution.

*Selaginella cataniapensis* is known from four collections in the Cataniapo river basin in Venezuela (states of Amazonas and Bolívar), where it occurs in lowland tropical wet forest at elevations of 90–600 m. One specimen (*A. Castillo 2046*, MO) is labeled “*Amazonas: Depto. Atures, Puerto Ayacucho, Río Cataniapo, between Saramatosa and Salto Nieve (Iguarimo)*,” which clearly places the collection in Amazonas; however, the coordinates on the label plot it in the state of Apure, suggesting a coordinate or transcription error. Because the locality information is internally consistent and geographically specific, we treat this record as originating from Amazonas and rely on the locality (rather than the mapped label coordinates) for georeferencing.

#### Etymology.

This fern-like species with upright stems is named after the general area where it has been collected so far.

#### Conservation assessment.

*Selaginella cataniapensis* is currently documented from only three collections in Venezuela, from the Cataniapo River Basin. This area has also served as the type locality or name-bearing reference for some flowering plant taxa described from the region, including *Coryanthes cataniapoensis* G.A.Romero & Carnevali [Orchidaceae] (Romero and Carnevali 1989), *Ouratea cataniapoensis* [Ochnaceae] Aymard (Aymard 2018), and *Sloanea cataniapensis* Steyerm. [Elaeocarpaceae] (Steyermark 1988 [1989]). Using GeoCAT, these georeferenced records yield an extent of occurrence (EOO) of 2,068 km^2^ and an area of occupancy (AOO) of 12 km^2^. Following the IUCN Standards and Petitions Committee (2022), we propose a preliminary conservation assessment of Endangered (EN B1ab(iii)+B2ab(iii)) and infer a continuing decline in habitat quality in parts of the Cataniapo basin associated with land-use pressures (e.g., shifting cultivation and riparian/roadside land conversion) (Blanco 2005).

#### Additional specimen examined (paratypes).

**Venezuela** • **Amazonas**: Depto. Atures, 125 km de la boca (delta) del Guayapo en Sipapo, 04°22'N, 67°06'W, alt. 130 m, May 1989, *E. Foldats & J. Velazco 9188* (UC), • Puerto Ayacucho, Río Cataniapo, between Saramatosa and Salto Nieve (Iguarimo), 06°25'N, 67°25'W, alt. 90–100 m, 10 Aug. 1986, *A. Castillo 2046* (MO, VEN); • Serranía Batata, 2 km NE of Salto Colorado, Caño Colorado, 55 km NE of Puerto Ayacucho, 05°33'N, 67°08'W, alt. 550 m, Sep. 1989, *A. Fernández, E. Sanoja & M. Yanez 6359* (MA [digital image], UC, VEN).

#### Discussion.

*Selaginella cataniapensis* is a Venezuelan Guayanan floristic element found at low elevations. It features a fern-like, erect growth form, with quadrangular stems measuring 1.8–2.5 mm in diam. The plant has stout rhizophores that can be ventral, dorsal, axillary, and lateral, confined to the lowermost, creeping stems, each with a diameter of 0.4–1.0 mm. The species can be further identified by glabrous leaves, with lateral leaves oblong or broadly ovate-oblong, positioned perpendicular or nearly so to the stems. The median leaves on branches are ovate to ovate-elliptic, while the axillary leaves range from ovate to ovate-lanceolate with rounded to truncate, glabrous bases. Furthermore, on the main stems, before the first branches, the leaves tend to be reflexed, though this may be a drying artifact.

Specimens of *Selaginella cataniapensis* were originally identified as *S. amazonica*, from which it differs in the characteristics discussed in the diagnosis. *S. cataniapensis* is additionally distinguished from *S. amazonica* by its lateral leaves, which spread throughout the main stem and are perpendicular at a right to obtuse angle of 90°–120° (vs. ascending at a 45° angle). It also has distant (vs. imbricate) lateral leaves with the basiscopic bases geniculate (vs. rounded), margins sparsely denticulate to serrulate (vs. dentate), falcate, acute to attenuate apices (vs. obtuse), and median leaves with acute to short-attenuate (vs. long-attenuate to short-aristate) apices, each 0.1–0.4 mm in length or 1/10 (vs. 1/4) of the leaf lamina length. Additionally, *S. cataniapensis* has its lateral leaf lower surfaces composed of submarginal, elongate, straight-walled, and papillate idioblasts (vs. idioblasts throughout the leaf surface, except submarginally).

*Selaginella cataniapensis* appears morphologically similar to *S. palmiformis* Alston ex Crabbe & Jermy, both of which have submarginal, elongate, straight-walled, and papillate idioblasts along the acroscopic and basiscopic margins of lateral and median leaves. *Selaginella cataniapensis* differs from *S. palmiformis* by its median leaves after the first branches, broadly ovate, ovate-deltate or ovate-elliptic (vs. ovate) with the bases rounded to truncate, plane in outline, and glabrous (vs. with the inner base truncate and the outer base oblique with a small, short-ciliate knob or reduced auricle), and with apices acute to short-attenuate and 1/8 (vs. long-attenuate, at least 1/4) the length of the lamina. Additionally, *S. cataniapensis* differs from *S. palmiformis* by its oblong or broadly ovate-oblong (vs. ovate-oblong) lateral leaves with apices falcate, acute to attenuate, each 1/16 the length of the lamina (vs. broadly acute). It is further distinguished from *S. palmiformis* by its megaspores distal faces reticulate with close reticulae, with each reticulum ridge and lumen microrugose, perforate, and foveolate (vs. reticulate with close reticulae, with each reticulum ridge and lumen microechinate and perforate), and microspores distal faces rugulate with microstructure smooth and sparsely microechinate (vs. rugulate and densely microechinate).

### Selaginella cultellifolia

Taxon classificationPlantaeSelaginellalesSelaginellaceae

Valdespino & C.López sp. nov.

2F5973DF-9049-5FE6-BBD0-535DCBAFAA57

urn:lsid:ipni.org:names:77381370-1

Figs 11, 12, 13, 14

#### Diagnosis.

*Selaginella cultellifolia* differs from *S. imbricans* A.R.Sm. by its lateral leaves narrowly oblong-ovate (vs. oblong), the acroscopic margins long-ciliate along proximal 1/2, denticulate on distal 1/2 (vs. denticulate throughout), the basiscopic margins long-ciliate along proximal-most 1/8–1/4, entire to sparsely dentate along central 1/4–1/2, and denticulate on distal 1/2 (vs. entire), gradually tapering into a falcate and short-acuminate (vs. abruptly tapering into falcate and broadly acute) apices, and the median leaves broadly elliptic (vs. lance-elliptic or narrowly ovate-elliptic).

**Figure 11.**
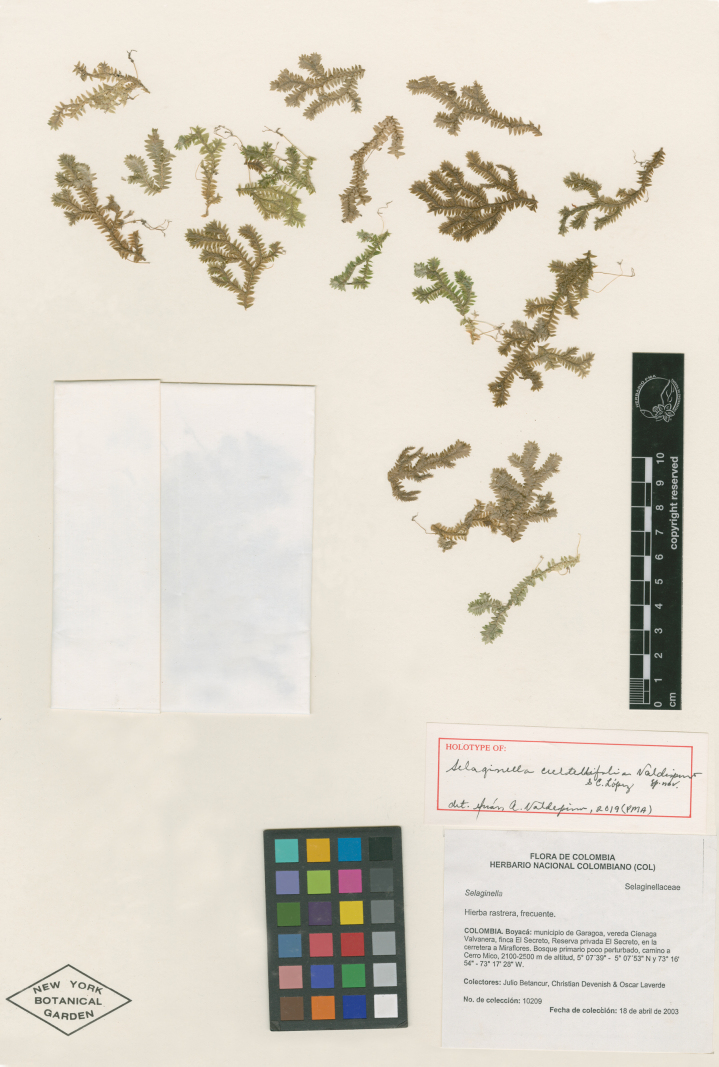
Holotype of *Selaginella cultellifolia* Valdespino & C.López. Digitized image courtesy of the herbarium of the University of Panama (PMA), from a loaned specimen from the herbarium of the New York Botanical Garden, *J. Betancur et al. 10209*, NY.

**Figure 12.**
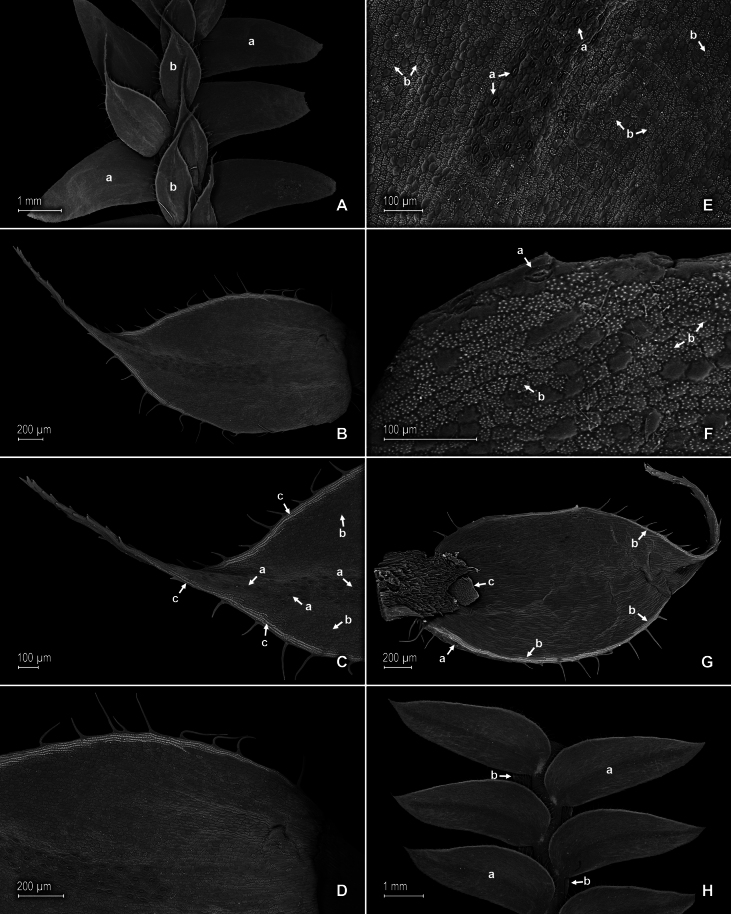
*Selaginella cultellifolia* Valdespino & C.López. **A**. Section of the stem, upper surface; note lateral (a) and median (b) leaves; **B**. Median leaf, upper surface; **C**. Close-up of the distal portion and apex of the median leaf, upper surface (same leaf as in **B**); note stomata along the midrib (a), quadrangular to rounded, papillate cells on the leaf lamina (b) and marginal, elongate, straight-walled, and papillate idioblasts (c); **D**. Close-up of the proximal portion and the inner base of the median leaf, upper surface (same leaf as in **B**); note cells as in **C**; **E**. Close-up of the mid-section of the median leaf, upper surface (same leaf as in **B**); note stomata along the midrib (a) and quadrangular to rounded, papillate cells (b); **F**. Close-up of the proximal, outer base section of the median leaf, upper surface (same leaf as in **B**); note submarginal to marginal stomata (a) and quadrangular to rounded and papillate cells (b); **G**. Median leaf, lower surface; note submarginal stomata (a), elongate, straight-walled, and papillate idioblast along margins (b), and the ligule (c); **H**. Section of the stem, lower surface; note lateral (a) and median (b) leaves. **A–H** from the holotype, *J. Betancur et al. 10209*, NY.

**Figure 13.**
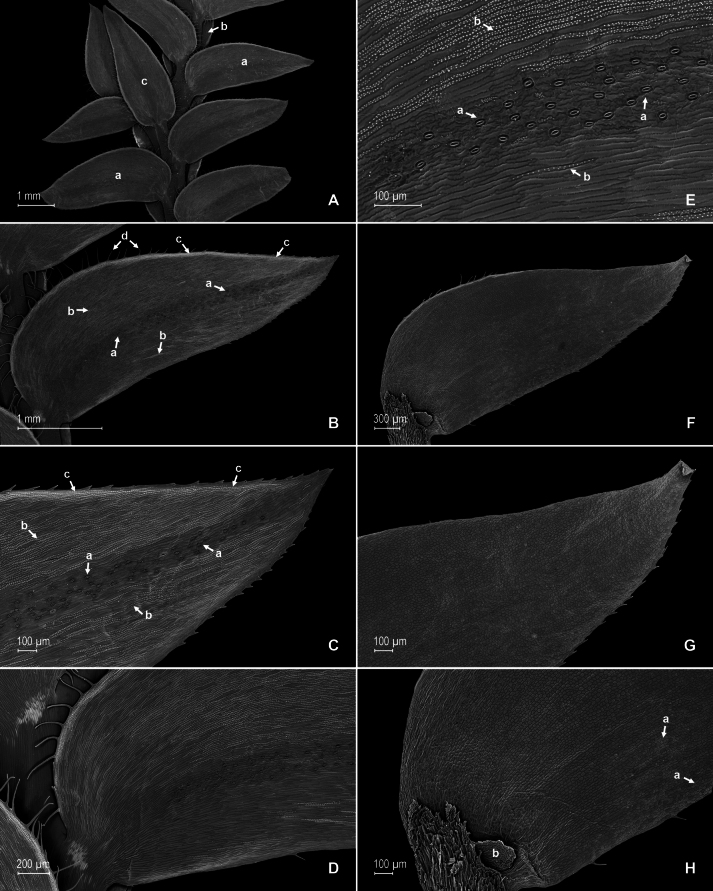
*Selaginella cultellifolia* Valdespino & C.López. **A**. Section of the stem, lower surface; note lateral (a), median (b), and axillary (c) leaves; **B**. Lateral leaf, lower surface; note stomata along the midrib (a), elongate, straight-walled, and papillate idioblasts on the leaf lamina (b) and margins (c), and long-ciliate acroscopic margin (d); **C**. Close-up of the distal portion and apex of the lateral leaf, lower surface (same leaf as in **B**); note stomata along midrib (a), elongate, straight-walled, and papillate idioblasts on leaf lamina (b), and along the acroscopic margin (c); **D**. Close-up of the proximal portion and base of the lateral leaf, lower surface (same leaf as in **B**); note stomata, idioblasts on cell lamina and on the acroscopic margins, and marginal cilia as in **B**; **E**. Close-up of the mid-section of the lateral leaf, lower surface (same leaf as in **B**); note stomata along the midrib (a) and elongate, straight-walled, and papillate idioblasts on the leaf lamina (b); **F**. Lateral leaf, upper surface; **G**. Close-up of distal portion and apex of the lateral leaf, upper surface (same leaf as in **F**); **H**. Close-up of the proximal portion and base of the lateral leaf, upper surface (same leaf as in **F**); note quadrangular, papillate cells on the leaf lamina (a) and the ligule (b) at base. **A–H** from the holotype, *J. Betancur et al. 10209*, NY.

**Figure 14.**
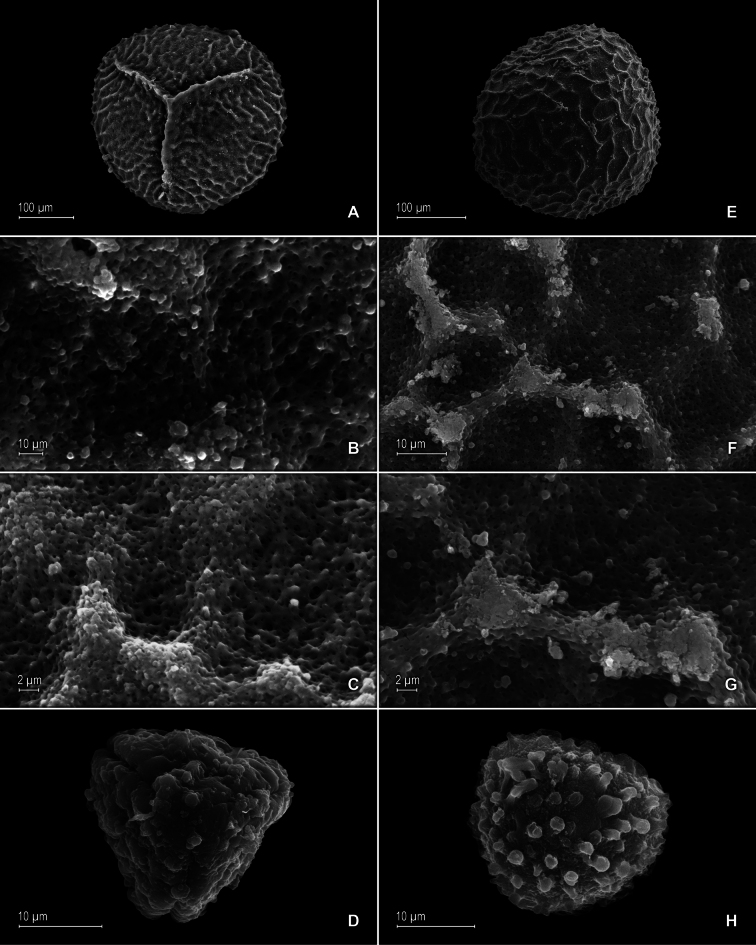
*Selaginella cultellifolia* Valdespino & C.López. **A**. Megaspore, proximal face; **B**. Close-up of the megaspore, proximal face (same spore as in **A**); **C**. Close-up of the megaspore, proximal face (same face as **B**); **D**. Tetrad of microspores, equatorial and distal faces; **E**. Megaspore, distal face; **F**. Close-up of the megaspore, distal face (same spore as in **E**); **G**. Close-up of the megaspore, distal face (same face as **F**); **H**. Microspore, distal face. **A–H** from the holotype, *J. Betancur et al. 10209*, NY.

#### Type.

**Colombia** • **Boyacá**: Mpio. Garagoa, vereda Cínaga Valvanera, finca El Secreto, Reserva Privada El Secreto, road Miraflores, camino a Cerro Mico, 05°07'39"N, 73°16'54"W–73°17'28"W, alt. 2100–2500 m, 18 Apr. 2003, *J. Betancur, C. Devenish & O. Laverde 10209* (holotype: NY!; isotypes: COL-digital image!, PMA!).

#### Description.

***Plants*** terrestrial. ***Stems*** creeping, stramineous, to 12 cm long, 0.5–0.8 mm diam., non-articulate, not flagelliform or stoloniferous, 2-branched. ***Rhizophores*** axillary, borne throughout proximal 1/2 of the stems, filiform, (0.1) 0.2–0.4 mm diam. ***Leaves*** heteromorphic throughout, chartaceous, both surfaces glabrous, the upper surfaces green, the lower surfaces silvery green. ***Lateral leaves*** imbricate to distant, spreading, narrowly oblong-ovate, 2.5–4.3 × 0.8–1.6 mm; bases asymmetric, the acroscopic bases rounded and strongly overlapping the stems, the basiscopic bases oblique to rounded and free from the stems; acroscopic margins on both surfaces hyaline, continuously bordered by a band 2–5 cells wide of idioblasts, the idioblasts elongate, straight-walled, and papillate, the papillae in 1 or 2 rows over each cell lumen, long-ciliate along proximal 1/2 and denticulate on distal 1/2, the basiscopic margins on the upper surface greenish to slightly hyaline, bordered by an irregularly distributed band 1–4 cells wide, the cells quadrangular, straight-walled, and papillate, each cell lumen covered by 5–20 papillae, on the lower surfaces greenish, bordered by elongate, straight-walled, laevigate cells, long-ciliate along proximal most 1/8–1/4, with each cilium 0.1 to 0.2 mm long, entire to sparsely dentate along central 1/4–1/2, and denticulate on distal 1/2; apices gradually tapering, falcate, and short-acuminate, the acumen 0.1 mm long, each tipped by 1–3 teeth; upper surfaces comprising quadrangular to rounded, sinuate-walled, and laevigate cells intermixed with similarly shaped, papillate cells, sparsely distributed along the distal 1/2 of the acroscopic half of the leaf lamina and along the distal 3/4 of the basiscopic half of the leaf lamina, each cell lumen covered by 10–25 papillae, the lower surfaces comprising elongate, sinuate-walled, and laevigate cells, and elongate, straight-walled, and papillate idioblasts distributed on both sides of the midribs, the papillae mostly in 2 rows over each cell lumen, with stomata in 4 rows along distal 7/8 of the midribs, where the cells are laevigate, or the stomata only in 1 or 2 rows distally. ***Median leaves*** imbricate, ascending, ovate to ovate-elliptic, 1.5–2.0 × 0.6–1.0 mm; bases rounded to oblique, the outer bases tufted with 3–6 long cilia, each 0.1–0.3 mm long; the inner margins and the distal 3/4 of the outer margins hyaline, bordered by a band 3–5 cells wide of idioblasts, the idioblasts elongate, straight-walled, and papillate parallel to margins, papillae in 1 or 2 rows over each cell lumen, the inner margins long-ciliate throughout and the outer margins entire along proximal 1/4 and long-ciliate along distal 3/4, with the cilia sparse, each 0.1–0.3 mm long, the proximal 1/4 of the outer margins greenish with the cells elongate, sinuate-walled, and laevigate; apices gradually tapering into a long arista, each arista 0.5–1.2 mm long, with margins denticulate and tipped by 2–4 teeth or short cilia; both surfaces without idioblasts, the upper surfaces comprising quadrangular to rounded, sinuate-walled cells, most of these covered by 6–16 papillae, with stomata in 4 rows along distal 2/3 of the midribs and few on proximal 1/4 of the outer margins, the lower surfaces comprising elongate, sinuate-walled cells, without stomata. ***Axillary leaves*** lanceolate or ovate-lanceolate, 2.4–3.6 × 1.0–1.8 mm, with the inner and the outer leaf halves about the same width; bases rounded; margins long-ciliate along proximal 1/2–2/3, otherwise dentate to denticulate distally; apices acute, tipped by 1–3 tooth-like projections; both surfaces as in the lateral leaves. ***Strobili*** terminal and semi-sessile, and lateral on branches, compact, mostly quadrangular, 0.4–30 mm long. ***Sporophylls*** monomorphic, without a laminar flap, with a glabrous keel along the midribs, lanceolate to lance-ovate, 1.3–1.9 × 0.5–0.8 mm; bases rounded; margins hyaline bordered by elongate, straight-walled, and papillate cells, this more obviously so on dorsal sporophylls, sparsely dentate to denticulate; apices long-attenuate, each 0.3–0.6 mm long, with the upper surface with tooth-like hairs and the lower surfaces glabrous, and tipped by 1–3 teeth; ***dorsal sporophylls*** with the upper surfaces green and cells as in the median leaves, except for the half that imbricates with ventral sporophylls, which is greenish to silvery hyaline and comprising elongate cells, the lower surfaces silvery green and comprising elongate, sinuate-walled cells; ***ventral sporophylls*** with both surfaces hyaline, comprising elongate, sinuate-walled cells. ***Megasporangia*** in two ventral rows; ***megaspores*** white, the proximal faces rugulate, with a slightly developed equatorial flange, the microstructure gemmate and perforate to foveolate, the distal faces reticulate with closed and open reticulae, the microstructure gemmate, perforate to foveolate, and echinate, 260–350 µm diam. ***Microsporangia*** few in two proximal dorsal rows, with the rest of the sporophylls without sporangia or proximally intermixed with one row of microsporangia and the other row occupied by megasporangia, otherwise distally without sporangia; ***microspores*** light-orange or tan-colored, with the proximal faces rugulate, with the microstructure laevigate, the distal faces rugulate, or baculate to capitate, with the microstructure laevigate and slightly echinate, 14–22 µm diam.

#### Habitat and distribution.

*Selaginella cultellifolia* is known from Colombia and Venezuela. It grows in primary montane, cloud forests at 1300–2500 m.

#### Etymology.

Derived from the Latin words “*cultellus*,” a diminutive of knife, and “*folium*,” meaning leaf; together, these allude to the lateral leaf shape that resembles a knife.

#### Conservation assessment.

*Selaginella cultellifolia* is known from only two georeferenced collections, one from Colombia (Boyacá) and another from Venezuela (Barinas). These records yield an area of occupancy (AOO) of 8 km^2^. The extent of occurrence (EOO) was not estimated because fewer than three georeferenced occurrences are available. Given its very small AOO and occurrence at very few locations, we propose a preliminary conservation assessment of Critically Endangered [CR B2ab(iii)].

#### Additional specimen examined (Paratype).

**Venezuela** • **Barinas**: Distr. Bolivar, between Santo Domingo y Altamira, Quebradón, road to the tunnel to the dam, [ca. 08°55'10.95"N, 70°11'19.68"W], alt 1300–1500 m, 24 Nov. 1984, *F. Ortega & H. van der Werff 2360* (UC).

#### Discussion.

*Selaginella cultellifolia* is characterized by its creeping stems, axillary and filiform rhizophores borne along the proximal 1/2 of the stems, median leaves ovate to ovate-elliptic, with the inner margins and the distal 3/4 of the outer margins hyaline, bordered by a band 3–5 cells wide of elongate, straight-walled, and papillate idioblasts, margins long-ciliate, and long-aristate apices with the arista 0.5–1.2 mm in length and tipped by 2–4 teeth or short cilia.

*Selaginella cultellifolia* is morphologically similar to *S. calosticha* Spring and *S. imbricans* because they share a creeping or sometimes ascending growth habit with axillary rhizophores, similar leaf shapes, median leaves with long-aristate apices, strobili borne almost laterally on the branches, and white to light-cream megaspores. However, it differs from *S. calosticha* by its narrowly oblong-ovate (vs. narrowly oblong) lateral leaves, with the acroscopic margins long-ciliate along the proximal 1/2 and denticulate on the distal 1/2 (vs. dentate to denticulate along the proximal 1/4–1/2, otherwise entire distally). Additionally, its basiscopic margins are long-ciliate along the proximal-most 1/8–1/4 portion of the margins, entire to sparsely dentate along the central 1/4–1/2, and denticulate on distal 1/2 (vs. entire throughout or denticulate along distal 1/3), with short-acuminate (broadly acute to obtuse) apices, and filiform (vs. stout) rhizophores, each (0.1) 0.2–0.4 (vs. 0.4–0.6) mm in diam.

*Selaginella cultellifolia* is distinguished from *S. imbricans* by the characters outlined in the diagnosis, and by its median leaves inner margins long-ciliate throughout and the outer margins entire along proximal 1/4 and long-ciliate along distal 3/4 (vs. the inner margins dentate and the outer margins dentate throughout or dentate along proximal 1/2 and denticulate along distal 1/2), with long-aristate (vs. short- to long-aristate) apices, the arista 1/3–1/2 (vs. 1/3 or less) the length of the lamina or 0.5–1.2 (vs. 0.3–0.6) mm long. Additionally, its lateral leaves have basiscopic margins that are long- (vs. short-) ciliate along the proximal 1/8–1/4, with each cilium measuring 0.1 to 0.2 (vs. 0.05–0.1) mm long. *Selaginella cultellifolia* differs further from *S. imbricans* by having the proximal 1/2 of the lateral leaves that are wider than the distal 1/2 (vs. both halves are nearly equal in width) and white (vs. cream to cream-yellow) megaspores.

### Selaginella guaramacalensis

Taxon classificationPlantaeSelaginellalesSelaginellaceae

Valdespino & C.López sp. nov.

23909D67-BB5D-5916-92BC-292905B6ECB5

urn:lsid:ipni.org:names:77381372-1

Figs 15, 16, 17

#### Diagnosis.

*Selaginella guaramacalensis* differs from *S. substipitata* Spring by its median leaves broadly elliptic to broadly ovate-elliptic (vs. elliptic) with the outer bases tufted with 5–17 long cilia (vs. glabrous), the outer margins long-ciliate along distal 3/4 (vs. dentate to serrate throughout), the lateral leaves acroscopic margins on the lower surface broadly hyaline, bordered by 6–18 (vs. 3–5) rows of elongate, straight-walled, and papillate idioblasts, with broadly-acute to obtuse (vs. acute) apices.

**Figure 15.**
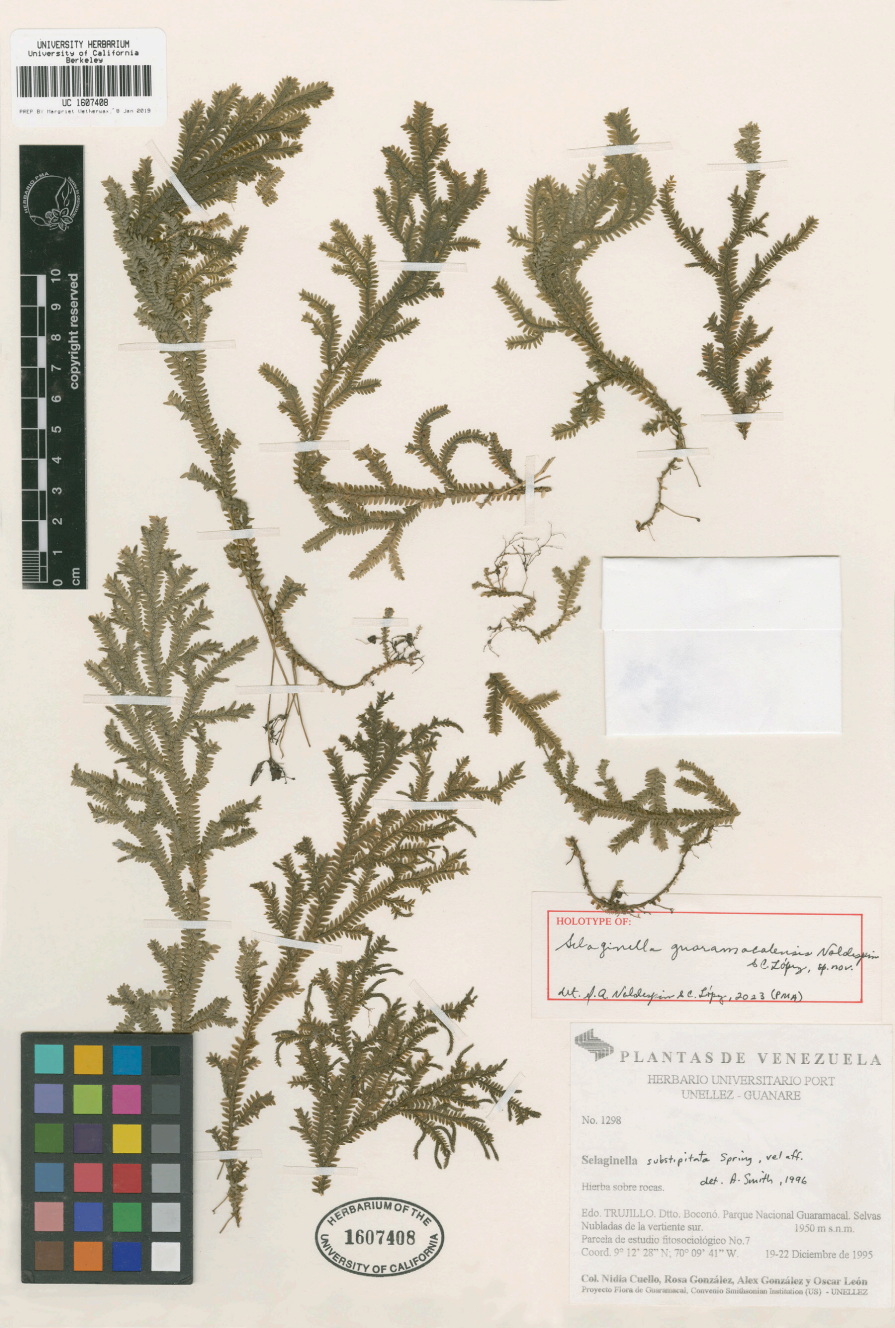
Holotype of *Selaginella guaramacalensis* Valdespino & C.López. Digitized image courtesy of the herbarium of the University of Panama (PMA), from a loaned specimen from the herbarium of the University of California, *N. Cuello et al. 1298*, UC.

**Figure 16.**
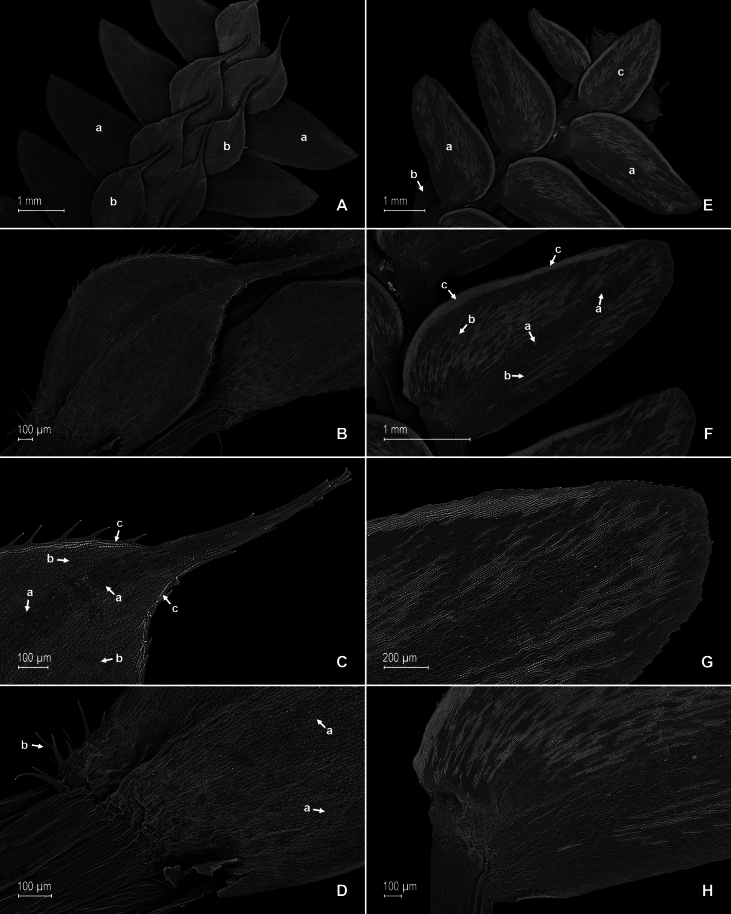
*Selaginella guaramacalensis* Valdespino & C.López. **A**. Section of the stem, upper surface; note lateral (a) and median (b) leaves; **B**. Median leaf, upper surface; **C**. Close-up of the distal portion and apex of the median leaf, upper surface (same leaf as in **B**); note stomata along the midrib (a), quadrangular and papillate cells on leaf lamina (b), and distal, marginal, elongate, straight-walled, and papillate idioblasts (c); **D**. Close-up of the proximal portion and base of the median leaf, upper surface (same leaf as in **B**); note quadrangular and papillate cells on the leaf lamina (a), and outer base tufted with long cilia (b); **E**. Section of the stem, lower surface; note lateral (a), median (b), and axillary (c) leaves; **F**. Lateral leaf, lower surface; note stomata along the midrib (a), elongate, straight-walled, and papillate idioblasts throughout the lamina surface (b) and along acroscopic margin (c); **G**. Close-up of the distal portion and apex of the lateral leaf, lower surface (same leaf as in **F**); note stomata and idioblasts as in **F**; **H**. Close-up of the proximal portion and base of the lateral leaf, lower surface (same leaf as in **F**); note stomata and idioblasts as in **F**. **A–H** from the holotype, *N. Cuello et al. 1298*, UC.

**Figure 17.**
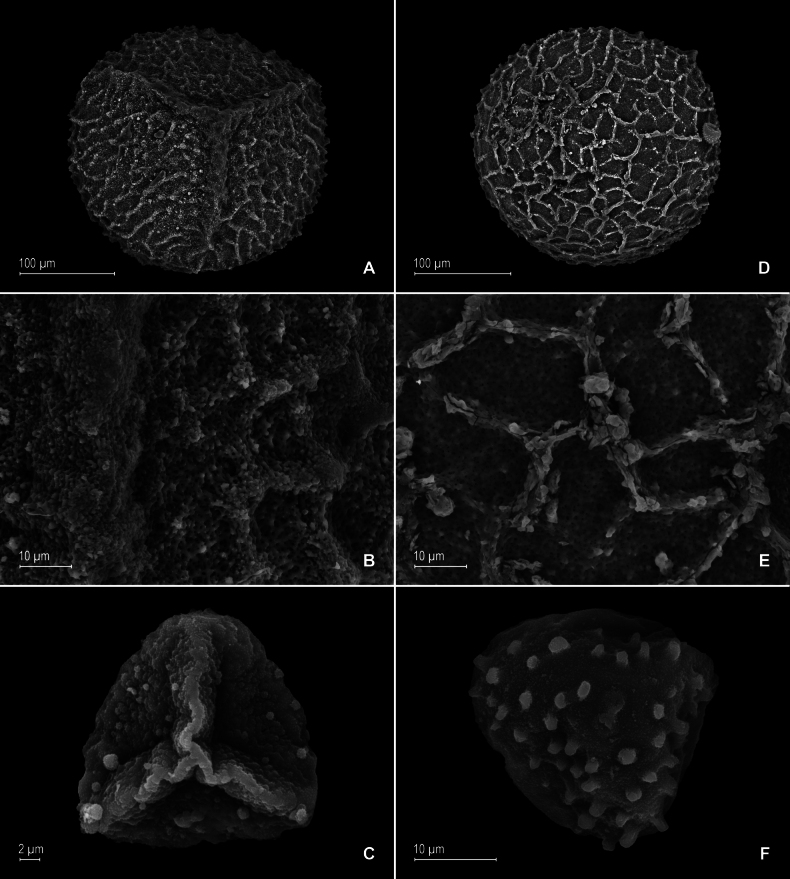
*Selaginella guaramacalensis* Valdespino & C.López. **A**. Megaspore, proximal face; **B**. Close-up of the megaspore, proximal face (same spore as in **A**); **C**. Microspore, proximal face; **D**. Megaspore, distal face; **E**. Close-up of the megaspore, distal face (same spore as in **D**); **F**. Microspore, distal face. **A–F** from the holotype, *N. Cuello et al. 1298*, UC.

#### Type.

**Venezuela** • **Trujillo**: Dto. Boconó, Parque Nacional Guaramacal, 09°12'28"N, 70°09'41"W, alt. 1950 m, 19–22 Dec. 1995, *N.L. Cuello*, *R. González, A. González & O. León 1298* (holotype: US! [US00513861]; isotypes: MO! [MO-2012938], NY!, PORT-n.v., UC! [UC1607408]).

#### Description.

***Plants*** terrestrial or epipetric. ***Stems*** decumbent to ascending, stramineous, 15–30 cm long, 0.7–2.0 mm diam., non-articulate, not flagelliform or stoloniferous, 2- or 3-branched. ***Rhizophores*** axillary, along the proximal 1/3–1/2 portion of the stems, stout, 0.4–1.0 mm diam. ***Leaves*** heteromorphic, chartaceous, both surfaces glabrous, the upper surfaces bright green to yellowish-green, smooth to slightly corrugate, the lower surfaces shiny green to yellowish-green (however, after specimens’ preservation, both surfaces become brownish). ***Lateral leaves*** on the main stems perpendicular at 90° to the stems, distant, oblong or oblong-ovate, 3.3–5.0 × 1.3–2.0 mm; bases rounded to rounded-truncate, the acroscopic bases strongly overlapping the stems, the basiscopic bases free from the stems; acroscopic margins on the upper surfaces greenish, composed of rounded to quadrangular, laevigate cells, on the lower surfaces broadly hyaline, continuously bordered by a band 6–18 cells wide of idioblasts, each elongate, straight-walled, and papillate, the papillae in one row on each cell lumen, serrate to dentate throughout or entire on proximal 2/12 and serrate to dentate on distal 10/12, or short-ciliate along proximal 2/12 and serrate to dentate on distal 10/12, the basiscopic margins on both surfaces greenish, continuously bordered by rounded to quadrangular cells, on the upper surfaces with some cells papillate, on the lower surfaces with laevigate cells, entire along proximal 3/4, sparsely denticulate on distal 1/4; apices broadly-acute to obtuse, tipped by 3–6 teeth; upper surfaces composed of irregularly shaped, somewhat roundish, rectangular to quadrangular, sinuate-walled, laevigate cells, some cells papillate and sparsely distributed on the leaf laminae, and with some papillate cells along a distinct submarginal and marginal band, without stomata, the lower surfaces composed of elongate, sinuate-walled, and laevigate cells and of elongate, straight-walled, and papillate idioblasts throughout the acroscopic half of the leaf lamina and on the basiscopic half along distal 3/4, stomata on 1–4 rows along the midrib on the central 2/3 of the leaf lamina. ***Median leaves*** slightly imbricate, ascending, broadly elliptic to broadly ovate-elliptic, 1.0–2.4 × 1.0–1.5 mm; bases plane in side view, oblique to oblique-rounded with the inner bases oblique and glabrous, and the outer bases, rounded and tufted with a ciliate knob or auricula, each with 5–17 long cilia, each 45–150 µm long; inner margins greenish along proximal 1/4, narrowly hyaline along distal 3/4 and continuously bordered by a band 1–4 cells wide of idioblasts, the idioblasts elongate, straight-walled, and papillate, the papillae in one row on each cell lumen, entire along proximal 1/3, short-ciliate, serrate or dentate along distal 2/3, the outer margins greenish along proximal 1/4, otherwise similarly hyaline as on the inner margins, entire along proximal 1/4, otherwise short- to long-ciliate along distal 3/4; apices long-aristate, 1/3–3/4 or more the length of the lamina, each arista 0.9–1.2 mm long, tipped by 1–5 teeth; the upper surfaces comprising quadrangular to rounded, sinuate-walled cells, most of these covered by 6–14 papillae, with stomata in 2–4 rows along distal 1/2 of the midribs and on proximal 1/2 of the outer margins, the lower surfaces comprising elongate, sinuate-walled cells and with few, sparsely distributed, elongate, straight-walled, and papillate idioblasts, the idioblasts on one row along the cell lumen, without stomata. ***Axillary leaves*** ovate to ovate-lanceolate, 2.0–3.9 × 1.0–1.8 mm; bases rounded, glabrous; margins on the upper and lower surfaces similar to the acroscopic margins on the upper and the lower surfaces of the lateral leaves, sparsely, long-ciliate along the proximal-most portion, otherwise, entire to denticulate distally; apices acute to broadly acute, similarly tipped as in lateral leaves. ***Strobili*** terminal, single or bifurcate on branches, 4.0–9.0 mm long. ***Sporophylls*** monomorphic, without a laminar flap, ovate to ovate-lanceolate, 1.2–1.6 × 0.5–0.7 mm, each with a strongly developed and dentate keel along the distal 1/4–1/3 of the midribs on the dorsal sporophylls and entire or sparsely denticulate on the distal 1/3 of the ventral sporophylls; bases rounded; margins narrowly hyaline, dentate to denticulate; apices long-acuminate, each acumen 0.3–0.5 mm long with the margins denticulate and tipped by 1–3 teeth; ***dorsal sporophylls*** with the upper surfaces green and cells as in the median leaves, the lower surfaces silvery green and comprising elongate, sinuate-walled cells; ***ventral sporophylls*** with both surfaces hyaline, comprising elongate, sinuate-walled cells. ***Megasporangia*** along the distal 1/2 of two ventral rows; ***megaspores*** light yellow, reticulate-rugulate on the proximal faces with closed reticula, without a prominent equatorial flange, the microstructure perforate, the distal faces reticulate with closed reticula, and the microstructure perforate, 250–280 µm diam. ***Microsporangia*** in two dorsal rows and proximal 1/2 of two ventral rows; ***microspores*** yellow, the proximal faces rugulate, the microstructure echinate, and the distal faces baculate with the microstructure of the bacula and the rest of the surfaces echinate, 22–30 µm diam.

#### Habitat and distribution.

*Selaginella guaramacalensis* grows as an understory terrestrial or epipetric plant in humid, shaded rainforests at elevations of 1000–1950 m in and around Parque Nacional Guaramacal, located in the Serranía de Guaramacal within the municipalities of Boconó and Sucre, in the states of Portuguesa and Trujillo, Venezuela.

#### Etymology.

The specific epithet refers to the Venezuelan Parque Nacional Guaramacal, where the species has been collected.

#### Conservation assessment.

*Selaginella guaramacalensis* is known only from the immediate area of Parque Nacional Guaramacal (Parque Nacional “General Cruz Carrillo”) in the Serranía/Ramal de Guaramacal, an outlier of the Cordillera de Mérida on the northeastern margin of the Venezuelan Andes (Dorr et al. 2000; Cuello et al. 2010; Dorr and Niño 2024; BirdLife International 2024). Based on 6 georeferenced occurrences, the Extent of Occurrence (EOO) of *S. guaramacalensis* is 1,274.218 km^2^, and the Area of Occupancy (AOO) is 24 km^2^. The species is known from a few localities in Trujillo, with a small extension into Portuguesa, and occurs in montane wet forest/cloud forest habitats. Although the park has been described as generally well conserved, it is not free from human impacts. Reported threats include resource extraction and agricultural activities in adjacent areas, as well as a proposed nearby gas development and associated infrastructure/road improvements that could increase access and pressure on less-disturbed sectors of the park (Muñ̃oz et al. 2006; BirdLife International 2024). Agriculture is reported to be non-extensive within the park, but land-use change around the park contributes to deforestation and habitat fragmentation along its boundaries (Muñoz et al. 2006; BirdLife International 2024). Consequently, despite occurring within a protected area, a continuing decline in habitat quality can be inferred, and we therefore provisionally assess the species as Endangered (EN) under criterion B1ab(iii)+B2ab(iii).

#### Additional specimens examined (paratypes).

**Venezuela** • **Portuguesa**: Dist. Sucre, La Divisoria de la Concepción, 09°18'N, 70°06'W, alt. 1500–1800 m, 23–26 Oct. 1985, *F. Ortega et al. 2731* (MO, PORT-n.v., UC), *F. Ortega et al. 2761* (MO, PORT-n.v., UC); • Los Paramitos, 20 km by air SW of Biscucuy, 09°20'N, 69°05'W, alt. 1000–1500 m, 17 Sep. 1983, *F. Ortega, B. Stergios & G. Aymard 1839* (UC, VEN); • Parque Nacional Guaramacal, Sector El Paramito, Camino Real Paramito - Batatal, 09°19.3'N, 70°4.25'W & 09°20.35'N, 70°4.8'W, alt. 1550–1950 m, 17 Mar. 1999, *N. Cuello et al. 1472* (US, VEN). • **Trujillo**. Dist. Boconó, above Escuque, between Escuque and La Mesa de San Pedro, [ca. 09°16'44.0"N–09°17'49.7"N, 70°40'21.72"W–70°43'25.00"W], alt. 1300–1650 m, 20–23 Feb. 1971, *J.A. Steyermark 104625* (MO, NY, VEN).

#### Discussion.

*Selaginella guaramacalensis* is distinguished by its decumbent to ascending stems with axillary, stout rhizophores along the proximal 1/3–1/2 portion of the stems. Its median leaves outer bases are tufted with 5–17 long cilia, with the outer margins along the proximal 1/2 greenish, entire, and bordered by stomata, while the distal 1/2 is broadly hyaline, composed of 2–5 rows of elongate, straight-walled, and papillate idioblasts, and with short- to long-ciliate apices. The inner margins of the median leaves are entire along the proximal 1/2 and on the distal 1/2 narrowly bordered by 1–3 rows of elongate, straight-walled, and papillate idioblasts, and serrate to short-ciliate. It is further characterized by its lateral leaf acroscopic margins on the lower surfaces broadly hyaline, bordered by 6–14 rows of elongate, straight-walled, and papillate idioblasts, the leaf lamina covered by elongate, straight-walled, and papillate idioblasts throughout the acroscopic half of the leaf lamina and on the basiscopic half along distal 3/4, and apices broadly acute to obtuse. It is further distinguished by its megasporangia on two ventral rows along the distal 1/2 of the strobili, light-yellow megaspores with open reticulate to reticulate-rugulate on the proximal faces and close reticulate on the distal faces, and microspores rugulate on the proximal faces and baculate on the distal faces.

Specimens of *S. guaramacalensis* were previously identified as *S. aff. producta* Baker and *S. substipitata*, both of which are creeping or ascending species found in Venezuela. For example, the type collection, *Cuello et al. 1298* (NY, PORT, UC, US), was listed as *S. substipitata*, while *Cuello et al. 1472* (PORT, US), *Ortega et al., 2731* (MO, PORT), and *Ortega et al. 2761* (MO, PORT) were listed as *S. aff. producta* (Dorr et al. 2000: 15). Similarly, *Cuello et al. 1298* at US was also identified as a new species with the unpublished herbarium epithet “*stergiosii*,” attributed to Alan R. Smith. We do not adopt this latter name, as it is not included in Smith (1985) or in his subsequent publications on *Selaginella* from Venezuela. Instead, we propose the new species name *S. guaramacalensis* to prevent further confusion and acknowledge the region where this species occurs. *Selaginella guaramacalensis* mainly differs from *S. producta* by its median leaves with the upper surfaces lacking (vs. with) elongate idioblasts, and long-aristate apices, each 1/3–3/4 or more (vs. 1/4) the length of the lamina, axillary leaf bases rounded (vs. cordate), and lateral leaf apices broadly-acute to obtuse (vs. rounded, truncate, truncate-oblique, or broadly acute). Furthermore, specimens of *S. producta* are known to display a bluish-green, greenish-brown, reddish-brown, or red color on the upper leaf surface, in addition to green. However, these color variations have not been reported in *S. guaramacalensis*.

*Selaginella guaramacalensis* is distinguished from *S. substipitata* by the morphological characters discussed in the diagnosis. It also differs from *S. substipitata* in having megasporangia located along the distal half of the two ventral rows, rather than throughout. Additionally, *S. guaramacalensis* has so far only been found in the northwestern part of Venezuela, specifically in the Serranía de Guaramacal at the eastern end of the Andes Mountain range in the states of Trujillo and Portuguesa, whereas *S. substipitata* is known to occur in the northern, central, and southern regions of the country.

*Selaginella guaramacalensis* appears morphologically and geographically close to *S. meridensis* Alston. Both species share similar habits and overall megaspore and microspore coloration and ornamentation. Alston et al. (1981: 267) considered the ornamentation of the microspores’ distal faces of *S. meridensis* to be papillate; however, our careful examination of the SEM image of these spores on the holotype (i.e., *J.A. Steyermark 55791*) at BM revealed that they are shortly baculate, making them similar to the ones depicted here for *S. guaramacalensis* (Fig. 17). The morphological similarities between these two taxa initially led us to determine two of the paratypes of *S. guaramacalensis* (i.e., *F. Ortega et al. 2731 & 2761* at UC) as *S. meridensis*. However, after further reexamination of this material and additional type material cited here, we are now inclined to treat these two taxa as separate species.

*Selaginella guaramacalensis* is distinguished from *S. meridensis* by its smooth to slightly corrugate (vs. corrugate) leaves on the upper surface of the leaf lamina, lateral leaves oblong to oblong-ovate (vs. ovate-lanceolate, ovate, or ovate-oblong), smaller (vs. larger), each 3.5–4.4 (vs. 5.0–7.0) mm long with the acroscopic margins serrate to dentate (vs. entire to denticulate or long-ciliate along proximal 1/8) and on the lower surfaces broadly (vs. narrowly) hyaline, bordered by 6–18 (vs. 1–4) rows of elongate, straight-walled, and papillate idioblasts. Its lateral leaf lamina on the lower surface covered by elongate, straight-walled, and papillate idioblasts (vs. lacking idioblasts or, if present, few and sparsely distributed on the acroscopic 1/2 and lacking on the basiscopic 1/2), and the acroscopic halves of the laminae 1/2 (vs. 1/3) wider than the basiscopic halves. Additionally, the lateral leaves of *S. guaramacalensis* along the main stems are similar in size, while those of *S. meridensis* are smaller in the proximal portion of the stems compared to the distal portion of the axis. *Selaginella guaramacalensis* is also notably different from *S. meridensis* by its broadly elliptic to broadly ovate-elliptic (vs. narrowly elliptic or broadly ovate) median leaves, with the inner margins entire along the proximal 1/3 and short-ciliate, serrate, or dentate along distal 2/3 (vs. short-ciliate throughout). Additionally, the axillary leaves on the main stems are ovate to ovate-lanceolate (vs. lanceolate to lanceolate-elliptic). Finally, *Selaginella guaramacalensis* has been collected on the northeastern Andean flank in the Guaramacal region (Portuguesa and Trujillo), whereas *S. meridensis* is known from the western-facing slopes of the same mountain system in Mérida. This pattern of occurrence on opposite slopes is consistent with an allopatric (or possibly peripatric) origin, because gene flow between humid montane populations may be reduced by intervening dry inter-Andean valleys. Such valleys within the Venezuelan Andes are reported to support xerophytic vegetation, which can function as an ecological barrier between montane wet-forest/cloud-forest habitats (Duno de Stefano et al. 2009).

### Selaginella liesneri

Taxon classificationPlantaeSelaginellalesSelaginellaceae

Valdespino sp. nov.

66222D05-310C-5CE4-8B0E-29D3329C2F3C

urn:lsid:ipni.org:names:77381373-1

**Figure 18.**
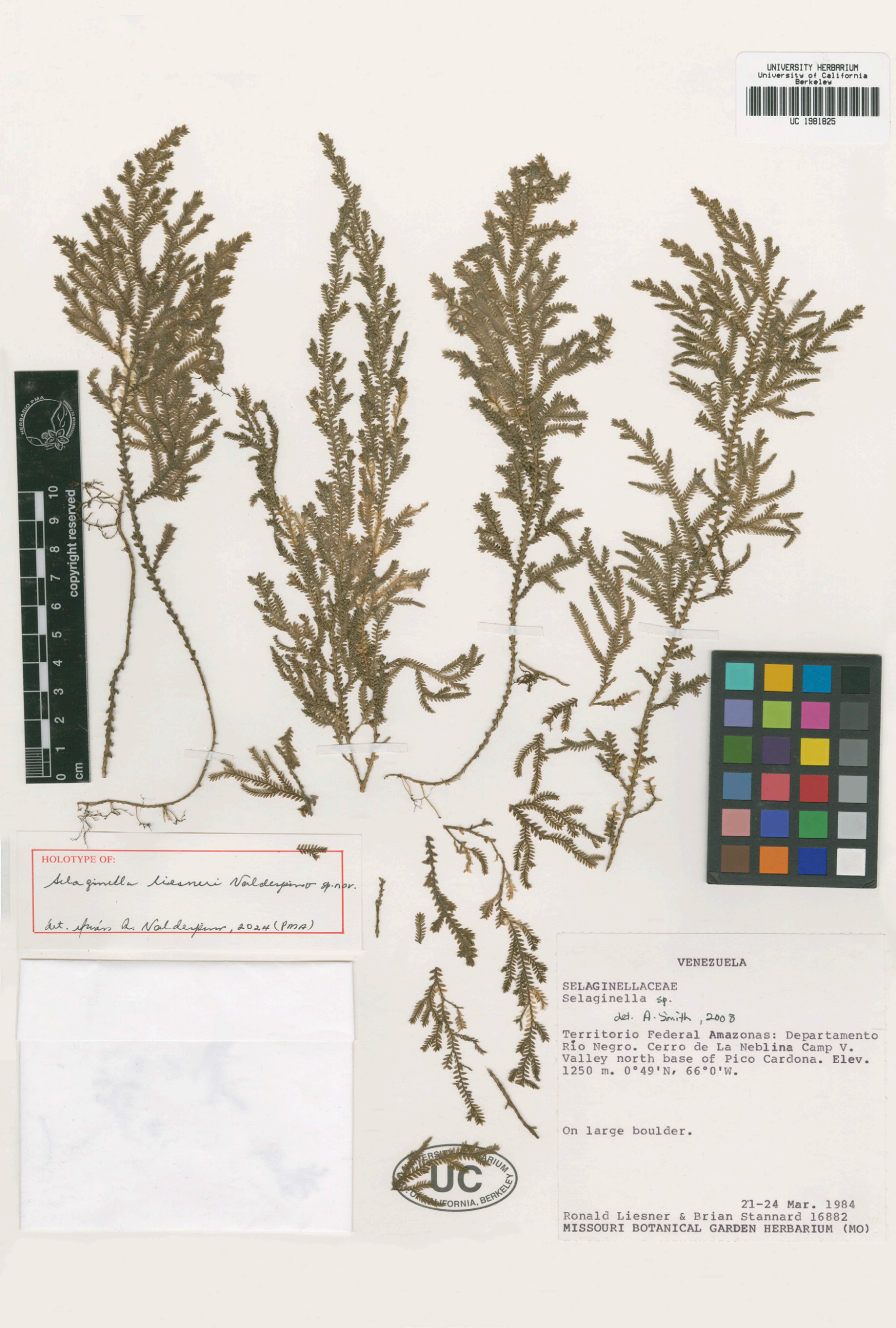
Holotype of *Selaginella liesneri* Valdespino. Digitized image courtesy of the herbarium of the University of Panama (PMA), from a loaned specimen from the herbarium of the University of California, *R.L. Liesner & B.L. Stannard 16882*, UC.

**Figure 19.**
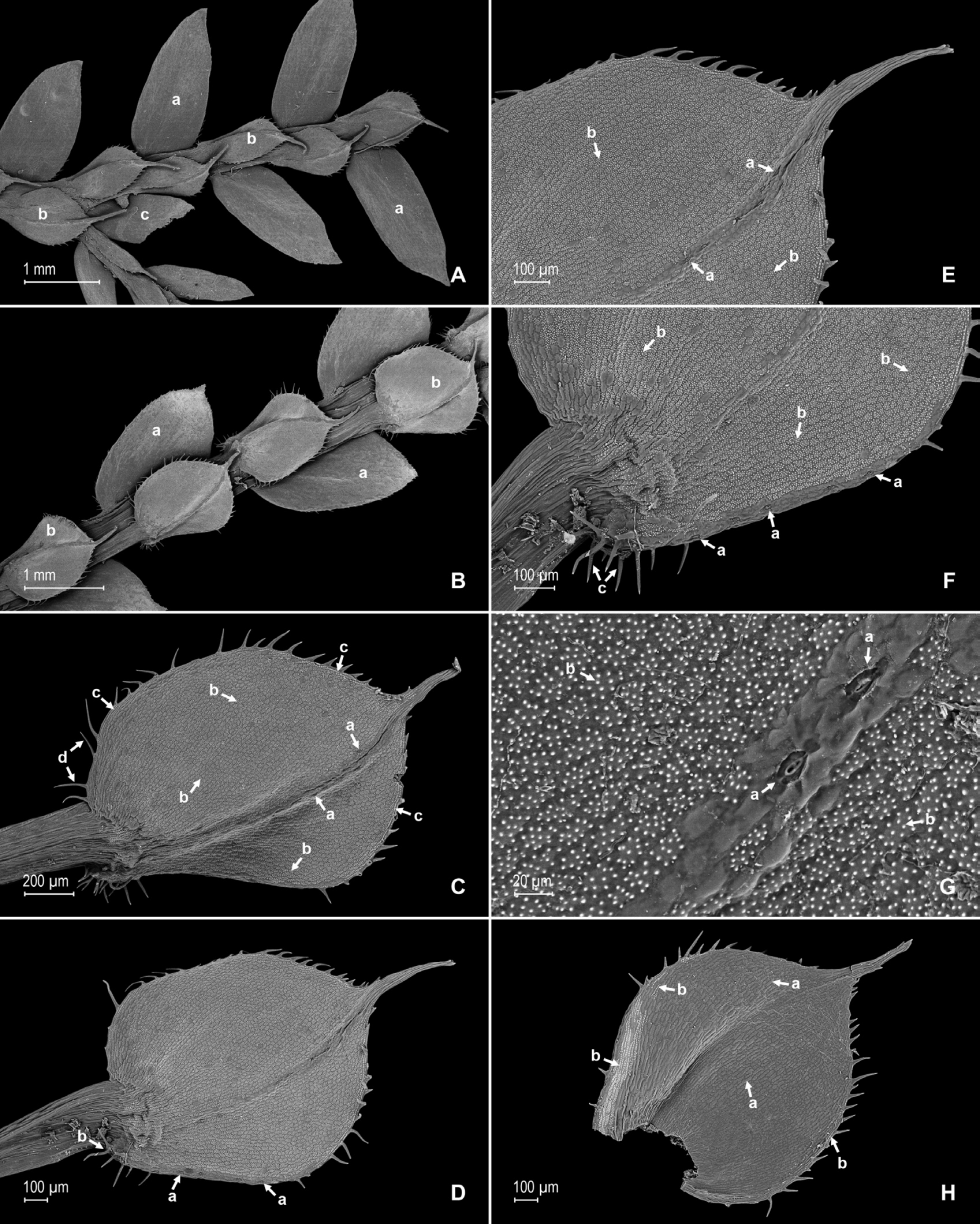
*Selaginella liesneri* Valdespino. **A**. Section of the stem, upper surface; note lateral (a), median (b), and axillary (c) leaves; **B**. Close-up of a section of the stem, upper surface; note lateral (a) and median (b) leaves; **C**. Median leaf, upper surface; note stomata along the midrib (a), quadrangular to rounded, sinuate-walled cells on leaf lamina (b), and marginal, elongate, straight-walled, papillate idioblasts (c), and long cilia along the inner margin (d); **D**. Median leaf, upper surface; note stomata along the midrib, cells on the leaf lamina, and marginal idioblasts as in **C**, marginal to submarginal stomata along the proximal, outer margin (a), and tufted hairs on the outer base (b); **E**. Close-up of the distal portion and apex of the median leaf, upper surface; note stomata along the distal portion of the midrib (a), and quadrangular and papillate cells on the leaf lamina (b); **F**. Close-up of the proximal portion and base of the median leaf, upper surface; note submarginal to marginal stomata along the proximal 1/3 of the outer margin (a), cells on the leaf lamina (b) as in **E**, and the outer base tufted with long cilia (c); **G**. Close-up of the mid-section of the median leaf, upper surface; note stomata along the midrib (a) and quadrangular and papillate cells on the leaf lamina (b) (same leaf as in **E**); **H**. Median leaf, lower surface; note quadrangular to elongate, sinuate-walled, and laevigate cells (a), and elongate, straight-walled, and papillate idioblasts along the proximal, outer, marginal and submarginal portion of the leaf lamina (b). **A–H** from the holotype, *R.L. Liesner & B.L. Stannard 16882*, UC.

**Figure 20.**
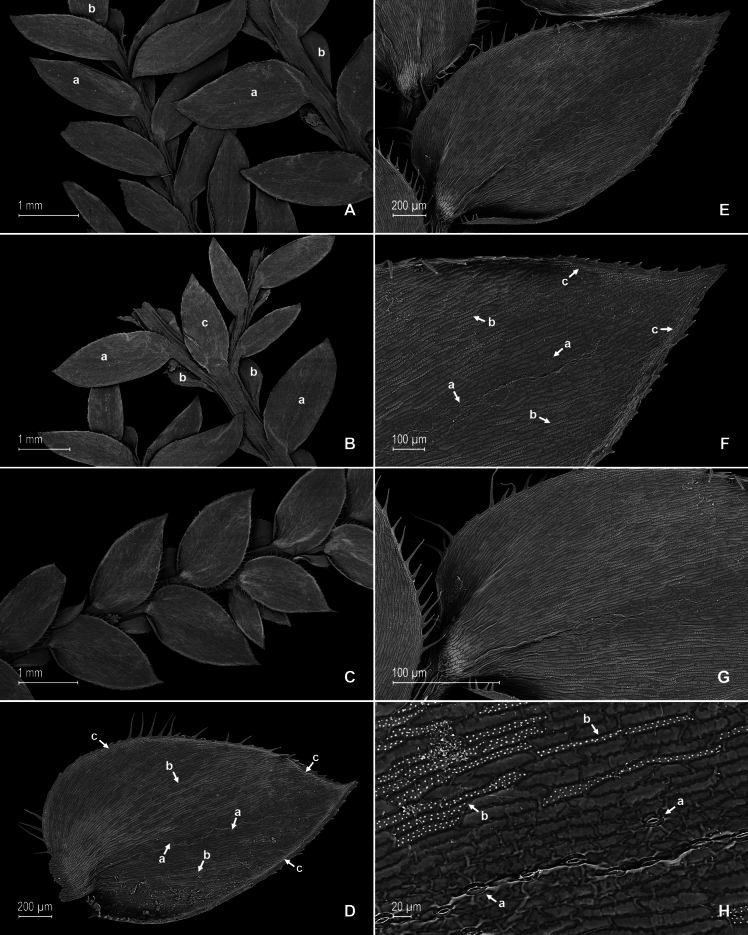
*Selaginella liesneri* Valdespino. **A**. Section of the stem, lower surface; note lateral (a) and median (b) leaves; **B**. Section of the stem, lower surface; note lateral (a), median (b), and axillary (c) leaves; **C**. Close-up of a section of the stem, lower surface; note leaves as in **A**; **D**. Lateral leaf, lower surface; note stomata along the midrib (a), elongate, straight-walled, and papillate idioblasts on the leaf lamina (b) and marginally to submarginally (c); **E**. Lateral leaf, lower surface; **F**. Close-up of the distal portion and apex of the lateral leaf, lower surface; note stomata along the midrib (a), elongate, straight-walled, and papillate idioblasts on the leaf lamina (b), and marginally to submarginally (c) (same leaf as in **E**); **G**. Close-up of proximal portion and base of the lateral leaf, lower surface; note idioblasts on the leaf lamina and margins as in **E**; **H**. Close-up of the mid-section of the lateral leaf, lower surface; note stomata along midrib (a) and elongate, straight-walled, and papillate idioblasts on the leaf lamina (b) (same leaf as in **E**). **A–H** from the holotype, *R.L. Liesner & B.L. Stannard, 16882*, UC.

**Figure 21.**
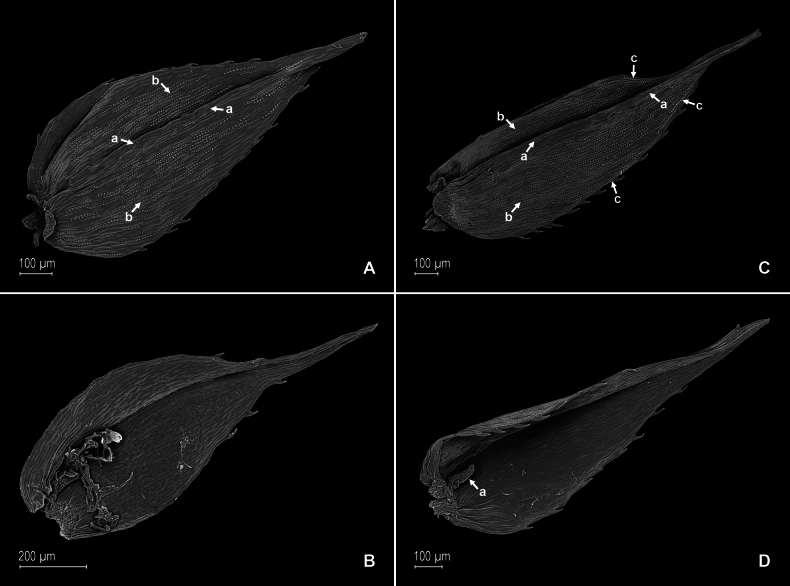
*Selaginella liesneri* Valdespino. **A**. Dorsal sporophyll, upper surface, note stomata along the midrib (a) and elongate, straight-walled, and papillate idioblasts on the leaf lamina (b); **B**. Dorsal sporophyll, lower surface; **C**. Ventral sporophyll, upper surface; note stomata along the midrib (a) and elongate, straight-walled, and papillate idioblasts on the sporophyll lamina (b) and along the margin (c); **D**. Ventral sporophyll, lower surface; note ligule (a). **A–D** from the holotype, *R.L. Liesner & B.L. Stannard, 16882*, UC.

**Figure 22.**
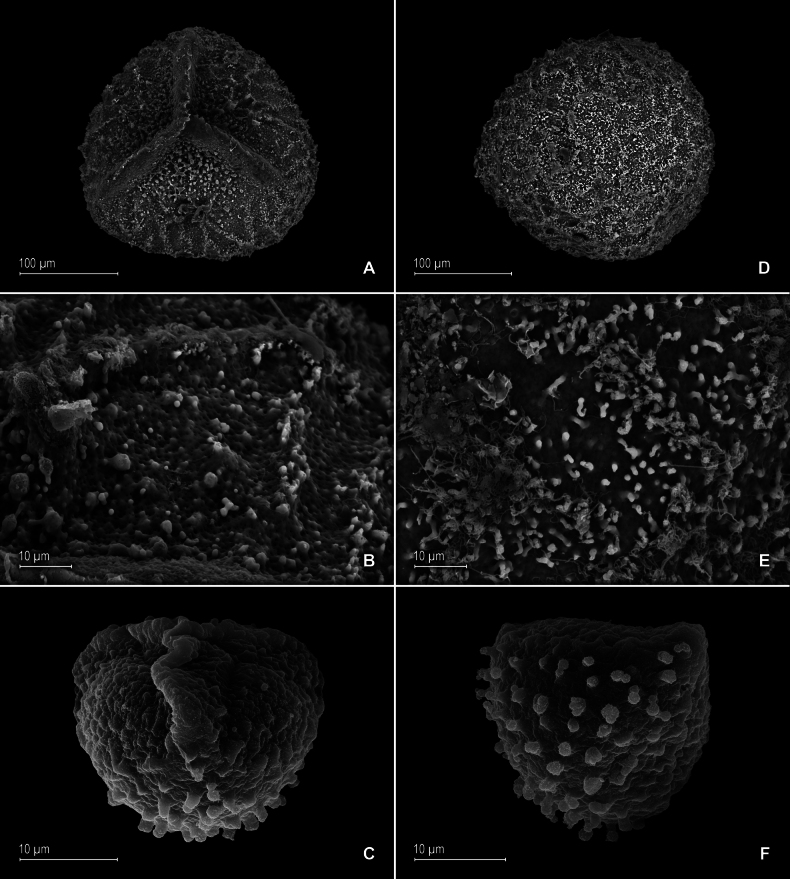
*Selaginella liesneri* Valdespino. **A**. Megaspore, proximal face; **B**. Close-up of the megaspore, proximal face (same spore as in **A**); **C**. Microspore, equatorial-proximal face; **D**. Megaspore, distal face; **E**. Close-up of the megaspore, distal face (same spore as in **D**); **F**. Microspore, distal face. **A–D** from the holotype, *R.L. Liesner & B.L. Stannard 16882*, UC.

Figs 18, 19, 20, 21, 22

#### Diagnosis.

*Selaginella liesneri* differs from *S. moritziana* Spring var. *pearcei* (Baker) Valdespino by its erect (vs. ascending to suberect) stems, leaves upper surfaces without (vs. with conspicuous, elongate idioblasts), the lateral leaves upper surfaces without (vs. with) short submarginal to marginal hairs near the basiscopic margins, the median leaves orbicular to orbicular-ovate (vs. ovate-lanceolate to broadly ovate), the apices long-aristate (vs. long-acuminate to short-aristate, or infrequently long-aristate), each arista measuring 0.3–0.6 (vs. each acumen or arista 0.1–0.36) mm long.

#### Type.

**Venezuela** • **Amazonas**: Río Negro. Cerro de La Neblina Camp V, Valley N base of Pico Cardona, 00°49'N, 66°00'W, alt. 1250 m, 21–24 Mar. 1984, *R.L. Liesner & B.L. Stannard 16882* (holotype: UC! [UC1981825]; isotypes: MO! [MO-101415443], NY!, PMA! [Herb. No. 102721], VEN! [Herb. No. 233157]).

#### Description.

***Plants*** terrestrial or epipetric. ***Stems*** erect, stramineous, 20–40 cm in height, 0.5–1.0 mm diam., non-articulate, not flagelliform or stoloniferous, 2- or 3-branched. ***Rhizophores*** latero-dorsal and axillary-dorsal, restricted to the proximal-most portion (3–7 cm) of the stems, filiform, 0.15–0.4 mm diam. ***Leaves*** below the first branches seemingly monomorphic and strongly appressed along the proximal 1/2 of the main stems, otherwise heteromorphic, chartaceous to coriaceous, both surfaces glabrous, the upper surfaces bright green to yellowish-green, the lower surfaces shiny yellowish-green (both surfaces becoming brownish after field preservation, presumably with alcohol). ***Lateral leaves*** on the main stems ascending at 45° to the stems, distant, narrowly ovate to narrowly ovate-elliptic, 2.0–3.3 × 1.0–1.5 mm; bases rounded to truncate-rounded, the acroscopic bases overlapping the stems, the basiscopic bases free from the stems; margins on the upper surfaces greenish, bordered by quadrangular to rounded cells, the acroscopic margins on the lower surfaces broadly hyaline, continuously bordered by a band 1–4 cells wide of idioblasts, the idioblasts elongate, sinuate-walled, and papillate, the papillae in one row on each cell lumen, long-ciliate along proximal 1/2–3/4, otherwise dentate to denticulate distally, the basiscopic margins on the lower surfaces continuously bordered by elongate, straight- to sinuate-walled, and laevigate cells, submarginally with similar cells as in acroscopic margins on the lower surfaces, short- to long-ciliate along proximal 1/8, entire along distal 7/8; apices acute to short-attenuate, tipped by 1–3 teeth; the upper surfaces composed of irregularly shaped, somewhat roundish, rectangular to quadrangular, sinuate-walled cells, some with many papillae on each cell lumen, without stomata, the lower surfaces composed of elongate, sinuate-walled, and laevigate cells intermixed with elongate, straight-walled, and papillate idioblasts throughout, stomata on 2–4 rows along the midrib on central 2/3 of the leaf lamina. ***Median leaves*** distant to slightly imbricate, ascending, orbicular to orbicular-ovate, 1.4–1.7 × 0.3–0.5 mm; bases subcordate with the inner bases rounded and glabrous and the outer bases decurrent, with a tufted, short- to long-ciliate knob, each with 8–12 long cilia, each measuring 100–200 µm long; the inner margins on the upper surfaces narrowly hyaline, continuously bordered by a band 1 or 2 cells wide of idioblasts, the idioblasts elongate, straight-walled, and papillate, the papillae in one row on each cell lumen, on the lower surfaces green, continuously bordered by 2–4 elongate, straight-walled, and laevigate cells intermixed with sparse elongate, straight-walled, and papillate idioblasts, entire along proximal 1/4, short- to long-ciliate along distal 3/4, the outer margins on the upper surfaces greenish along proximal 1/2, bordered by round, laevigate cells, the distal 1/2 hyaline, continuously bordered by a band 1–3 cells wide of idioblasts, the idioblasts elongate, straight-walled, and papillate, the papillae in one row on each cell lumen, on the lower surfaces hyaline along proximal 1/3–1/2, bordered by 3–6 cells wide of idioblasts, the idioblasts elongate, straight-walled, and papillate, the papillae in 1–3 rows on each cell lumen, on distal 1/2–2/3, bordered by 1–6 cells wide of elongate, straight-walled, and laevigate cells, entire along proximal 1/2, otherwise sparsely long-ciliate to serrate along distal 1/2; apices long-aristate, each arista 0.3–0.6 mm long, tipped by 1–3 teeth; both surfaces without idioblasts, the upper surfaces comprising quadrangular to rounded, sinuate-walled cells, most of these covered by 6–20 papillae, with stomata in 1 or 2 rows along distal 1/2 of the midribs and along proximal 1/2 of the outer margins, the lower surfaces comprising elongate, sinuate-walled cells almost throughout, except for the outer, submarginal to marginal 1/2 where elongate, straight-walled, and papillate idioblasts occur, each idioblast with papillae in 1–3 rows, without stomata. ***Axillary leaves*** ovate to lanceolate, 2.0–3.0 × 0.9–1.6 mm; bases rounded, glabrous; margins narrowly hyaline, continuously bordered by a band 1 or 2 cells wide of idioblasts, the idioblasts similar to those on the acroscopic margins of the lateral leaves on the lower surfaces, long-ciliate along proximal 1/2–2/3, short-ciliate to dentate distally; apices acute, tipped by 2 or 3 teeth. ***Strobili*** single and terminal on the branches, 2.0–8.0 mm long. ***Sporophylls*** monomorphic, without a laminar flap, ovate to narrowly ovate, 1.4–1.7 × 0.5–0.9 mm, each with a strongly developed and distally dentate keel along the midrib on the dorsal sporophylls and entire on the ventral sporophylls; bases rounded; margins narrowly hyaline, dentate; apices long-acuminate, each acumen 0.2–0.4 mm long with margins dentate and tipped by 1–3 teeth; ***dorsal sporophylls*** with the upper surfaces green with elongate, straight- and sinuate-walled, laevigate cells and with elongate, straight-walled, and papillate idioblasts, the lower surfaces silvery green and comprising elongate or quadrangular, sinuate-walled, and laevigate cells; ***ventral sporophylls*** with the upper surfaces mostly comprised of elongate, straight-walled, and papillate idioblasts and the lower surface comprising elongate or quadrangular, sinuate-walled, and laevigate cells. ***Megasporangia*** in two ventral rows; ***megaspores*** light yellow, rugulate, rugulate-reticulate to reticulate with open to close reticula of low ridges on the proximal faces, without a distinct equatorial flange, the microstructure scabrate and perforate, the distal faces rugulate-reticulate to reticulate with open to close reticula of low ridges, with the microstructure scabrate and perforate, 230–260 µm diam. ***Microsporangia*** in two dorsal rows; ***microspores*** yellow or light-tan colored, the proximal faces rugulate with the microstructure echinate, the distal faces capitate to baculate with these elements and the microstructure echinate over a rugulate background, 22–26.5 µm diam.

#### Habitat and distribution.

*Selaginella liesneri* is known only from the type collection from Cerro de la Neblina, Amazonas state, Venezuela. It grows in primary tropical wet, montane forests at 1250 m.

#### Eponym.

This species is named after Ronald L. Liesner (1944–present), a staff scientist at the Missouri Botanical Garden (MO), in recognition of his prolific and distinguished career as a botanist and plant collector, who, in addition, has collected several *Selaginella* novelties from Venezuela, including the one here described.

#### Conservation assessment.

*Selaginella liesneri* is known only from the type collection from Cerro de la Neblina (Amazonas, Venezuela). Based on a single georeferenced occurrence, the extent of occurrence (EOO) cannot be estimated, whereas the area of occupancy (AOO) is 4 km^2^. The species is therefore known from a single location and an Area of Occupancy (AOO) of 4 km^2^. The species is therefore known from one location. Although the type locality lies within the Serranía La Neblina protected-area complex, management limitations and illegal mining pressures have been documented in the broader region, including illegal gold mining in lowland sectors and incursions within protected areas (Huber 2001), which could result in a continuing decline in habitat quality. Accordingly, we provisionally assess *S. liesneri* as Critically Endangered (CR) under criterion B2ab(iii).

#### Discussion.

*Selaginella liesneri* is characterized by its erect stems with latero-dorsal, axillary-dorsal, and filiform rhizophores restricted to the proximal-most portion (3–7 cm) of the stems, the leaves without elongate and papillate idioblasts on the upper surfaces, the lateral leaves narrowly ovate to narrowly ovate-elliptic with the upper surfaces glabrous, the axillary leaves ovate to lanceolate with rounded, glabrous bases, narrowly hyaline and long-ciliate along the proximal 1/2–2/3 and short-ciliate to dentate distally. It is further characterized by its median leaves orbicular to orbicular-ovate with the bases plane in side view, subcordate, the inner base glabrous and rounded, the outer base decurrent and tufted with a long-ciliate knob or auricula, each with 8–12 long cilia, megaspores light yellow, and microspores yellow, or light-tan colored.

*Selaginella liesneri* is morphologically somewhat similar to *S. moritziana* var. *pearcei*, but the characters contrasted in the diagnosis serve to separate them. In addition, *S. liesneri* has narrowly ovate to narrowly ovate-elliptic (vs. ovate to ovate-oblong) lateral leaves, with the acroscopic margins long-ciliate along proximal 1/2–3/4, otherwise dentate to denticulate distally (vs. serrate or infrequently ciliate-denticulate along proximal 1/3, otherwise serrulate along distal 2/3), without (vs. frequently with) marginal stomata on the leaf upper surfaces, and the basiscopic margins long-ciliate along proximal 1/8 and entire along distal 7/8 (vs. entire throughout or serrulate on distal-most portion). *Selaginella liesneri* is additionally distinct from *S. moritziana* var. *pearcei* by its megaspores rugulate, rugulate-reticulate to reticulate on proximal and distal faces (vs. proximal faces reticulate-striate or corrugate-reticulate and distal faces reticulate or reticulate-corrugate), without (vs. frequently with a prominent) equatorial flange. Finally, *S. liesneri* is known to date only from the Cerro de la Neblina area in Amazonas State, within the Guayana Highlands of Venezuela, whereas *S. moritziana* var. *pearcei* has been documented only from Mérida and Trujillo, restricted to the Cordillera de Mérida, a northeastern offshoot of the Venezuelan Andes.

*Selaginella liesneri* is also morphologically similar to *S. substipitata* in overall plant shape and by having yellow megaspores, but mainly differs in its orbicular to orbicular-ovate (vs. elliptic to ovate-elliptic) median leaves with the inner margins on upper surface narrowly (vs. widely hyaline), bordered continuously by a band 1 or 2 (vs. 4–6) cells wide of idioblasts, entire along proximal 1/4, short- to long-ciliate along distal 3/4, (vs. serrate throughout on both margins), with the outer margins greenish along proximal 1/2 and narrowly hyaline along distal 1/2 (vs. broadly hyaline throughout and similar to the inner margins). It further differs in its narrowly ovate to narrowly ovate-elliptic (vs. ovate to ovate-oblong) lateral leaves, which lack (vs. have) distinct, elongate, straight-walled idioblasts on the lower surfaces of the leaf lamina. Additionally, its axillary leaf margins are long-ciliate along the proximal 1/2–2/3, and short-ciliate to dentate distally (vs. serrate to denticulate throughout), and its rugulate-reticulate to reticulate (vs. reticulate) megaspores on the distal faces.

### Selaginella mawarinumensis

Taxon classificationPlantaeSelaginellalesSelaginellaceae

Valdespino & C.López sp. nov.

ACCB3800-C751-581B-B6A2-E6D42667E5CC

urn:lsid:ipni.org:names:77381375-1

Figs 23, 24, 25, 26

#### Diagnosis.

*Selaginella mawarinumensis* is distinguished from *S. applanata* A.Braun by its elliptic to elliptic-ovate (vs. broadly-ovate, broadly ovate-elliptic to broadly elliptic) median leaves with the margins broadly (vs. narrowly) hyaline, continuously bordered by a band 3–8 (vs. 1–3) cells wide of elongate, straight-walled, and papillate idioblasts, with the outer margins sparsely, long-ciliate throughout (vs. sparsely long-ciliate along proximal 1/4–1/2, otherwise dentate to denticulate distally).

**Figure 23.**
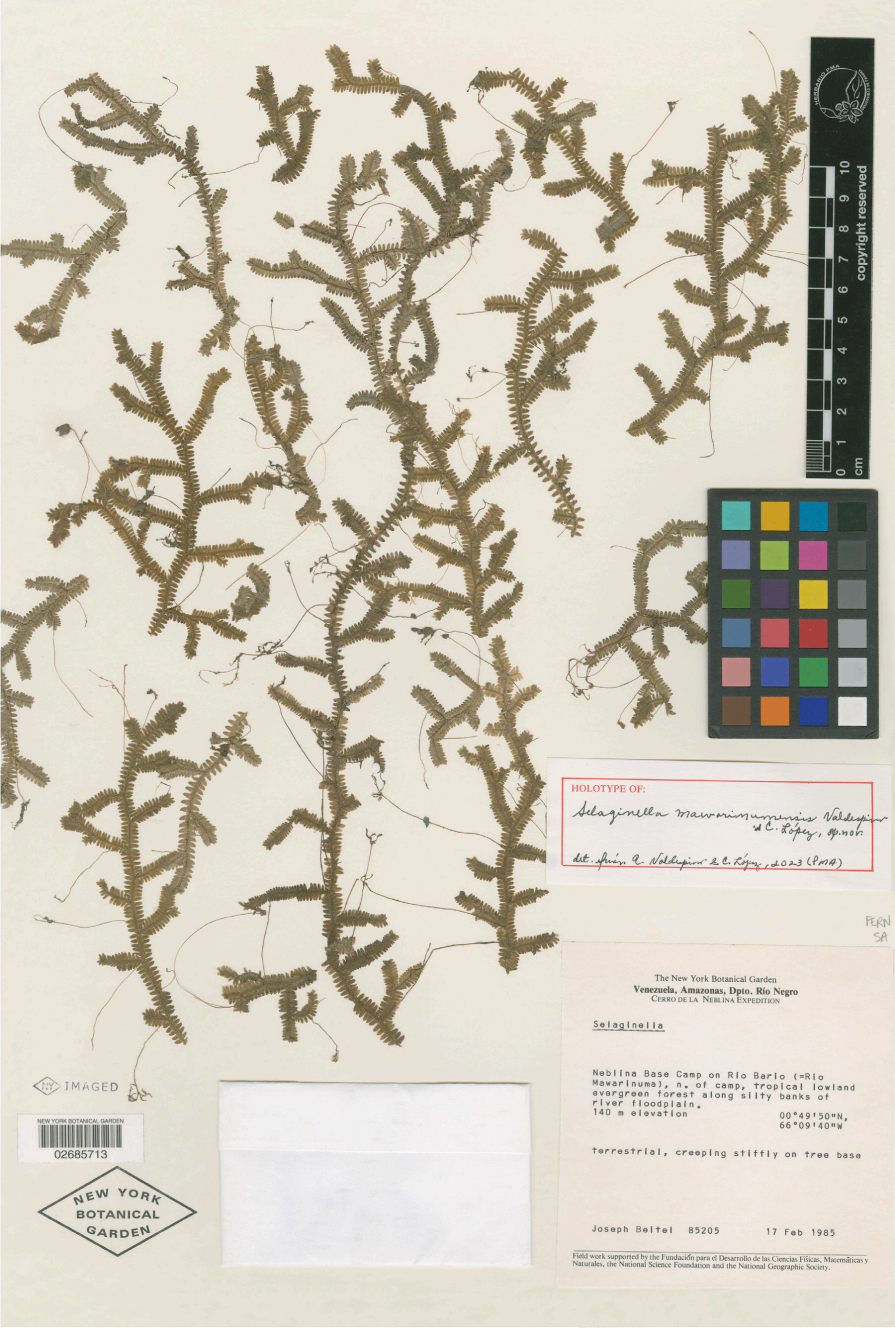
Holotype of *Selaginella mawarinumensis* Valdespino & C.López. Digitized image courtesy of the herbarium of the University of Panama (PMA), from a loaned specimen from the herbarium of the New York Botanical Garden, *J.M. Beitel 85205*, NY.

**Figure 24.**
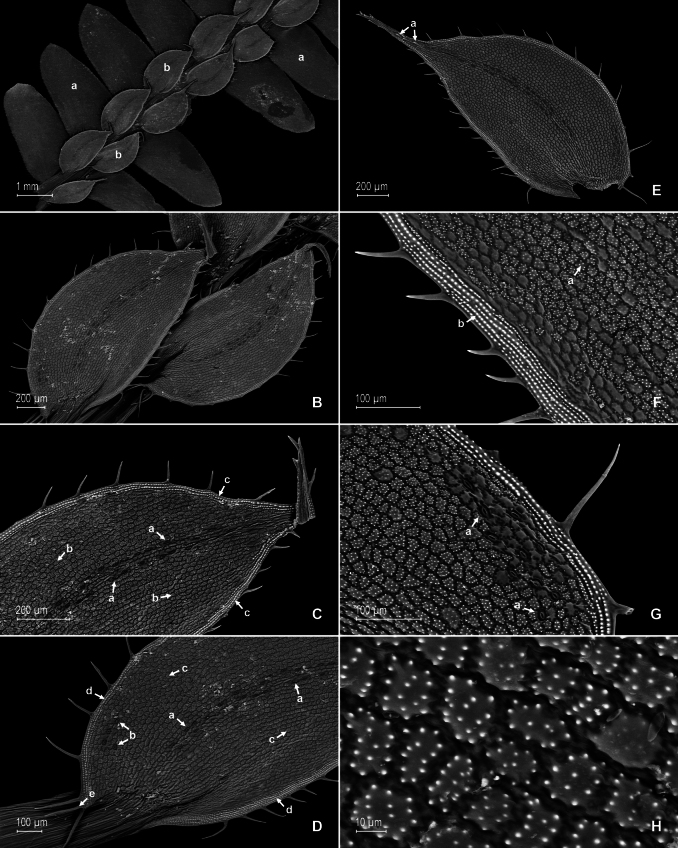
*Selaginella mawarinumensis* Valdespino & C.López. **A**. Section of the stem, upper surface; note lateral (a) and median (b) leaves; **B**. Median leaves, upper surface; **C**. Close-up of the distal portion and apex of the median leaf, upper surface (same leaf as in **B**); note stomata along the midrib (a), rounded, quadrangular to rectangular, sinuate-walled, and papillate cells on the leaf lamina (b), and elongate, straight-walled, and papillate idioblasts along the margins (c); **D**. Close-up of the proximal portion and base of the median leaf, upper surface (same leaf as in **B**); note stomata along the midrib (a) and submarginally on the proximal portion of the outer leaf half (b), rounded, quadrangular to rectangular, and papillate cells on the leaf lamina (c), marginal, elongate, straight-walled, and papillate idioblasts (d), and tufted hairs on the outer base (e); **E**. Median leaf, upper surface; note tooth-like hairs (a) along the proximal portion of the arista; **F**. Close-up of the inner half of the median leaf, upper surface (same leaf as in **E**); note rounded, quadrangular to rectangular, and papillate cells on the leaf lamina (a), and marginal, elongate, straight-walled, and papillate idioblasts (b); **G**. Close-up of the outer half of the median leaf, upper surface (same leaf as in **E**); note submarginal stomata on the proximal portion of the outer leaf half (a), and cells on the leaf lamina and marginal idioblasts as in **F**; **H**. Close-up of median leaf, upper surface; note rounded, quadrangular to rectangular, papillate cells on the leaf lamina. **A–H** from the holotype, *J.M. Beitel 85205*, NY.

**Figure 25.**
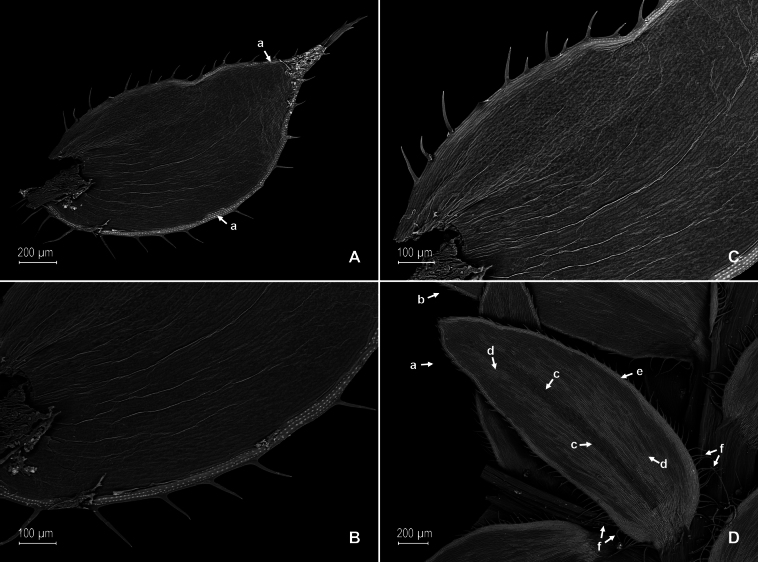
*Selaginella mawarinumensis* Valdespino & C.López. **A**. Median leaf, lower surface; note marginal, elongate, straight-walled, and papillate idioblasts (a); **B**. Close-up of the middle portion of the outer half of the median leaf, lower surface (same leaf as in **A**); note outer margin as in **A**; **C**. Close-up of the mid-section of the median leaf, lower surface (same leaf as in **A**); note distal 1/2 of inner margin with idioblasts as in **A**; **D**. Axillary leaf (a) and portions of the lateral leaves (b), lower surface; note the axillary leaf with stomata along the midrib (c), elongate, straight-walled, and papillate idioblasts on the leaf lamina (d) and similar idioblasts on the margins (e), and long-ciliate margins along the proximal half of the leaf lamina (f). **A–D** from the holotype, *J.M. Beitel 85205*, NY.

**Figure 26.**
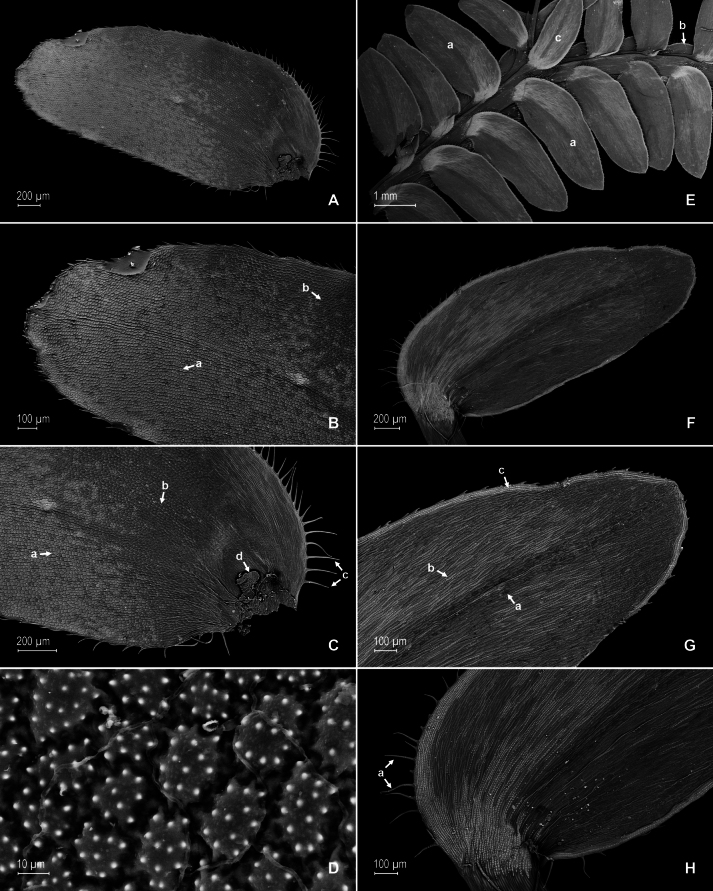
*Selaginella mawarinumensis* Valdespino & C.López. **A**. Lateral leaf, upper surface; **B**. Close-up of the distal portion and apex of the lateral leaf, upper surface (same leaf as in **A**); note rounded, quadrangular to rectangular, and papillate (a) and laevigate (b) cells; **C**. Close-up of the proximal portion and base of the lateral leaf, upper surface (same leaf as in **A**); note rounded, quadrangular to rectangular, and papillate (a) and laevigate (b) cells, long cilia along the acroscopic margin (c), and the ligule (d); **D**. Close-up of the lateral leaf, upper surface (same leaf as in **A**); note rounded, quadrangular to rectangular, sinuate-walled, and papillate cells; **E**. Section of the stem, lower surface; note lateral (a), median (b), and axillary (c) leaves; **F**. Lateral leaf, lower surface; **G**. Close-up of distal portion and apex of the lateral leaf, lower surface (same leaf as in **F**); note stomata along the midrib (a), elongate, straight-walled, and papillate idioblasts on the leaf lamina (b), and similar idioblasts on the margins (c); **H**. Close-up of the proximal portion and base of the lateral leaf, lower surface (same leaf as in **F**); note stomata, cells on the leaf lamina, and margins as in **G**, and long-ciliate acroscopic margins along the proximal portion of the lamina (a). **A–H** from the holotype, *J.M. Beitel 85205*, NY.

#### Type.

**Venezuela** • **Amazonas**: Depto. Río Negro, N of [Neblina Base] Camp, 00°49'50"N, 66°09'40"W, alt. 140 m, 17 Feb. 1985, *J.M. Beitel 85205* (holotype: NY! [NY02685713]; isotypes: PMA!, UC! [UC1551681], VEN [Herb. No. 318841]).

#### Description.

***Plants*** terrestrial, epipetric, or epiphytic on the lower portions of the tree trunks. ***Stems*** creeping, stramineous, 10–30 cm long, 0.3–0.6 mm diam., non-articulate, not flagelliform or stoloniferous, 1–3-branched. ***Rhizophores*** axillary, borne throughout the length of the stems, filiform, 0.1–0.3 mm diam. ***Leaves*** heteromorphic, chartaceous, both surfaces laevigate, the upper surfaces bright green to yellowish-green, smooth, the lower surfaces shiny green to yellowish-green (both surfaces also brownish after treatment in the field, probably with alcohol). ***Lateral leaves*** on main stems perpendicular at 90° to the stems, distant, glabrous, oblong to oblong-ovate, 2.4–3.2 × 0.8–1.8 mm; bases rounded, rounded-geniculate to rounded-truncate, the acroscopic bases strongly overlapping the stem, the basiscopic bases free from the stems; the acroscopic margins on the upper surfaces greenish, continuously bordered by a band 2–8 cells wide, the cells elongate, laevigate, and sinuate-walled, intermixed with greenish-hyaline portions, these composed of a band 1 or 2 cells wide of idioblasts, the idioblasts elongate, straight-walled and papillate, the papillae in one row on each cell lumen, on the lower surfaces broadly hyaline, continuously bordered by a band 3–20 cells wide of idioblasts, the idioblasts elongate, straight-walled, and papillate, the papillae mostly in one row or occasionally in 2 rows on each cell lumen, long-ciliate along proximal 1/4–1/3, short-ciliate along central 1/4–1/3, and dentate to denticulate along distal 1/3–1/2, the basiscopic margins on the upper surfaces, green, bordered by rounded to quadrangular, sinuate-walled, and papillate cells, the papillae abundant, and evenly distributed on each cell lumen, on the lower surfaces continuously bordered by a band 1–3 cells wide of idioblasts, the idioblasts as those in the acroscopic margins on the lower surface, long- to short-ciliate along proximal 1/4–1/3, otherwise short-ciliate to dentate distally; apices broadly acute, tipped by 3–6 teeth; upper surfaces composed of irregularly shaped, somewhat roundish, quadrangular to rectangular, sinuate-walled, and papillate cells, each cell lumen with 8–20 papillae distributed throughout, without stomata, the lower surfaces composed of elongate, sinuate-walled, and laevigate cells intermixed, particularly on acroscopic half of the leaf lamina, with elongate, straight-walled, and papillate idioblasts, stomata on 1–3 rows throughout the midrib of the leaf lamina. ***Median leaves*** imbricate, ascending, elliptic to elliptic-ovate, 1.2–1.7 × 0.6–1.2 mm; bases plane in side view, rounded to rounded-oblique with the inner bases rounded-oblique and glabrous and the outer bases, rounded and tufted with 3–5 long cilia, each 150–230 µm long; the margins on the upper surfaces broadly hyaline, continuously bordered by a band 3–8 cells wide of idioblasts, the idioblasts elongate, straight-walled, and papillate, the papillae usually in one row or occasionally in two rows on each cell lumen, the inner margins sparsely, long-ciliate along proximal 1/2–2/3, otherwise short-ciliate along distal 1/2–1/3, the outer margins sparsely, long-ciliate throughout, the inner margins on the lower surfaces with a hyaline border along distal 1/2 as on the upper surfaces, otherwise composed of a 5–7 cells wide band of elongate, sinuate-walled, and laevigate cells, the outer margins on the lower surfaces as on the upper surfaces; apices long-aristate, each arista 0.3–0.6 mm long, with tooth-like hairs on the upper surface and distally tipped by 1–3 teeth; upper and lower leaf surfaces without idioblasts, the upper surfaces mostly comprised of roundish, quadrangular to rectangular, sinuate-walled, and papillate cells, the papillae on each cell lumen as those on upper surface of lateral leaves or few, sparsely distributed, roundish, quadrangular to rectangular, sinuate-walled, and laevigate cells, with stomata in 1–3 rows the distal 2/3–3/4 of the midribs and few on proximal 1/4 of submarginal region of the outer leaf half, the lower surfaces comprising elongate, sinuate-walled, and laevigate cells, without stomata. ***Axillary leaves*** oblong to oblong-ovate or lanceolate, 1.5–2.4 × 0.7–1.0 mm; bases rounded or subcordate, glabrous; margins narrowly- to broadly hyaline, continuously bordered by a band 2–10 cells wide of idioblasts, the idioblasts similar to those on the acroscopic margins of lateral leaves, long-ciliate along proximal 1/2–2/3, short-ciliate to serrate distally; apices acute to broadly acute, tipped by 2 or 3 teeth; the upper and lower surfaces as in the lateral leaves. ***Strobili*** terminal, solitary or bifurcate on branches, 0.4–15 cm long. ***Sporophylls*** monomorphic, without a laminar flap, each with a strongly developed and dentate to denticulate keel along distal 3/4 of the lamina midrib; bases rounded; margins broadly hyaline on both surfaces, bordered by a band 2–6 cells wide of idioblasts, the idioblasts similar to those on the acroscopic margins of the median leaves, entire along proximal 2/3, otherwise sparsely denticulate on distal 1/3 on the dorsal sporophylls and on the ventral sporophylls entire; apices short-acuminate, each acumen 0.1 or 0.2 mm long, with the margins sparsely denticulate and tipped by 1–3 teeth; ***dorsal sporophylls*** ovate, 1.0–1.2 × 0.5–0.7 mm; the upper surfaces green and cells as in the median leaves, the lower surfaces silvery green and comprising elongate, sinuate-walled cells; ***ventral sporophylls*** lanceolate to ovate-lanceolate, 1.1–1.4 × 0.4–0.6 mm; bases rounded; the upper and lower surfaces silvery green and comprising elongate, sinuate-walled cells. ***Megasporangia*** on two ventral rows; ***megaspores*** underdeveloped, cream to light-yellow, proximal faces rugulate without a prominent equatorial flange, the microstructure not observed, the distal faces reticulate, with open or closed reticulae, the microstructure not studied, 150–200 μm diam. ***Microsporangia*** in two dorsal rows; ***microspores*** underdeveloped, yellow to light-orange, the sculpturing pattern and diameter unknown.

#### Habitat and distribution.

*Selaginella mawarinumensis* grows as a terrestrial, creeping plant in low-elevation riparian habitats (140–170 m) in the Cerro de la Neblina, Amazonas State, Venezuela. It occurs along the Río Mawarinuma, near the Neblina Base Camp and at the Puerto Chimo camp, typically on moist riverbanks, steep banks, and riverbeds, and occasionally at the bases or lower trunks of trees.

#### Etymology.

The species is named after the Mawarinuma River in Venezuela, in the area where most specimens were collected.

#### Conservation assessment.

*Selaginella mawarinumensis* is known from the lower Río Mawarinuma area near the Cerro de la Neblina Base Camp and nearby Puerto Chimo camp, in Amazonas, Venezuela. Although nine collections are known, they correspond to only three distinct georeferenced localities, all clustered within a very small area. Using GeoCAT, the Extent of Occurrence (EOO) is 0.860 km^2^ and the Area of Occupancy (AOO) is 8 km^2^. Given the very restricted range and the fact that the records likely represent a single location (*sensu* IUCN), any localized disturbance could rapidly affect the entire known population. In addition, human pressures in the Neblina protected-area complex, including illegal mining and limitations in effective management, have been documented and may contribute to a continuing decline in habitat quality (Huber 2001). We therefore provisionally assess *S. mawarinumensis* as Critically Endangered (CR) under criterion B1ab(iii)+B2ab(iii).

#### Additional specimens examined (paratypes).

**Venezuela** • **Amazonas**: Depto. Río Negro, near Cerro de la Neblina Base Camp on Río Mawarinuma, 00°50'N, 66°10'W, alt. 140 m, 25–26 Nov. 1984, *R.L. Liesner 17284* (MO, NY, UC, VEN), • Neblina Base Camp on Río Bario (= Río Mawarinuma), SE of [Neblina Base] Camp, at end of Bongo trail, 00°49'50"N, 66°09'40"W, alt. 140 m, 26 Jan. 1985, *J.M. Beitel 85042* (NY, VEN), • N of [Neblina Base] Camp, 00°49'50"N, 66°09'40"W, alt. 140 m, 21 Feb. 1985, *J.M. Beitel 85240* (MO, NY-2 sheets, UC, W-n.v.), 23 Feb. 1985, *J.M. Beitel 85261* (NY, UC, VEN), • NE tip of large island upstream from Neblina Base Camp along Río Mawarinuma, 00°50'N, 66°10'W, alt. 160 m, 27 Nov. 1984, *R. Kral & R.L. Liesner 71839* (UC, VEN), • near Cerro de la Neblina Base Camp on Río Mawarinuma, 00°50'N, 66°10'W, alt. 140 m, 5 Feb. 1984, *R.L. Liesner 15673* (MO-2 sheets, NY, UC, VEN, WIS-digital image), 25–26 Nov. 1984, *R.L. Liesner 17301* (F, MO, UC), • Puerto Chimo camp, 00°50'N, 66°07'W, alt. 170 m, 23 Apr. 1984, *W.W. Thomas 3222* (NY, PMA, VEN).

#### Discussion.

*Selaginella mawarinumensis* is characterized by its long-creeping stems, filiform rhizophores throughout the stems, and elliptic to elliptic-ovate median leaves with the inner and outer margins distinctly hyaline, bordered by 3–6 rows of elongate, straight-walled idioblasts, each idioblast with papillae arranged in a single row within the cell lumen, the inner margins sparsely long- to short-ciliate and the outer margins long-ciliate throughout, the midribs with stomata along the distal 3/4 and a few stomata (2–4) on the proximal submarginal portion of the outer leaf halves, and the outer bases tufted with 3 long cilia. Moreover, the upper surfaces of the median leaves are composed of rounded to quadrangular, papillate cells, each with 8–18 papillae in the cell lumen. In addition, its lateral leaves are narrowly oblong to oblong with the upper surface similar to the median leaves, the acroscopic margins long-ciliate along proximal 1/4–1/3, short-ciliate along the central 1/4–1/3, and dentate to denticulate along distal 1/3–2/4, the basiscopic margins long- to short-ciliate along proximal 1/4–1/3, otherwise short-ciliate to dentate distally, on the lower leaf surfaces, the acroscopic margins hyaline throughout and bordered by a band 3–17 cells wide of elongate, straight-walled, and papillate idioblasts, with papillae arranged in a single row, while the basiscopic margins are also hyaline but bordered by a band 2 cells wide of similar idioblasts.

*Selaginella mawarinumensis* shares a similar growth habit and vegetative leaf morphology with *S. applanata*, leading to confusion between the two. However, a detailed examination shows that these two taxa are distinct species. *Selaginella mawarinumensis* can be easily distinguished from *S. applanata* by the morphological features outlined in the diagnosis. They differ further in that *S. mawarinumensis* has median leaves with stomata along the distal 3/4 (vs. throughout) of the midribs, and lateral leaves that are oblong to oblong-ovate (vs. broadly ovate or broadly oblong), with the upper surfaces laevigate (vs. verrucose), with the lower surfaces of the leaf laminae having the acroscopic margins hyaline throughout (vs. along distal 2/3), bordered by a band 3–17 (vs. on the hyaline portion bordered only by 1 or 2) cells wide of elongate, straight-walled, and papillate idioblasts. Additionally, its lateral leaf basiscopic margins are also hyaline but bordered by a band 2 cells wide of similar idioblasts (vs. greenish and lacking idioblasts), with broadly acute (vs. obtuse to broadly oblique-obtuse) apices.

### Selaginella monoloba

Taxon classificationPlantaeSelaginellalesSelaginellaceae

A.R.Sm. ex C.López, Valdespino & Mostacero sp. nov.

39C4EC0F-644F-52AB-8E64-8CD97979FEDB

urn:lsid:ipni.org:names:77381377-1

**Figure 27.**
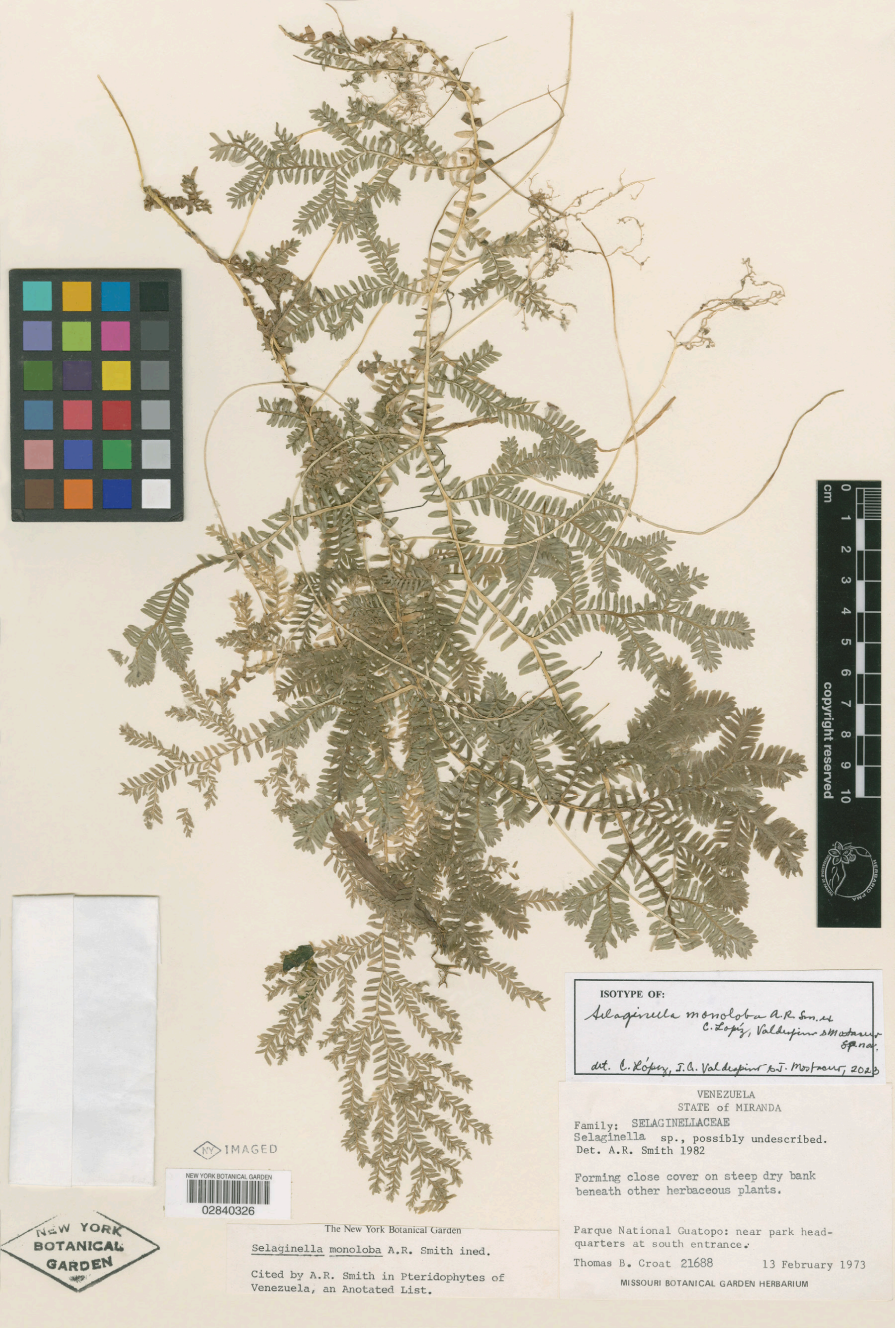
Isotype of *Selaginella monoloba* A.R.Sm. ex C.López, Valdespino & Mostacero. Digitized image, courtesy of the herbarium of the University of Panama, Panama (PMA), from a loaned specimen from the herbarium of the New York Botanical Garden, *T.B. Croat 21688*, NY.

**Figure 28.**
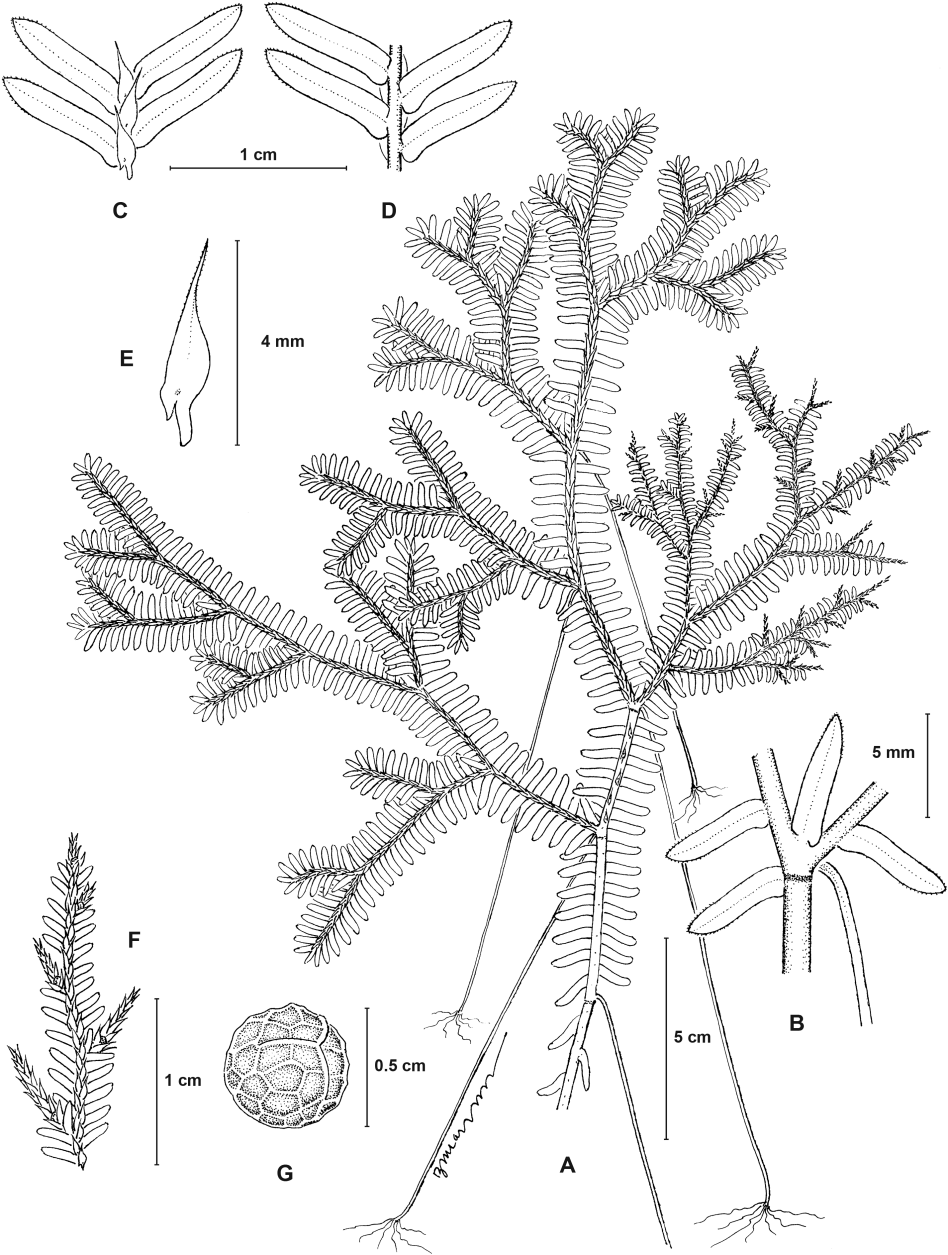
Line drawing of an isotype of *Selaginella monoloba* A.R.Sm. ex C.López, Valdespino & Mostacero. **A**. Habit; **B**. Section of the stem, lower surface; showing lateral and axillary leaves and stem articulation; **C**. Section of the stem, upper surface; **D**. Section of the stem, lower surface; **E**. Median leaf, general view; **F**. Terminal branch with strobili; **G**. Megaspore, distal face. Drawing by the late Bruno Manara and digitized by Julian Mostacero G., both at VEN; *T.B. Croat 21688*, VEN.

**Figure 29.**
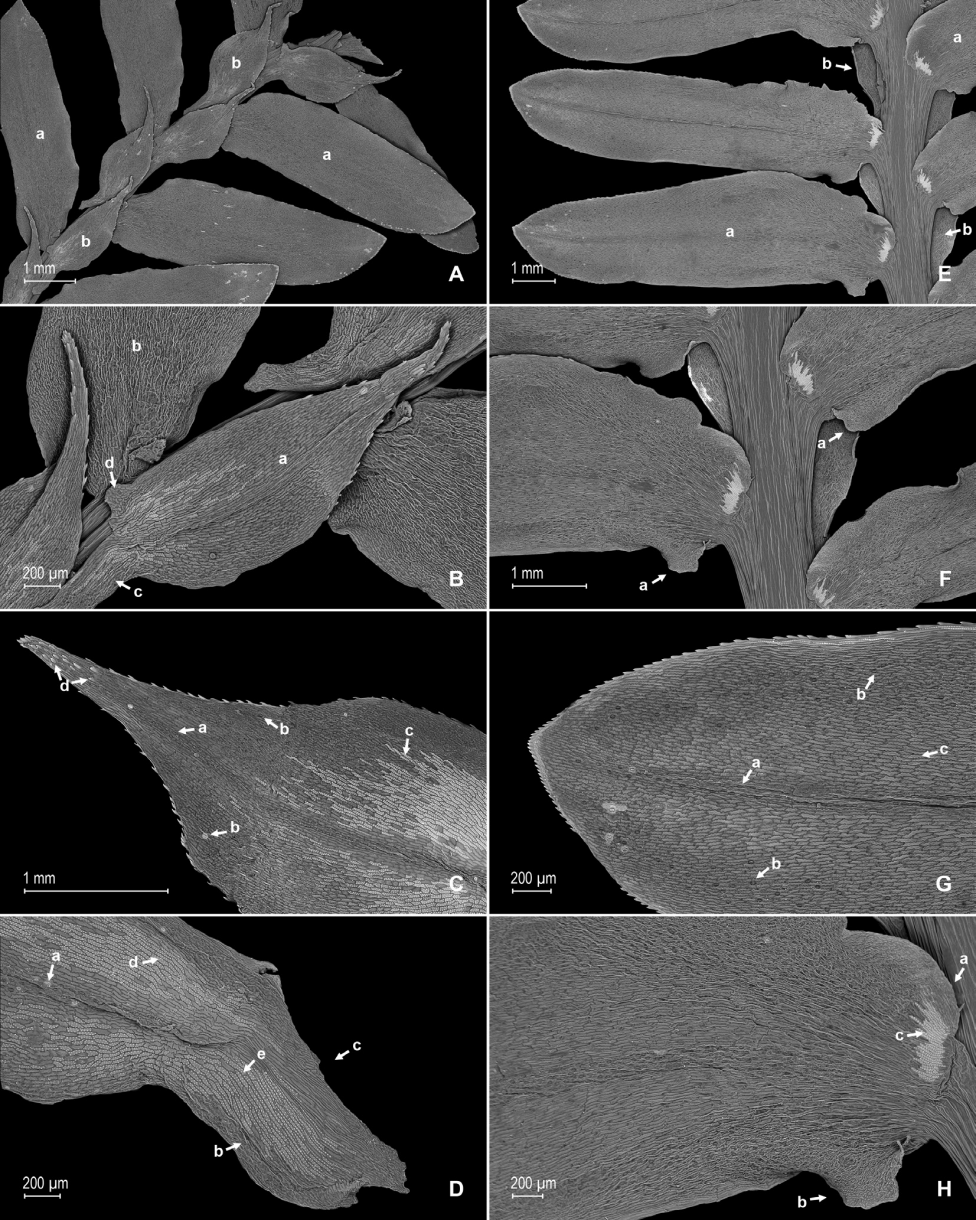
*Selaginella monoloba* A.R.Sm. ex C.López, Valdespino & Mostacero. **A**. Section of the stem, upper surface; note lateral (a) and median (b) leaves; **B**. Median leaves (a) and lateral leaves (b), upper surface; note the median leaf large outer auricle (c) and reduced inner auricle (d); **C**. Close-up of the distal portion and apex of the median leaf (same leaf as in **B**), upper surface; note stomata along the midrib (a) and submarginally on the leaf lamina (b), elongate, straight-walled, and papillate idioblasts (c) and tooth-like hairs on the arista (d); **D**. Close-up of the proximal portion and base of the median leaf, upper surface (same leaf as in **B**); note stomata along the midrib (a) and submarginally (b) on the large, outer auricle (c), and elongate, straight-walled, and papillate idioblasts on the leaf lamina (d) and auricle (e); **E**. Section of the stem, lower surface; note lateral (a) and median (b) leaves; **F**. Close-up of a stem section showing the mid to proximal portion and bases of lateral leaves (same leaves as in **E**), lower surface; note geniculate to auriculate basiscopic bases (a) on lateral leaves; **G**. Close-up of the distal and apical portion of the lateral leaf, lower surface (same leaf as in **E**); note stomata along the midrib (a) and submedially to submarginally (b) on the leaf lamina, and elongate, sinuate-walled, and laevigate cells (c); **H**. Close-up of the proximal portion and base of the lateral leaf, lower surface (same leaf as in **E**); note stomata as in **G**, rounded acroscopic base (a) and geniculate to auriculate basiscopic base (b), and elongate, straight-walled, and papillate idioblasts on the proximal-median portion of the acroscopic base (c). **A–H** from the isotype, *T.B. Croat 21688*, NY.

**Figure 30.**
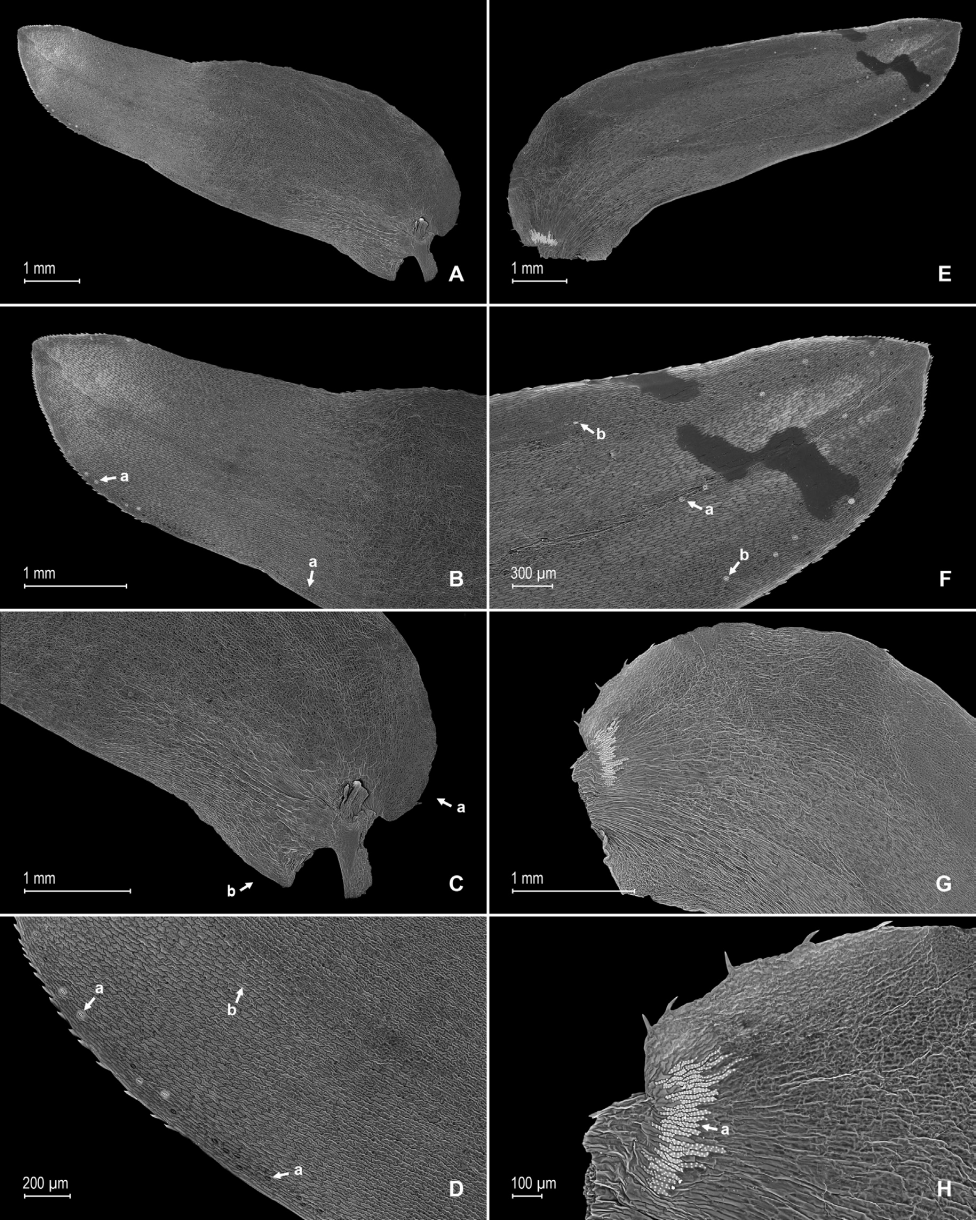
*Selaginella monoloba* A.R.Sm. ex C.López, Valdespino & Mostacero. **A**. Lateral leaf, upper surface; **B**. Close-up of the distal portion and apex of the lateral leaf, upper surface (same leaf as in **A**); note basiscopic, submarginal stomata (a); **C**. Close-up of the proximal portion and base of the lateral leaf, upper surface (same leaf as in **A**); note the rounded, acroscopic base (a) and geniculate to auriculate basiscopic base (b); **D**. Close-up of the basiscopic half of the lateral leaf, upper surface (same leaf as in **A**); note submarginal stomata (a) and rectangular to elongate, sinuate-walled laevigate cells on the leaf lamina (b); **E**. Lateral leaf, lower surface; **F**. Close-up of the distal portion and apex of the lateral leaf, lower surface (same leaf as in **E**); note stomata along the midrib (a) and throughout the leaf lamina (b); **G**. Close-up of the proximal portion and base of the lateral leaf, lower surface (same leaf as in **E**); **H**. Close-up of the proximal portion and base of the lateral leaf, lower surface (same leaf as in **E** and **G**); note elongate, straight-walled, and papillate idioblasts on the proximal-median portion of the acroscopic base (a). **A–H** from the isotype, *T.B. Croat 21688*, NY.

**Figure 31.**
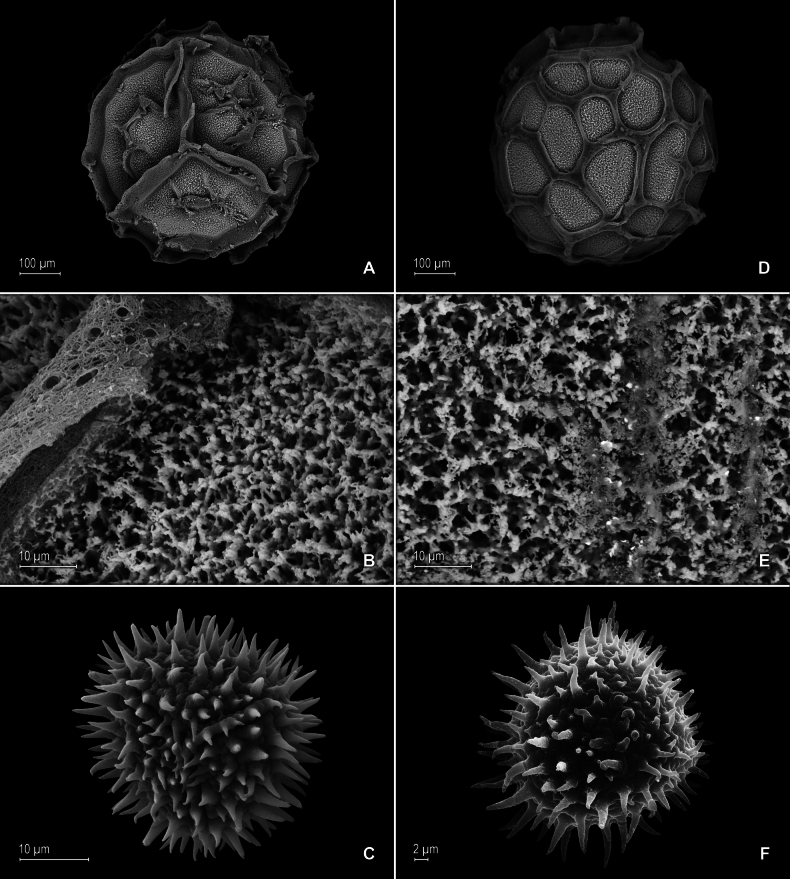
*Selaginella monoloba* A.R.Sm. ex C.López, Valdespino & Mostacero. **A**. Megaspore, proximal face; **B**. Close-up of the megaspore, proximal face (same spore as in **A**); **C**. Microspore, proximal face; **D**. Megaspore, distal face; **E**. Close-up of the megaspore, distal face (same spore as in **D**); **F**. Microspore, distal face. **A–F** from the isotype, *T.B. Croat 21688*, NY.

Figs 27, 28, 29, 30, 31

#### Diagnosis.

*Selaginella monoloba* differs from *S. kunzeana* A.Braun by its peltate (vs. basifixed) median leaves, with two divergent (vs. parallel) auricles, axillary leaves biauriculate (vs. exauriculate), and greenish (vs. strongly hyaline) leaf margins.

#### Type.

**Venezuela** • **Miranda**: Parque Nacional Guatopo: near park headquarters at south entrance, 13 Feb. 1973, *T.B. Croat 21688* (holotype: UC! [UC1481428]; isotypes: MEXU!, MO! [MO-1808922], NY! [02840326], fragment PMA!, VEN! [Herb. No. 347379].

#### Description.

***Plants*** terrestrial. ***Stems*** creeping to suberect, reddish, 15–45 cm long, 1.0–3.0 mm diam., articulate, not flagelliform or stoloniferous, 3–4-branched. ***Rhizophores*** dorsal, borne on the proximal half of the stems, stout, 0.5–1.5 mm diam. ***Leaves*** heteromorphic throughout the stem and branches, chartaceous, with the upper surfaces dark green to yellowish-green and smooth, and the lower surfaces shiny yellowish-green and smooth. ***Lateral leaves*** along the main stems spreading, perpendicular to the stems, distant, narrowly oblong to oblong-lanceolate, 5.0–8.3 × 1.7–2.5 mm; bases truncate to rounded, the acroscopic bases slightly overlapping stems, the basiscopic bases with an auricle or lobe, 0.2–0.4 × 0.2–0.3 mm, free from the stems, glabrous; acroscopic margins greenish to slightly hyaline, continuously bordered by a band 1 or 2 cells wide of idioblasts, the idioblasts elongate, sinuate-walled, and papillate, the papillae in one row on each cell lumen, short-ciliate to denticulate in the basal third, otherwise dentate to denticulate distally, basiscopic margins similar to acroscopic margins, entire throughout, except short-ciliate along the distal 1/3; apices broadly acute to acute, tipped by 5–6 teeth; upper surfaces comprising irregularly shaped, somewhat rectangular to quadrangular, elongate, sinuate-walled cells, with stomata in 1–2 rows along the basiscopic margins and some scattered in the distal 1/3 of the acroscopic margins, the lower surfaces without idioblasts, comprising elongate, sinuate-walled cells, with stomata on 4–6 rows along the midribs and 6–7 rows submedial to submarginal on both halves of each leaf lamina. ***Median leaves*** peltate, non-overlapping on the main stems, imbricate on the branches, ascending, elliptic to ovate-elliptic, 2.5–3.8 × 1.2–1.7 mm; bases asymmetric and auricled, the auricles entire and divergent, the inner auricle small, broadly cuneate to rounded, 0.2–0.3 × 0.1–0.2 mm, and the outer auricle, well-developed, long rectangular, 0.6–1.0 × 0.2–0.4 mm; margins green and comprising elongate and sinuate-walled cells, the inner margin entire in the proximal 1/2, otherwise denticulate distally, the outer margins similar to the inner one; apices long-acuminate, each acumen 0.8–1.5 mm long, with denticulate margins, the upper surfaces puberulent with tooth-like hairs, and stomata in one row in the central portion, each acumen 0.8–1.5 mm long; upper surfaces with idioblasts and stomata in 1–3 rows along the midribs and submarginal, the idioblasts elongate and papillate, the papillae mostly in two rows on each cell lumen, but some idioblasts near the peltate base of the leaves to the stems with papillae in one row on each cell lumen and sinuate-walled, the lower surfaces without idioblasts and without stomata. ***Axillary leaves*** oblong to narrowly oblong along the main stem, 3.5–6.0 × 1.0–1.7 mm; bases biauriculate; margins greenish, bordered by a band 1 or 2 cells wide, the cells rectangular and laevigate; margins entire along proximal half, otherwise denticulate distally; apices acute to broadly acute, tipped by 6 or 7 teeth; both surfaces similar to the lateral leaves. ***Strobili*** terminal on branch and stem tips, globose proximally, compact, and quadrangular distally, 4.0–8.0 mm long. ***Sporophylls*** ovate and of three kinds, those that subtend the microsporangia and occupy almost the whole strobilus 0.8–1.4 × 0.5–0.6 mm, and those basally located (including the megasporophyll that subtends the single basal megasporangium and the sterile sporophylls protecting it) enlarged relative to others, 1.5–1.7 × 0.6–1.4 mm; all sporophylls lacking a laminar flap, with a well-developed keel along almost the entire laminae; bases rounded; margins denticulate, greenish; apices acute tipped by 2 or 3 tooth-like projections; ***dorsal sporophylls*** (including microsporophylls and sterile sporophylls protecting the megasporangium) with the upper and lower surfaces greenish to silvery green and with irregularly elongated, sinuate-walled cells, except for the half that overlap the ventral sporophylls, where the cells are silvery-hyaline; ***ventral sporophylls*** (including microsporophylls, megasporophyll, and sterile sporophylls protecting megasporangium) with epidermal cells on both surfaces similar in shape to those of dorsal sporophylls and silvery green to hyaline. ***Megasporangia*** one per strobilus, inserted adaxially on the basal portion of the ventral sporophyll; ***megaspores*** white, the proximal and distal faces with reticulate ornamentation, the reticulae closed, each reticulum wall foveolate, with the microstructure sponge-like, 560–600 μm diam. ***Microsporangia*** many per strobilus, inserted adaxially in the two dorsal rows of microsporophylls and throughout most of the two ventral rows of microsporophylls on the strobilus; ***microspores*** tan, the proximal and distal faces with echinate ornamentation, and with laevigate to slightly rugulate microstructure, 40–50 μm diam.

#### Habitat and distribution.

*Selaginella monoloba* grows as a terrestrial, creeping plant on granitic soils along roads and rivers in the departments of Cesar and Magdalena in Colombia, and in the state of Miranda in Venezuela.

#### Etymology.

The name derives from the Greek “*monos*,” meaning single, and “*lobos*,” meaning lobe, referring to the single short basiscopic auricle in the lateral leaves.

#### Conservation assessment.

*Selaginella monoloba* is known from ten gatherings representing widely distributed occurrences, eight from Colombia and two from Venezuela (including the type collection). The extent of occurrence (EOO = 37 km^2^) would suggest a Near Threatened (NT) conservation assessment. However, its area of occupancy (AOO = 40 km^2^) indicates that this species is Endangered (EN). This is the case when one considers the environmental problems faced by the Caribbean regions of Colombia and Venezuela, including deforestation, inefficient waste disposal, air and water pollution, and soil degradation (Rodríguez 2013). The only two collections of this species from Venezuela were collected within the Guatopo National Park in the state of Miranda, a protected area; however, the collections from Colombia are many more and represent widely separated locations. The others are found in areas with a high incidence of environmental problems, such as the departments of Magdalena and Cesar (Rodríguez 2013). Accordingly, *Selaginella monoloba* fulfills all requirements for an assessment of Endangered [EN B2ab(iii)] under the IUCN Standards and Petitions Committee (2022).

#### Additional specimens examined (paratypes).

**Colombia** • **Cesar**: Sierra Nevada de Santa Marta, southeastern slope, Hoya del Río Donachuí, ravine SE of Donachuí, alt. 1350–1500 m, 22 Sep. 1959, *J. Cuatrecasas & R. Romero C. 24341* (COL, F-image, US); • Mpio. Manaure, between Cordillera Central and Santa Marta, alt. 975 m, 24 Aug. 1946, *M.B. Foster & E. Smith 1585* (A); • Sierra de Perijá, Eastern of Manaure: Hacienda Nuevo Horizonte, El Podrido, alt. 1550–1600 m, 15 Nov. 1959, *J. Cuatrecasas & R. Romero C. 25385* (COL, US), • 16 Nov. 1959, *J. Cuatrecasas & R. Romero C. 25409* (COL). • **Magdalena**: Alto Río Frío Cabeceras del Río Congo, Ciudad Antigua, road to Alto Chimborazo, 10°59'N, 74°04'W, alt. 1100–1200 m, 26 Jul. 1989, *S. Madriñan & C.E. Barbosa 510* (F-image, MEXU, MO-image, VEN); • Santa Marta, Ridge above Onaca, alt. 3000 ft [914 m], 16 Aug. 1898, *H.H. Smith 2468* (F-image, GH, NCU-image, MO-image, NY-2 sheets, MICH-image, PH-image, TEX, US-image, WIS-image, WVA-image); • Mountains above Santa Marta, alt. 3000 ft [914 m], 27 Jul. 1946, *M.B. Foster et al. 1289* (COL); • Sierra Nevada de Santa Marta, Hacienda Cincinnati, alt. 1250–1500 m, 9 Aug. 1935, *C.W. Martin 3209* (US-image). **Venezuela** • **Miranda**: Parque Nacional de Guatopo: trail between carretera to Summit of Morro de Aramagual, passing Río Taquasito and Río San Lorenzo, alt. 500 m, 25 Nov. 1961, *J.A. Steyermark 90016* (NY, US).

#### Discussion.

*Selaginella monoloba* is characterized by its creeping to suberect habit with strongly articulated bistelic stems, leaf margins denticulate to short-ciliate, peltate median leaves with two divergent auricles: a short inner auricle overlapping the stem and a long outer auricle facing downward, lateral leaves oblong to narrowly oblong with short basiscopic auricles, and axillary leaves with two short, incurved auricles.

Initially, specimens of this species were identified as *Selaginella kunzeana* “Magdalena form” by Somers (1978) because of their resemblance to that species (e.g., basiscopic auricles on the lateral leaves, biauriculate median leaves, and similar spore size). Later, Smith (1985) cited the type collection (*Croat 21688*, UC) along with other specimens of this species as representing a potentially undescribed taxon, which is now formally described and validated here. *Selaginella monoloba* has peltate median leaves, a feature uncommon among articulated species within the genus. This character, along with short-ciliated margins bearing unicellular cilia and articulated bistelic stems, suggests that this species may belong to the *Selaginella horizontalis* (C. Presl) Spring complex. Members of this group share peltate median leaves, bistelic stems, and leaf margins that are serrate, denticulate, or long- to short-ciliate with unicellular cilia. Morphologically, *S. monoloba* is similar to *S. kunzeana* in its leaf shape and size, ascending to suberect habit, biauriculate median leaves, and lateral leaves with basiscopic auricles curved upward. However, it differs from *S. kunzeana* based on the diagnostic characters discussed.

### Selaginella mostaceroi

Taxon classificationPlantaeSelaginellalesSelaginellaceae

Valdespino & C.López sp. nov.

B0909F65-4B5B-5F50-A262-3AFDB329ED47

urn:lsid:ipni.org:names:77381378-1

Figs 32, 33, 34

#### Diagnosis.

*Selaginella mostaceroi* differs from *S. breynii* by its lateral leaves broadly (vs. narrowly) oblong, (1.6)1.7–2.2 (vs. 1.4–1.6) mm wide, with the acroscopic halves of the laminae almost twice as wide as the basiscopic halves of the laminae (vs. acroscopic and basiscopic halves of the laminae equal or nearly equal in width), and broadly obtuse or rounded (vs. broadly acute) apices.

**Figure 32.**
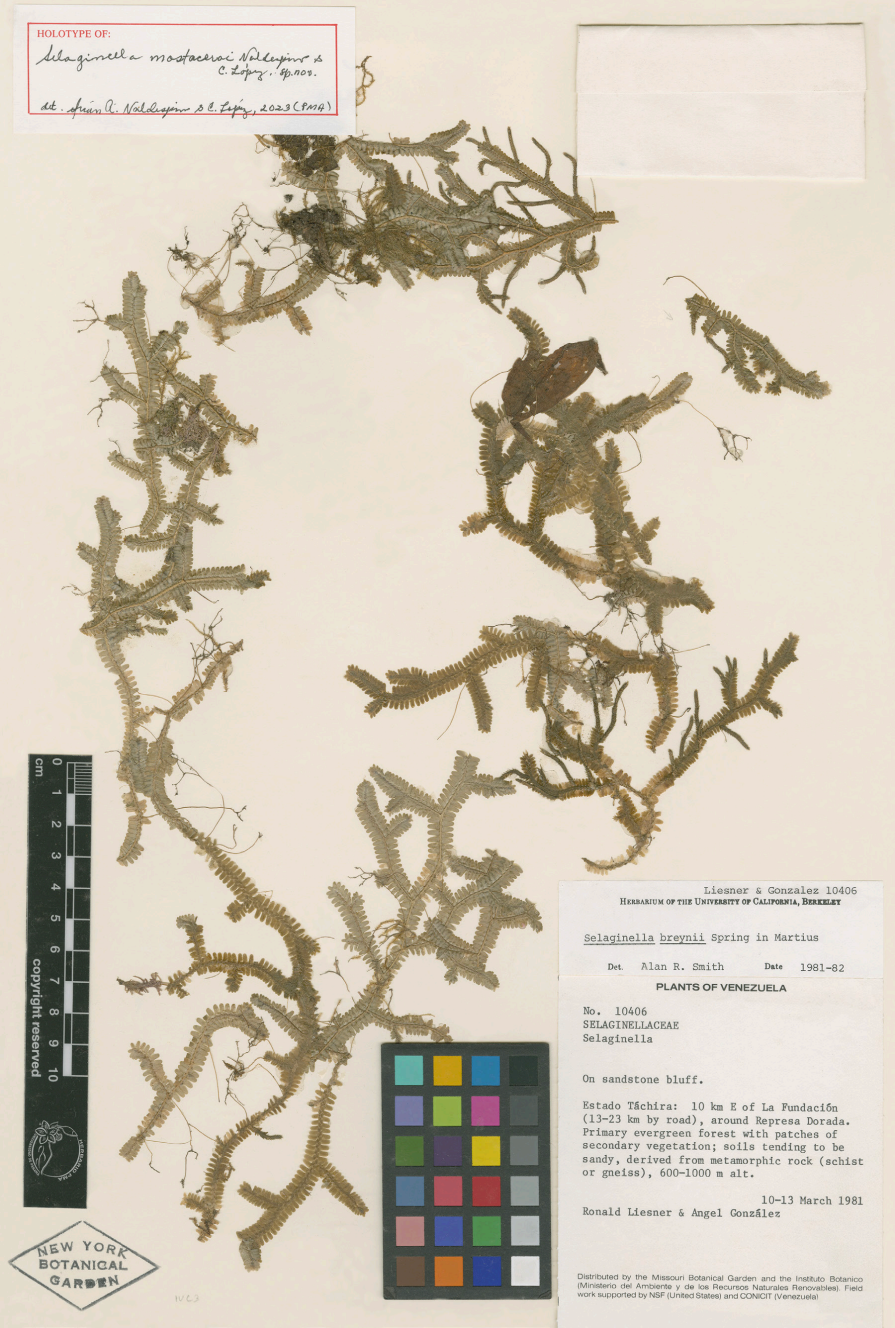
Holotype of *Selaginella mostaceroi* Valdespino & C.López. Digitized image courtesy of the herbarium of the University of Panama, Panama (PMA), from a loaned specimen from the herbarium of the New York Botanical Garden, *R.L. Liesner & A. González 10406*, NY.

**Figure 33.**
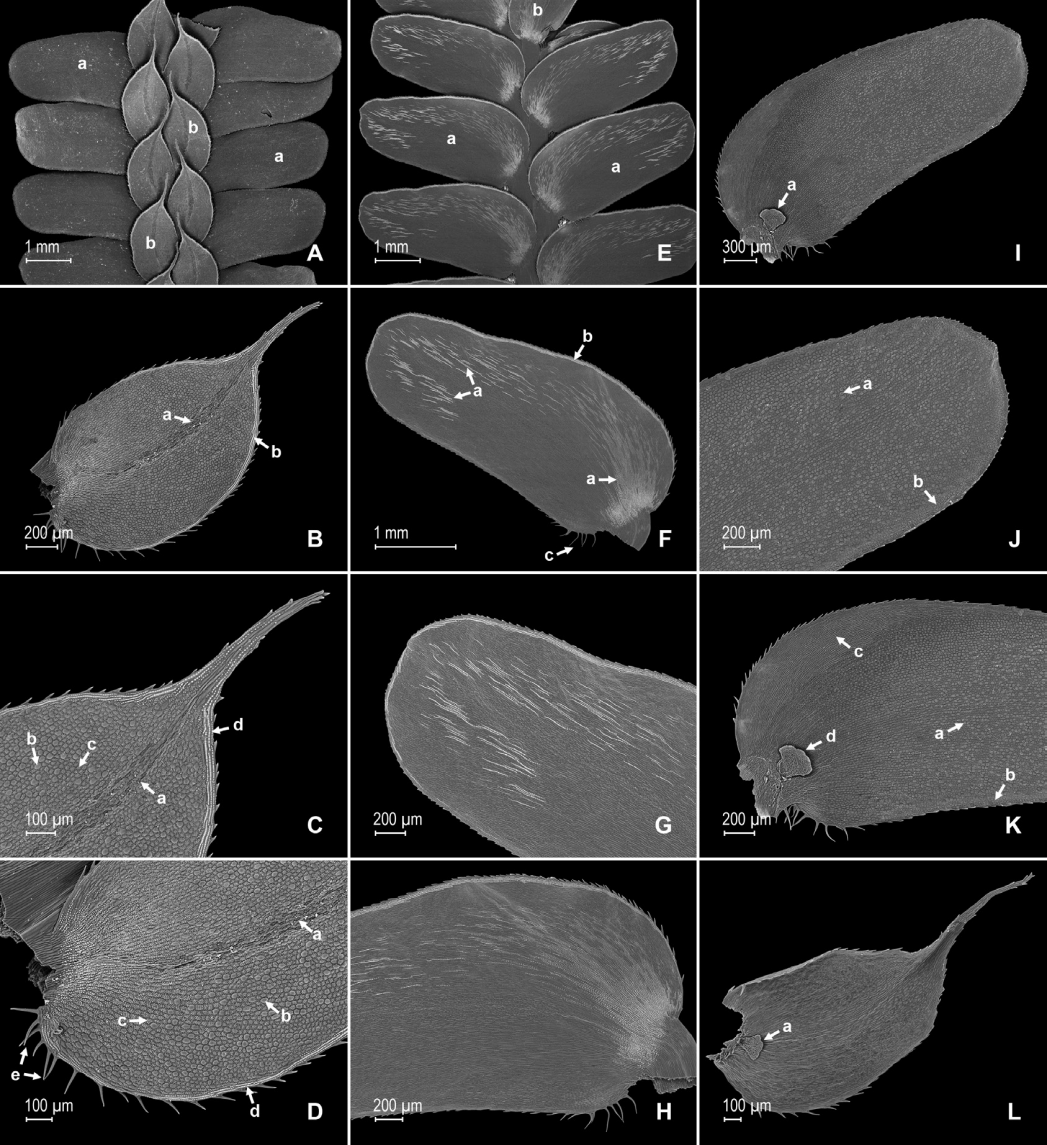
*Selaginella mostaceroi* Valdespino & C.López. **A**. Section of the stem, upper surface; note lateral (a) and median (b) leaves; **B**. Median leaf, upper surface; note stomata along the midrib (a) and marginal, elongate, straight-walled, and papillate idioblasts (b); **C**. Close-up of distal portion and apex of the median leaf, upper surface (same leaf as in **B**); note stomata along the midrib (a), roundish, rectangular to quadrangular, sinuate-walled, and laevigate (b) and papillate (c) cells, and marginal, elongate, straight-walled, and papillate idioblasts (d); **D**. Close-up of proximal portion and base of the median leaf, upper surface (same leaf as in **B**); note stomata along the midrib (a), roundish, rectangular to quadrangular, sinuate-walled, and laevigate (b) and papillate (c) cells, marginal, elongate, straight-walled, and papillate idioblasts (d), and long cilia near the outer base (e); **E**. Section of the stem, lower surface; note lateral (a) and axillary (b) leaves; **F**. Lateral leaf, lower surface; note elongate, straight-walled, and papillate idioblasts on the leaf lamina (a) and marginally (b), and long cilia along the proximal-most portion of the basiscopic base (c); **G**. Close-up of distal portion and apex of the lateral leaf, lower surface (same leaf as in **F**); note idioblasts as described in **F**; **H**. Close-up of proximal portion and base of the lateral leaf, lower surface (same leaf as in **F**); note idioblasts as described in **F**; **I**. Lateral leaf, upper surface; note the ligule (a); **J**. Close-up of the distal portion and apex of the lateral leaf, upper surface (same leaf as in **I**); note rounded, rectangular to quadrangular, laevigate cells (a) and rounded to quadrangular, papillate cells submarginally near the basiscopic margin (b); **K**. Close-up of the proximal portion and base of the lateral leaf, upper surface (same leaf as in **I**); note rounded, rectangular to quadrangular, laevigate cells (a) and rounded to quadrangular, papillate cells submarginally near the basiscopic margin (b); elongate, sinuate-walled, and laevigate cells (c), and fan-shaped ligule (d); **L**. Median leaf, lower surface; note fan-shaped ligule at the base (a). **A–L** from the holotype, *R.L. Liesner & A. González 10406*, NY.

**Figure 34.**
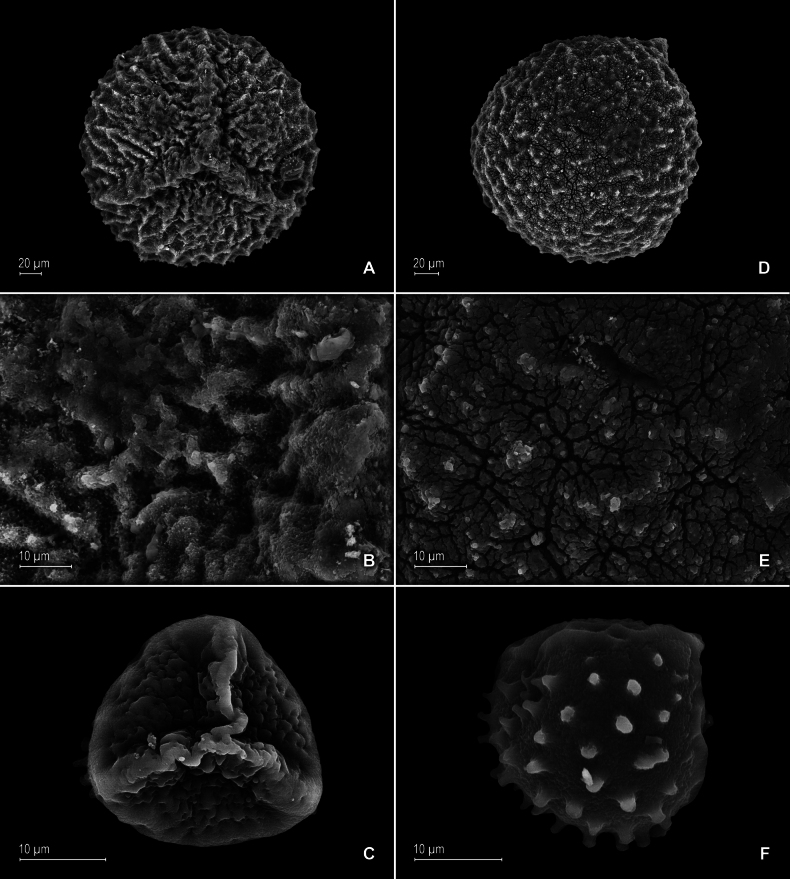
*Selaginella mostaceroi* Valdespino & C.López. **A**. Megaspore, proximal face; **B**. Close-up of the megaspore, proximal face (same spore as in **A**); **C**. Microspore, proximal face; **D**. Megaspore, distal face; **E**. Close-up of the megaspore, distal face (same spore as in **D**); **F**. Microspore, distal face. **A–F** from the holotype, *R.L. Liesner & A. González 10406*, NY.

#### Type.

**Venezuela** • **Tachira**: 10 km E of La Fundación (13–23 km by road), around Represa Dorada, [07°48'N, 71°40'W], alt. 600–1000 m, 10–13 Mar. 1981, *R.L. Liesner & A. González 10406* (holotype: NY!; isotypes: MO! [MO-1346878], P! [P01266019], PMA!, UC! [UC1480230]).

#### Description.

***Plants*** terrestrial. ***Stems*** creeping, stramineous, 8.5–15 cm long, 0.5–1.0 mm diam., non-articulate, not flagelliform or stoloniferous, 1- or 2-branched. ***Rhizophores*** axillary, borne throughout the stems, filiform, 0.1–0.3 mm diam. ***Leaves*** heteromorphic throughout the stems and branches, chartaceous, the upper and lower surfaces yellowish-green and smooth. ***Lateral leaves*** imbricate on main stems, perpendicular to the stems, broadly oblong to oblong, 3.0–4.5 × 1.6–2.2 mm; bases truncate to rounded, the acroscopic bases overlapping stems, the basiscopic bases free from the stems, long-ciliate; acroscopic margins hyaline to slightly hyaline, continuously bordered by a band 1–7 wide of idioblasts, the idioblasts elongate, sinuate-walled, and papillate, the papillae in one row on each cell lumen, short-ciliate to denticulate in the proximal 1/3, otherwise dentate to denticulate distally, basiscopic margins long-ciliate along the proximal 1/3, otherwise similar to the acroscopic margins; apices obtuse to broadly acute, tipped by 6–10 teeth; upper surfaces comprising irregularly shaped, somewhat rounded, rectangular to quadrangular, sinuate-walled cells, without stomata, the lower surfaces comprising elongate, sinuate-walled, and laevigate cells and elongate, sinuate-walled, and papillate idioblasts along the bases, the acroscopic half, and the distal 1/3 of the leaf lamina, with stomata in 3–5 rows along the midribs. ***Median leaves*** imbricate, ascending, broadly ovate to ovate-elliptic, 1.5–2.0 × 1.0–1.3 mm; bases oblique, glabrous, without auricles; margins hyaline, the inner margins continuously bordered by a band 2–3 cells wide of idioblasts, the idioblasts elongate, straight-walled, and papillate, the papillae in one row on each cell lumen, short-ciliate along proximal 1/2, otherwise dentate distally, the outer margins continuously bordered by a band 2–3 cells wide of idioblasts, the idioblasts elongate, straight-walled, and papillate, the papillae in one row on each cell lumen, long-ciliate along proximal 1/2, otherwise dentate distally; apices long-aristate, the arista 0.6–0.7 mm long, denticulate on margins and tipped by 2–4 teeth; upper surfaces comprising somewhat roundish, rectangular to quadrangular, sinuate-walled cells, some of these papillate, without idioblasts, with stomata in 2–4 rows along the midribs, the lower surfaces comprising elongate, sinuate-walled cells, without idioblasts. ***Axillary leaves*** broadly oblong to oblong, 2.0–3.8 × 1.0–1.5 mm; bases truncate; margins, apices, and surfaces similar to those of the lateral leaves. ***Strobili*** terminal and solitary on branch tips or borne subsessile on lateral branches along the main stems, quadrangular, 10–25 mm long. ***Sporophylls*** monomorphic, without a laminar flap, each with a dentate keel along the midrib, lanceolate to lance-ovate, 0.6–1.6 × 0.3–0.6 mm; bases rounded; margins hyaline; apices long-acuminate to aristate, the acumen or arista 0.2–0.4 mm long; ***dorsal sporophylls*** with the upper and lower surfaces as in the vegetative leaves, except for the half that imbricates with the ventral sporophylls which are greenish to silvery hyaline and with elongate cells; ***ventral sporophylls*** with both surfaces silvery green to hyaline. ***Megasporangia*** in two ventral rows; ***megaspores*** cream, the proximal faces rugulate-reticulate, the reticulae ill-defined to open, delimited by low ridges, without an equatorial flange, the microstructure rugulate, echinate, and perforate, the distal faces rugulate-reticulate to irregularly reticulate, reticulae open, delimited by low ridges, the microstructure verrucate and perforate, 185–225 µm diam. ***Microsporangia*** in two dorsal rows; ***microspores*** light orange, the proximal faces rugulate, the microstructure rugulate to smooth, the distal faces broadly capitate or baculate with the microstructure of each caput and baculum laevigate, and the rest of the surface rugulate to laevigate, 25–28 µm diam.

#### Habitat and distribution.

*Selaginella mostaceroi* is known only from the type collection near La Fundación, Táchira state, Venezuela. It grows on sandy soil in premontane to montane forests at 600–1000 m.

#### Etymology.

This species is named after our colleague Julián Mostacero Giannangeli (1972–), who is the Curator of lycophytes and ferns at the Herbario Nacional de Venezuela (VEN) and authored the Selaginellaceae treatment for the “Nuevo Catálogo de la Flora de Venezuela” (Hokche et al. 2008).

#### Conservation assessment.

The type collection was made in the La Fundación area, part of the Eastern Cordillera of the Andes, which is characterized by sandstone outcrops. Its vegetation includes several endemic species, as well as taxa shared with the Guiana Shield and the Amazon Basin (Aymard and Campbell 2007; Aymard 2022). To date, no additional specimens of *S. mostaceroi* have been recorded from these regions. Furthermore, Aymard (2022) noted that habitats in the La Fundación area have undergone continuous deforestation and degradation over the last five decades. Based on a single georeferenced occurrence, the extent of occurrence (EOO) cannot be estimated, whereas the area of occupancy (AOO) was 4 km^2^. Accordingly, we assess *S. mostaceroi* as Critically Endangered (CR) under IUCN criteria B2ab(iii) (IUCN Standards and Petitions Committee 2022).

#### Discussion.

*Selaginella mostaceroi* is characterized by its long-creeping, centipede-like stems, lateral leaves on the main stems broadly oblong in shape, long-ciliate along the proximal-most portion of the basiscopic margins, with rounded apices, and the median leaves broadly ovate to ovate-elliptic, with hyaline margins, long-ciliate along the proximal portion of the outer margins, long-aristate apices, and strobili solitary on the branch tips or borne subsessile on the lateral branches along the main stem.

The type collection of *S. mostaceroi* was originally identified as *S. breynii*, but it differs from the latter based on the characters described in the diagnosis. It is also distinct from *S. breynii* because of its lateral leaves with the acroscopic margins sparsely short-ciliate along the proximal 1/8–1/4, and otherwise sparsely denticulate to entire distally (vs. long-ciliate along the proximal 1/4, and densely denticulate distally). *Selaginella mostaceroi* is additionally distinguished from *S. breynii* by its median leaves with the inner and outer halves nearly equal in width (vs. outer half 1/8 wider than the inner half), the outer margins along the proximal 1/4 (vs. 3/4) long-ciliate, with the upper surfaces of the long-aristate apices glabrous (vs. covered by teeth-like projections or hairs), and the axillary leaves on the main stems long-ciliate along the proximal 1/8 (vs. along the proximal 1/3–1/2) of the leaf laminae, and with obtuse to rounded (vs. acute) apices.

*Selaginella mostaceroi* is also similar to other *Selaginella* species with a centipediform habit, such as *S. calosticha* and *S. imbricans*. It differs from *S. calosticha* by its broadly ovate to ovate-elliptic (vs. elliptic) median leaves with the outer margins long-ciliate along the proximal 1/2, otherwise dentate distally and the inner margins short-ciliate along proximal 1/2, otherwise both margins dentate to denticulate (vs. the outer margins long-ciliate throughout and the inner margins ciliate along proximal 1/2), and lateral leaves with rounded (vs. acute) apices tipped by several (vs. 1–4) teeth. *Selaginella mostaceroi* is distinguished from *S. imbricans* by its median leaves’ outer bases glabrous (vs. tufted with 2–6 stiff short cilia) and lateral leaves with rounded (vs. acute) apices tipped by several (vs. 1–3) teeth.

### Selaginella plagiochiloides

Taxon classificationPlantaeSelaginellalesSelaginellaceae

Valdespino & C.López sp. nov.

5F17A5D7-DADF-5BFE-8F4E-5F6DB6868B48

urn:lsid:ipni.org:names:77381379-1

Figs 35, 36, 37, 38

#### Diagnosis.

*Selaginella plagiochiloides* differs from *S. schultesii* Alston ex Crabbe & Jermy by its median leaves dentate to denticulate (vs. long-ciliate), the acroscopic margins of the lateral leaves short-ciliate along the proximal 1/2, otherwise denticulate to entire distally (vs. long-ciliate along the proximal 1/2–2/3, otherwise denticulate or entire distally), the cilia 1/6 or less (vs. 1/3–1/2 or more) of the width of the acroscopic halves of the leaf laminae, and with prominent midribs on the upper and lower surfaces (vs. only visible on the lower surfaces) and raised (vs. plane) in side view on the lower surfaces.

**Figure 35.**
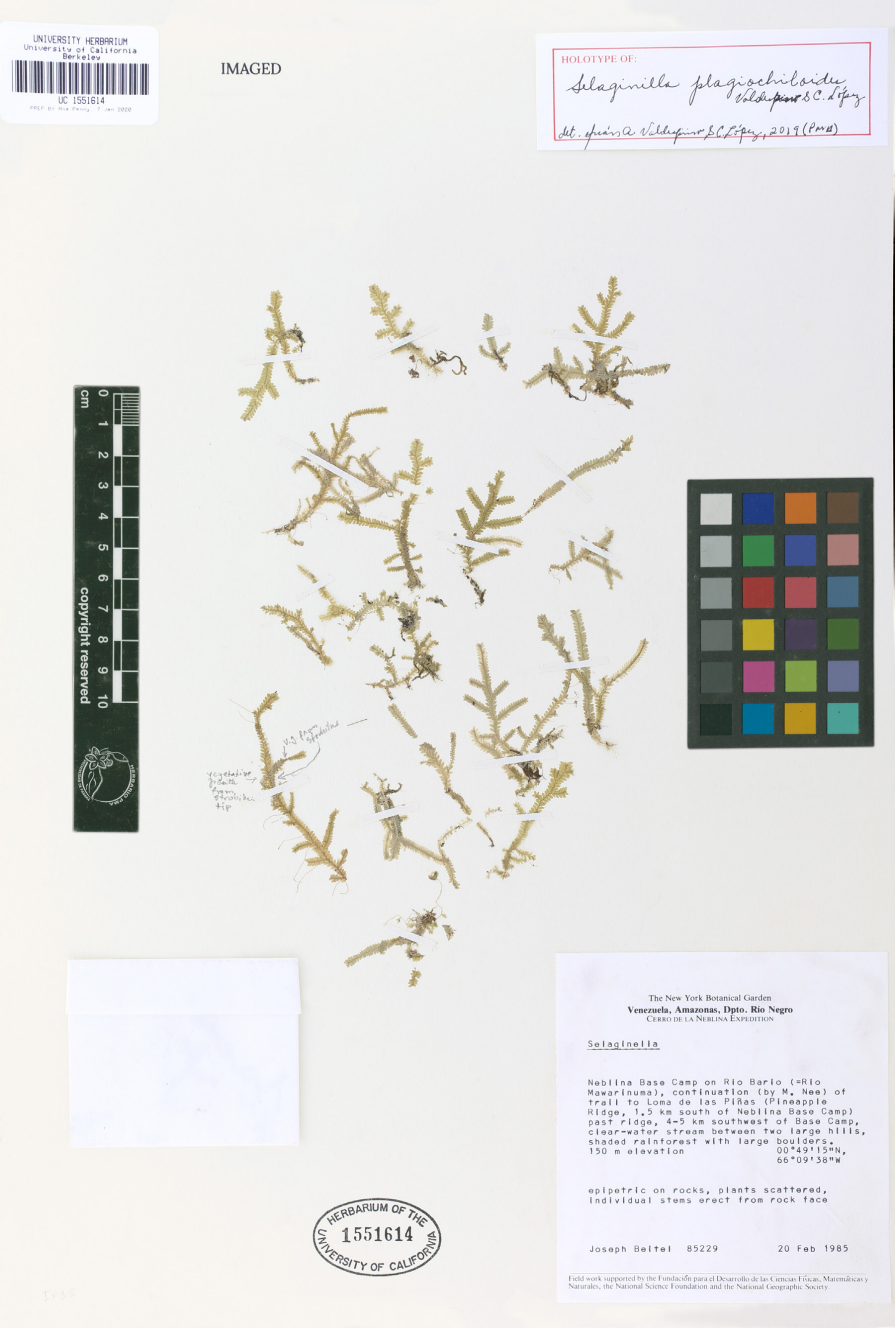
Holotype of *Selaginella plagiochiloides* Valdespino & C.López. Digitized image courtesy of the herbarium of the University of Panama (PMA), from a loaned specimen from the herbarium of the University of California, Berkeley, *J.M. Beitel 85229*, UC.

**Figure 36.**
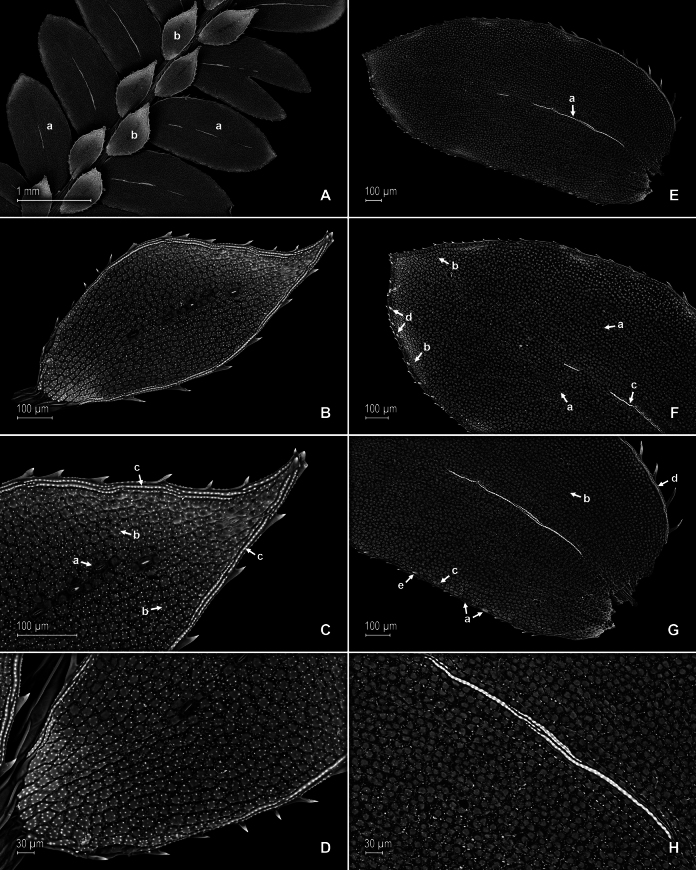
*Selaginella plagiochiloides* Valdespino & C.López. **A**. Section of the stem, upper surface; note lateral (a) and median (b) leaves; **B**. Median leaf, upper surface; **C**. Close-up of distal portion and apex of the median leaf, upper surface (same leaf as in **B**); note stomata along the midrib (a), rounded to quadrangular, sinuate-walled, and papillate cells on the leaf lamina (b), and marginal, elongate, straight-walled, and papillate idioblasts (c); **D**. Close-up of proximal portion and base of the median leaf, upper surface (same leaf as in **B**); note stomata, cells on leaf lamina, and margins as in **C**; **E**. Lateral leaf, upper surface; note elongate, straight-walled, and papillate idioblasts over the midrib (a); **F**. Close-up of distal portion of the lateral leaf, upper surface (same leaf as in **E**); note rounded to quadrangular, sinuate-walled, and laevigate (a) and papillate cells (b) on the leaf lamina, elongate, straight-walled, and papillate idioblasts on the leaf lamina (c), and submarginal tooth-like hairs (d); **G**. Close-up of the proximal portion and base of the lateral leaf, upper surface (same leaf as in **E**); note marginal stomata along basiscopic margin (a), rounded to quadrangular, sinuate-walled, and laevigate (b) and papillate (c) cells, elongate, straight-walled, and papillate idioblasts on the leaf acroscopic margin (d), and submarginal tooth-like hairs (e); **H**. Close-up of mid-section of the lateral leaf, upper surface (same leaf as in **E**); note leaf lamina cells as in **G**. **A–H** from the holotype, *J.M. Beitel 85229*, UC.

**Figure 37.**
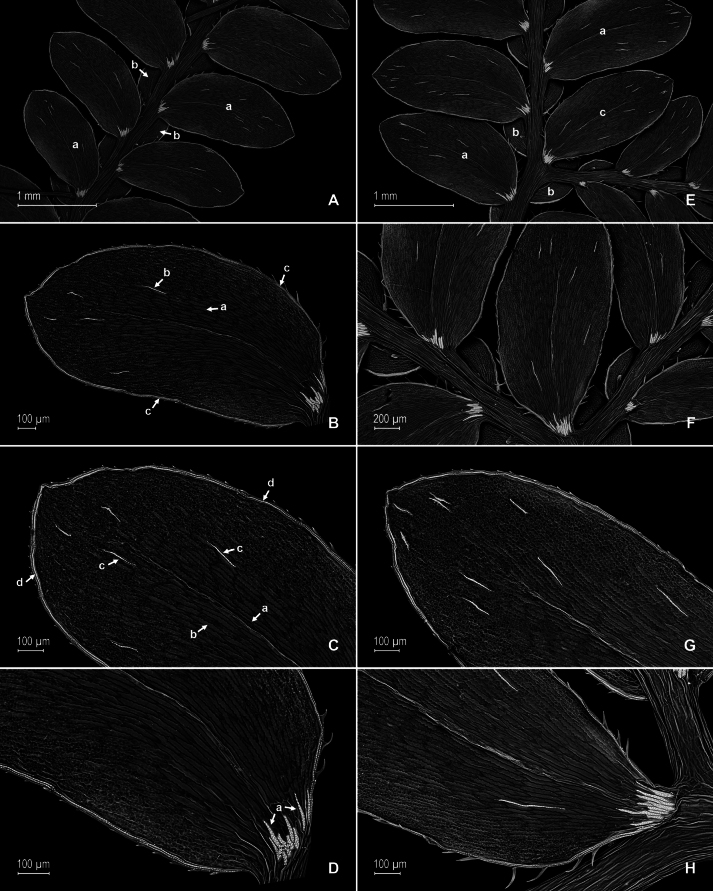
*Selaginella plagiochiloides* Valdespino & C.López. **A**. Section of the stem, lower surface; note lateral (a) and median (b) leaves; **B**. Lateral leaf, lower surface; note elongate, sinuate-walled, and laevigate cells (a) and elongate, straight-walled, and papillate idioblasts on the leaf lamina (b) and on the margins (c); **C**. Close-up of the distal portion and apex of the lateral leaf, lower surface (same leaf as in **B**); note stomata along the midrib (a), elongate, sinuate-walled, and laevigate cells (b), and elongate, straight-walled, and papillate idioblasts sparsely distributed along the distal portion of the leaf (c) and along the margins (d); **D**. Close-up of the proximal portion and base of the lateral leaf, lower surface (same leaf as in **B**); note stomata, cells on the leaf lamina and margins as in **C**, and elongate, straight-walled, and papillate idioblasts at the leaf base (a); **E**. Section of the stem, upper surface; note lateral (a), median (b), and axillary leaves (c); **F**. Close-up of section of the stem, lower surface (same section as in **E**); **G**. Close-up of the distal portion of the axillary leaf, lower surface; note cells on the leaf lamina and margins as in **C**; **H**. Close-up of proximal portion and base of the axillary leaf, lower surface (same leaf as in **G**); note cells on the leaf lamina and margins as in **C** and **D**. **A–H** from the holotype, *J.M. Beitel 85229*, UC.

**Figure 38.**
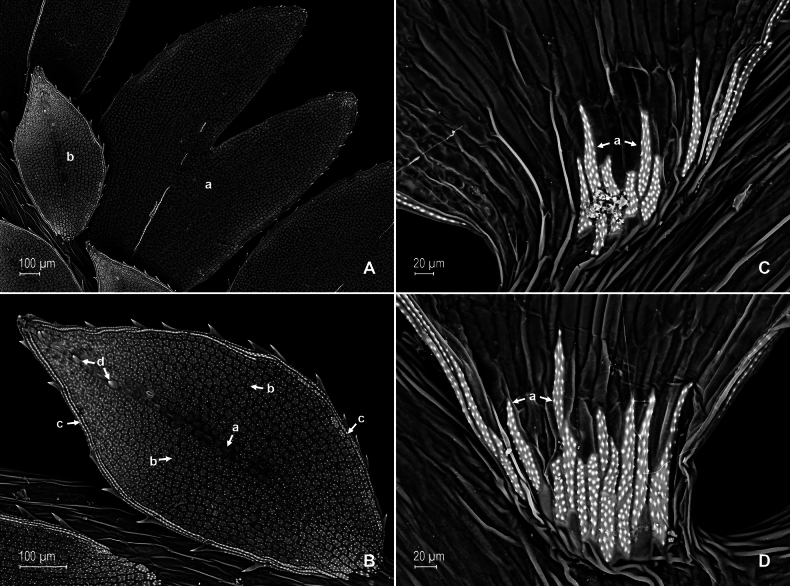
*Selaginella plagiochiloides* Valdespino & C.López. **A**. Close-up of section of the stem, upper surface; note lateral leaf with a bifurcated distal half (a) and median leaf (b); **B**. Median leaf, upper surface; note stomata along the midrib (a), rounded to quadrangular, sinuate-walled, and papillate cells on the leaf lamina (b), marginal, elongate, straight-walled, and papillate idioblasts (c), and teeth along distal 1/4 of the leaf lamina (d); **C**. Close-up of base of the lateral leaf; note elongate, straight-walled, and papillate idioblasts (a); **D**. Close-up of the base of the axillary leaf; note elongate, straight-walled, and papillate idioblasts (a). **A–D** from the holotype, *J.M. Beitel 85229*, UC.

#### Type.

**Venezuela** • **Amazonas**: Neblina Base Camp on Río Bario (= Río Mawarinuma), continuation of trail to Loma de las Piñas (Pineapple Ridge, 1.5 km S of Neblina Base Camp), past ridge, 4–5 km SW of Base Camp, 00°49'15"N, 66°09'38"W, alt. 150 m, 20 Feb. 1985, *J.M. Beitel 85229* (holotype: UC! [UC1551614], isotypes: PMA-image!, VEN! [Herb. No. 318917]).

#### Description.

***Plants*** epipetric. ***Stems*** prostrate, stramineous, 3–7 cm long, 0.1–0.4 mm diam., non-articulate, flagelliform, not stoloniferous, 1-branched. ***Rhizophores*** axillary-dorsal or axillary, borne throughout the stems, filiform, 0.02–0.1 mm diam. ***Leaves*** heteromorphic throughout, membranaceous, both surfaces glabrous, the upper surfaces green, the lower surfaces silvery green. ***Lateral leaves*** imbricate or slightly distant, ascending at 45° to the stems, ovate-oblong to oblong, 1.8–2.0 × 0.8–1.0 mm; bases rounded, the acroscopic bases slightly overlapping the stems, the basiscopic bases free from the stems; acroscopic margins on the upper surfaces narrowly hyaline along proximal 1/2, bordered by a band 1 or 2 cells wide of idioblasts, the idioblasts elongate, straight-walled, and papillate, the papillae in one row on each cell lumen, along the distal 1/2 bordered by a band 1 cell wide, the cells elongate and laevigate, on the lower surfaces hyaline, continuously bordered by a band 2 cells wide of idioblasts, the idioblasts elongate, straight-walled, and papillate, the papillae in one row on each cell lumen, short-ciliate along proximal 1/2, otherwise denticulate to entire distally, the basiscopic margins on the upper surfaces greenish, continuously bordered by a band 1 or 2 cells wide of rounded to quadrangular, sinuate-walled, and papillate cells, with 4–6 variously arranged papillae on each cell lumen, on the lower surfaces hyaline, continuously bordered by a band 1 or 2 cells wide of idioblasts, the idioblasts elongate, straight-walled, and papillate, the papillae in one row on each cell lumen, denticulate; apices acute, tipped by 1–3 teeth; upper surfaces composed of irregularly shaped, somewhat roundish to quadrangular, sinuate-walled, and laevigate cells, with some particularly along apical 1/4 and near the basiscopic margins papillate, each of these with the cell lumen bearing 2–9 irregularly arranged papillae, stomata submarginal and marginally along the basiscopic margins, with tooth-like hairs marginally to submarginally along the basiscopic margins, particularly near distal 1/4 of the leaf, the lower surfaces composed of elongate, sinuate-walled, and laevigate cells and of few scattered, elongate, sinuate-walled, and papillate idioblasts, each idioblast lumen with 25–65 papillae in 1 or 2 rows, stomata in 1 row along the midrib of the leaf lamina. ***Median leaves*** distant, ascending, lance-ovate to narrowly elliptic or elliptic-ovate, 0.6–1.0 × 0.3 to 0.4 mm; bases oblique, glabrous; margins hyaline, continuously bordered by a band 2 or 3 cells wide of idioblasts, the idioblasts elongate, straight-walled, and papillate, the papillae in one row on each cell lumen, dentate to denticulate throughout; apices long-attenuate to acuminate, each acumen 0.1–0.3 mm long, tipped by 2–4 teeth; upper surfaces comprising rounded to quadrangular, sinuate-walled, and papillate cells, each cell lumen covered by 3–6 papillae, with stomata in 1 row along central 1/2 or distal 3/4 of the midrib, the lower surfaces comprising elongate, sinuate-walled cells, with some idioblasts similar to those of the lower surface of the lateral leaves, without stomata. ***Axillary leaves*** similar to the lateral leaves in shape, bases, margins, and apices. ***Strobili*** borne along the main stem, loosely quadrangular, 0.7–4.0 mm, at tips often reverting to vegetative growth. ***Sporophylls*** monomorphic, without a laminar flap, each with a strongly developed and dentate keel along the midrib, lanceolate to broadly ovate, 1.0–1.3 × 0.6–0.8 mm; bases rounded; margins broadly hyaline, continuously bordered by a band 2–4 cells wide of idioblasts, the idioblasts similar to those on margins of the median leaves, dentate to denticulate; apices long-attenuate to aristate, each 0.2–0.3 mm long, with margins denticulate, and tipped by 1–3 teeth and with tooth-like projections on the upper surface; ***dorsal sporophylls*** with the upper surfaces green and cells as in the median leaves, except for the half that imbricates with the ventral sporophylls where the surface is hyaline, comprised of elongate, sinuate-walled, and papillate cells, the lower surfaces silvery green and comprising elongate, sinuate-walled cells; ***ventral sporophylls*** with both surfaces hyaline, comprising elongate, sinuate-walled, and papillate cells. ***Megasporangia*** in two ventral rows; ***megaspores*** not observed or measured. ***Microsporangia*** in two dorsal rows; ***microspores*** orange, surface ornamentation and diameter not observed or measured.

#### Habitat and distribution.

*Selaginella plagiochiloides* is known only from the type collection from Venezuela. It grows epipetrically on rocks near a water stream in the rainforest at 150 m.

#### Etymology.

The specific epithet refers to the overall stem and branch patterns of the new species, which resemble those of certain liverwort species within the genus *Plagiochila* (Dumort.) Dumort. (Plagiochilaceae–Marchantiophyta).

#### Conservation assessment.

*Selaginella plagiochiloides* is currently known from a single collection/locality in the Venezuelan Amazon (Cerro de la Neblina region). Based on the available georeferenced record, GeoCAT estimated an AOO of 4 km^2^, which falls within the threshold for Critically Endangered (CR) under criterion B2. Although the species occurs in a remote area, ongoing environmental pressures documented in the Venezuelan Amazon support the inference that habitat quality continues to decline (Huber 2001). Therefore, we preliminarily assess *S. plagiochiloides* as CR B2ab(iii).

#### Discussion.

*Selaginella plagiochiloides* is a small, creeping, centipediform species that resembles a leafy liverwort. It features the main stems ribbon-shaped and without branches, or once branched, ascending at 45° to the main stems, with stem tips often flagelliform.

We were unable to determine megaspore color, diameter, or ornamentation, nor microspore diameter and ornamentation, because megaspores were absent from most strobili examined, and microspores were rare. *Selaginella plagiochiloides* can easily be mistaken for a leafy liverwort because of its habit, stem shape, branch pattern, and small leaves, which resemble those of the genus *Plagiochila*, such as *P. adianthoides* (Sw.) Lindenb., *P. priceana* Gradst. & Benitez, *P. rutilans* Lindenb. from tropical America (Gradstein 2016; Gradstein and Benitez 2017), and *P. asplenioides* (L.) Dumort. from northwestern North America and western and central Europe to the Russian Far East (Stotler and Crandall-Stotler 2017). *Selaginella plagiochiloides* is further characterized by its lateral leaves with the acroscopic margins short-ciliate along the proximal 1/2 and otherwise denticulate distally, the basiscopic margins sparsely denticulate to entire, with the upper surfaces marginally to submarginally puberulent along the basiscopic margins, and median leaf margins narrowly hyaline and dentate to denticulate, and short strobili with their tips reverting to vegetative growth. Valdespino et al. (2015) reviewed cases of continuous vegetative growth from strobilus apices in *Selaginella* and used standardized codes to describe these shoot growth sequences (e.g., V/F, V/F/V, and V/F/V/F; where V = vegetative and F = fertile). In *S. plagiochiloides*, the V/F/V pattern appears to predominate, with fertile regions along the main stem interrupted by renewed vegetative growth, resulting in constricted strobili. Interestingly, this pattern is also observed in some species of *Plagiochila*, in which antheridia form along vegetative stems of antheridial gametophytes that later revert to a vegetative condition (e.g., *P. rutilans*; see fig. 3–I in Heinrichs et al. 2001).

*Selaginella plagiochiloides* somewhat resembles *S. schultesii* because of their small size, similar liverwort-like habit, and epipetric growth; however, several diagnostic traits distinguish the two species. Additionally, the stems of *S. plagiochiloides* are shorter, reaching 7 cm, while those of *S. schultesii* are longer, measuring 8–21 cm or more. It differs further from *S. schultesii* by its axillary leaves with the inner margins short-ciliate along the proximal 1/2, otherwise entire and the outer margins denticulate to entire throughout (vs. the inner and outer margins long-ciliate along the proximal 1/2–2/3, otherwise short-ciliate to denticulate distally), and the cilia on the inner margins measuring 1/6 or less (vs. 1/3–1/2 or more) of the width of the inner halves of the laminae.

*Selaginella plagiochiloides* is somewhat morphologically similar to other species in the “*Selaginella jungermannioides* (Gaudich.) Spring group”, especially *S. calosticha*, *S. imbricans*, *S. mawarinumensis*, and *S. mostaceroi*. It differs most notably from these species by its very small leaves, filiform rhizophores, median leaf apices that are long-attenuate to acuminate, and strobili with a V/F/V growth pattern.

Finally, in *Selaginella plagiochiloides* (Fig. 38A), occasional bifid leaves are observed, specifically among the lateral leaves, resembling the condition described by McAdam (2019) for *S. helvetica* (L.) Spring. This may be the first documented report of occasional bifid leaves in a Neotropical species of *Selaginella* and suggests that developmental lability in lycophylls may be more common in the genus than previously thought. Following McAdam’s (2019) interpretation, such variants might reflect latent genetic or developmental potential for leaf-form variation in lycophytes rather than a consistent taxonomic trait. Further research is needed to determine the frequency, anatomical and venation patterns, developmental origin, and possible environmental or genetic factors behind this condition in *S. plagiochiloides* and related species.

### Selaginella tricula

Taxon classificationPlantaeSelaginellalesSelaginellaceae

A.R.Sm. ex Valdespino & C.López sp. nov.

036A1610-8EC2-55B4-8DAF-1F9A3CE6EF8B

urn:lsid:ipni.org:names:77381380-1

Figs 39, 40, 41

#### Diagnosis.

*Selaginella tricula* is similar to *S. neospringiana* Valdespino in having median leaf margins sparsely ciliate with stiff, long cilia, but differs in its flagelliform (vs. determinate) stem apices, median leaves with long-acuminate to short-aristate (vs. long-aristate) apices, and the upper surfaces of lateral leaves with greenish (vs. straw-colored) midribs, with submarginal and marginal short, stiff hairs (vs. smooth) along the distal 1/2–3/4 of the basiscopic margins on the upper surfaces.

**Figure 39.**
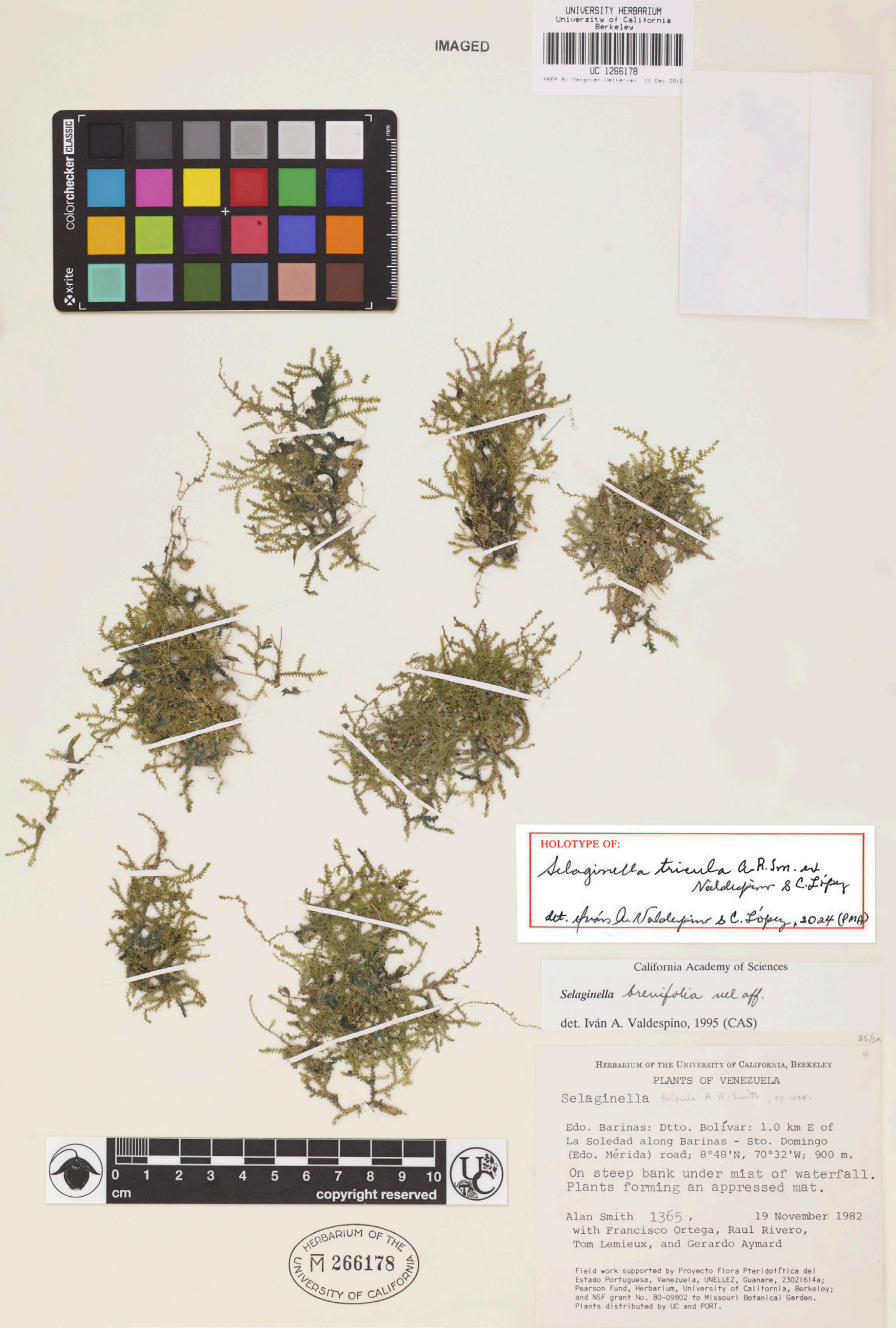
Holotype of *Selaginella tricula* A.R.Sm. ex Valdespino & C.López. Digitized image courtesy of the herbarium of the University of California, Berkeley, *A.R. Smith et al. 1365*, UC.

**Figure 40.**
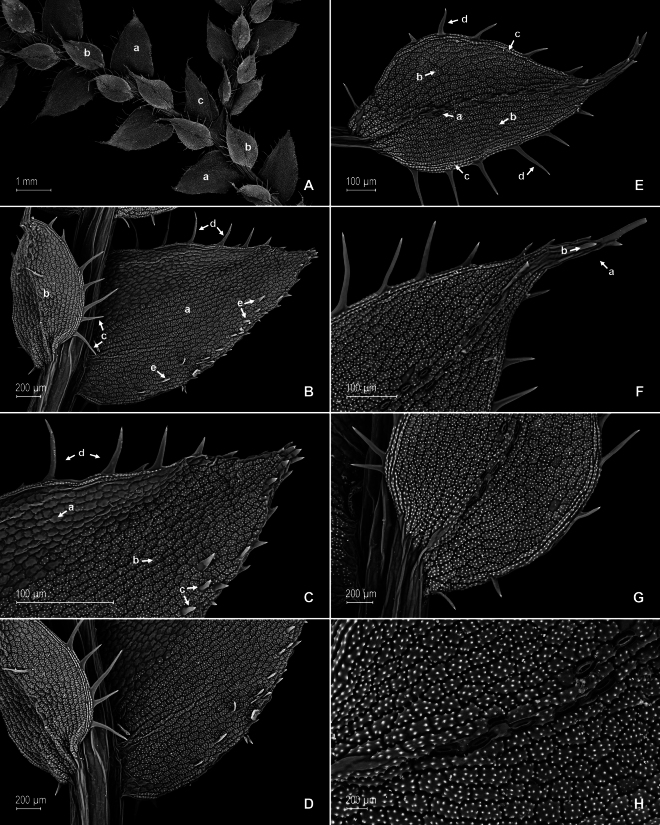
*Selaginella tricula* A.R.Sm. ex Valdespino & C.López. **A**. Section of the stem, upper surface; note lateral (a), median (b), and axillary (c) leaves; **B**. Close-up of a stem section, upper surface, showing lateral (a) and median (b) leaves; note long cilia on inner margin of the median leaf (c) and on acroscopic margin of the lateral leaf (d), and stiff hairs marginally and submarginally near the basiscopic margin of the lateral leaf (e); **C**. Close-up of distal portion and apex of the lateral leaf, upper surface (same leaf as in **B**); note rounded to quadrangular, sinuate-walled, laevigate (a) and papillate (b) cells, submarginal, tooth-like, short hairs along the basiscopic half (c), and long cilia along the distal portion of the acroscopic margin of the leaf lamina (d); **D**. Close-up of the proximal portion and base of the median and lateral leaves, upper surfaces (same leaves as in **B**); note leaf features as in **B**; **E**. Median leaf, upper surface, note stomata along the midrib (a), rounded to quadrangular, sinuate-walled, and papillate cells (b), elongate, straight-walled, and papillate idioblasts along the margins (c), and long marginal cilia (d); **F**. Close-up of the distal portion of the median leaf, upper surface (same leaf as in **E**); note stomata, cell features and margins of the lamina as described in **E**, long-acuminate to long-aristate apex (a), and short tooth-like hairs on the apex surface (b); **G**. Close-up of proximal portion and base of the median leaf, upper surface (same leaf as in **E**); note stomata, cell features of the lamina and the margins as described in **E** and **F**; **H**. Close-up of the median leaf, upper surface (same leaf as in **E**); note stomata and shape and features of the epidermal cells as described in **E–G**. **A–H** from the holotype, *A.R. Smith et al. 1365*, UC.

**Figure 41.**
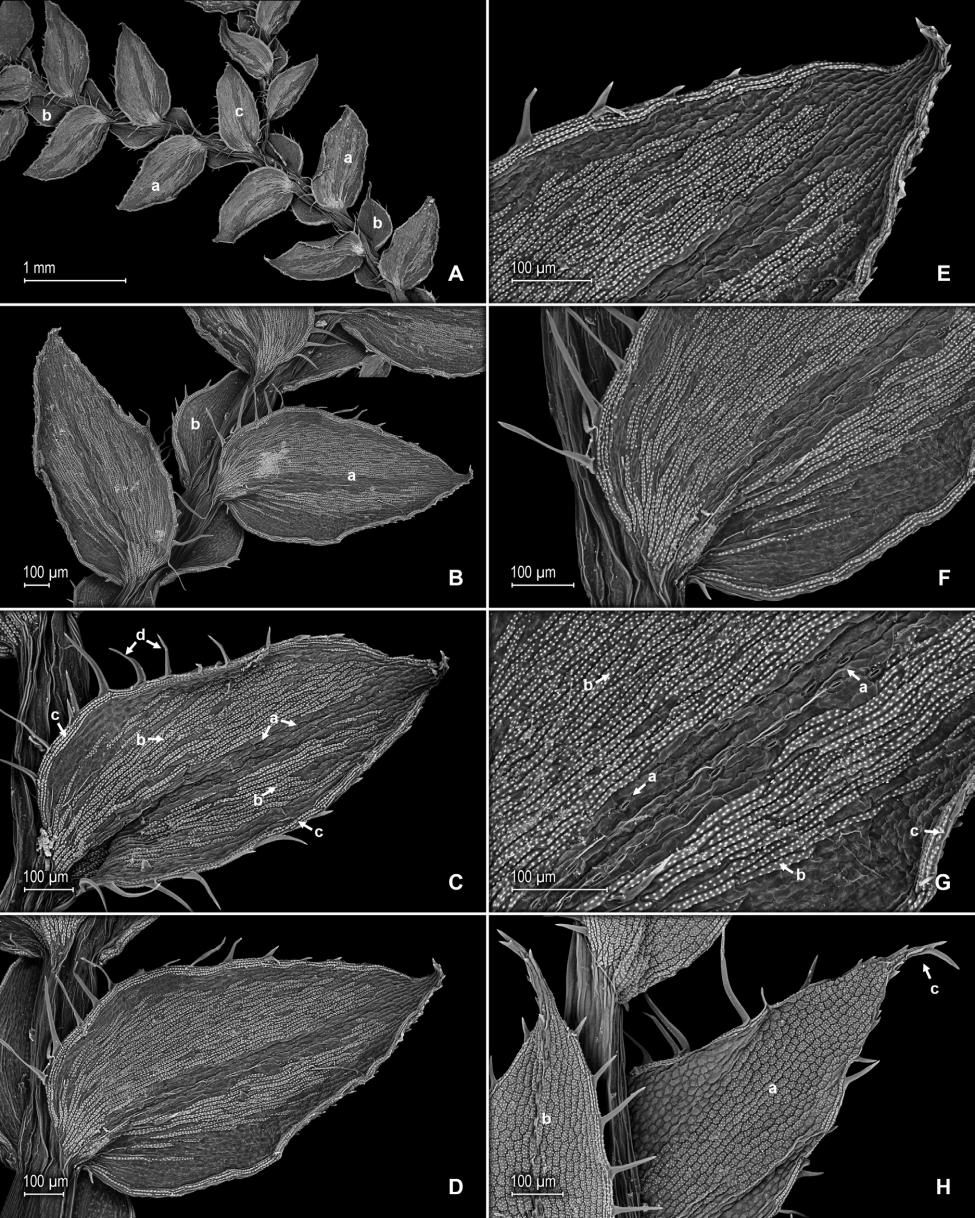
*Selaginella tricula* A.R.Sm. ex Valdespino & C.López. **A**. Section of the stem, lower surface; note lateral (a), median (b), and axillary (c) leaves; **B**. Close-up of a stem section, lower surface; note lateral (a) and median (b) leaves; **C**. Lateral leaf, lower surface; note stomata along the midrib (a), elongate, straight-walled, and papillate idioblasts on the leaf lamina (b) and on the margins (c), and marginal cilia (d); **D**. Lateral leaf, lower surface; note features described in **C**; **E**. Close-up of distal portion and apex of the lateral leaf, lower surface (same leaf as in **D**); note features as in **C**; **F**. Close-up of proximal portion and base of the lateral leaf, lower surface (same leaf as in **D**); note features described in **C**; **G**. Close-up of epidermal cell features of the lateral leaf, lower surface (same leaf as in **D**); note stomata along the midrib (a) and elongate, straight-walled, and papillate idioblasts on the leaf lamina (b) and basiscopic margin (c); **H**. Close-up of an axillary leaf (a) and portions of a median leaf (b); note acute apex tipped by divergent cilia (c). **A–H** from the holotype, *A.R. Smith et al. 1365*, UC.

#### Type.

**Venezuela** • **Barinas**: Dto. Bolívar, 1.0 km E of La Soledad along Barinas-Sto. Domingo (Edo. Mérida) road, alt. 900 m, 08°48'N, 70°32'W, 19 Nov. 1982, *A.R. Smith, F.J. Ortega, R. Rivero, T. Lemieux & G. Aymard 1365* (holotype: UC! [UC1266178]; isotype: MO! [MO-1841645]).

#### Description.

***Plants*** terrestrial or epipetric. ***Stems*** creeping, stramineous, 2.0–7.0 cm long, 1.0–2.0 mm diam., non-articulate, non-stoloniferous, 1- or 2-branched with stems and branches flagelliform. ***Rhizophores*** axillary, borne along the stems, filiform, 0.05–0.15 mm diam. ***Leaves*** heteromorphic throughout, membranaceous, both surfaces glabrous, the upper surfaces green, the lower surfaces silvery green. ***Lateral leaves*** spreading or slightly ascending, ovate to ovate-lanceolate, 0.9–1.5 × 0.6–0.8 mm; bases rounded to subcordate, the acroscopic bases overlapping the stems, the basiscopic bases free from the stems; acroscopic margins on the upper surfaces hyaline in a band 1–3 cells wide, with the cells elongate, straight-walled, and papillate parallel to the margins, the papillae in 1 or 2 rows over each cell lumen, the basiscopic margins greenish on the upper surfaces, composed of cells similar to those of the laminae on the upper surfaces, the margins on the lower surfaces conspicuously hyaline in a band 1–5 cells wide, with the cells elongate, straight-walled, and papillate parallel to the margins, the papillae in 1 or 2 rows over each cell lumen, the acroscopic margins long-ciliate along proximal 3/4, otherwise dentate distally, the basiscopic margins long-ciliate along proximal 1/8, otherwise entire and denticulate along distal 1/4; apices acute to attenuate, each 0.05–0.2 mm long, variously tipped by 1–4 teeth; upper surfaces comprising quadrangular or rounded, sinuate-walled cells, many of these covered by 6–20 papillae along the basiscopic 2/3 and the distal 1/4 of the acroscopic half of the leaf, without idioblasts, stomata scarce and sparsely distributed along the proximal 1/4 of the basiscopic margins, and with short, stiff hairs marginally and submarginally along the distal 1/2–3/4 of the margins, the lower surfaces comprising elongate, sinuate-walled, and laevigate cells, and a wide band of elongate, sinuate-walled, and papillate idioblasts on both sides of the midribs, the papillae in 1–3 rows on each cell lumen, with stomata in 1–3 rows along the midribs. ***Median leaves*** distant, ascending, elliptic or ovate-elliptic, 0.4–0.8 × 0.2–0.5 mm; bases oblique; margins on the upper and the lower surfaces hyaline in a band 2–4 cells wide, the cells elongate, straight-walled, and papillate, the papillae in 1–3 rows on each cell lumen, sparsely long-ciliate along the inner and outer margins or sparsely long-ciliate along the distal 1/2–3/4 of the outer margins; apices long-acuminate to short-aristate, each arista 0.1–0.3 mm long, marginally denticulate distally, tipped by 1–3 teeth; the upper and lower surfaces without idioblasts, the upper surfaces comprising quadrangular or rounded, sinuate-walled, and papillate cells, each cell lumen covered by 4–22 papillae, with stomata in one row along distal 3/4 of the midribs and few along proximal 1/4 of the outer margins, the lower surfaces comprising elongate, sinuate-walled, and laevigate cells, without stomata. ***Axillary leaves*** lanceolate or ovate, 0.8–1.7 × 0.3–0.7 mm; bases subcordate or rounded; margins hyaline, similar to those of the lateral leaves, the inner margins sparsely long-ciliate along proximal 2/3, otherwise denticulate distally, the outer margins long-ciliate along the proximal 1/8, otherwise entire to denticulate distally; apices similar to those of the lateral leaves. ***Strobili***, sporophylls, sporangia, and spores absent.

#### Habitat and distribution.

*Selaginella tricula* has only been recorded from its type collection in Barinas State, Venezuela. It inhabits steep banks beneath waterfalls, wet boulders, and shaded sandstone bluffs within submontane and montane forests at an altitude of 900 meters.

#### Etymology.

The specific epithet was first proposed by Smith (1985) and is derived from the Greek prefix “*thrix*,” Latinized as “*trich*, *tricho*,” meaning “hairy or hair-like,” and the diminutive suffix “-*ula*,” meaning “little or small.” It likely refers to the short, tooth-like hairs on the upper surface along the basiscopic margins of the lateral leaves, both submarginal and marginal. We believe the specific epithet accurately reflects a key feature of the newly proposed species and, therefore, has been retained.

#### Conservation assessment.

*Selaginella tricula* is currently known only from the type locality in Bolívar Municipality, Barinas State, Venezuela, where it was collected in humid, misty waterfall habitats. As in many *Selaginella* species, it is likely associated with moist microhabitats near watercourses and open wet areas. The type locality lies in the southern Venezuelan Andes, northeast of Sierra Nevada National Park and ca. 30 km west of Guaramacal National Park, both of which are recognized for high plant diversity and endemism. Despite the proximity of protected areas, deforestation associated with agricultural expansion and other environmental pressures (including pollution) has contributed to vegetation loss in Venezuela (Maldonado et al. 2011), potentially affecting the species’ suitable habitat. Additional evidence of habitat degradation in Barinas State includes vegetation decline in the Caparo Forest Reserve (Maldonado et al. 2011) and deforestation impacts on important watersheds (Peñaloza et al. 2008). Based on IUCN criteria (2022) with a single georeferenced occurrence, the extent of occurrence (EOO) cannot be estimated, whereas the area of occupancy (AOO) = 4 km^2^. Therefore, *S. tricula* is assessed as Critically Endangered [CR B2ab(iii)].

#### Discussion.

*Selaginella tricula* is characterized by its creeping, moss-like habit with flagelliform stems and branches, lateral leaves with the acroscopic margins long-ciliate along the proximal 3/4, with submarginal and marginal short, stiff hairs along distal 2/4 of basiscopic margins on the upper surfaces, and median leaves margins hyaline, bordered by a band of elongate, straight-walled, and papillate idioblasts, the inner margins long-ciliate, with stomata in one row along the distal 3/4 of the midribs and few along proximal 1/4 of the outer margins, and long-acuminate to short-aristate apices.

*Selaginella tricula* appears morphologically similar to *S. neospringiana*, which occurs in the eastern coastal Serra dos Órgãos in Rio de Janeiro, Brazil, whereas the former is found in the foothills of the Venezuelan Andes. They can be distinguished by the characters discussed in the diagnosis. *Selaginella tricula* is also set apart from *S. neospringiana* by its creeping (vs. ascending to erect) stems and rhizophores borne throughout (vs. along the proximal 1/4 of) the stems.

### Selaginella turingiana

Taxon classificationPlantaeSelaginellalesSelaginellaceae

Valdespino sp. nov.

BFD7B394-7471-506A-B550-21E027BEA28D

urn:lsid:ipni.org:names:77381381-1

**Figure 42.**
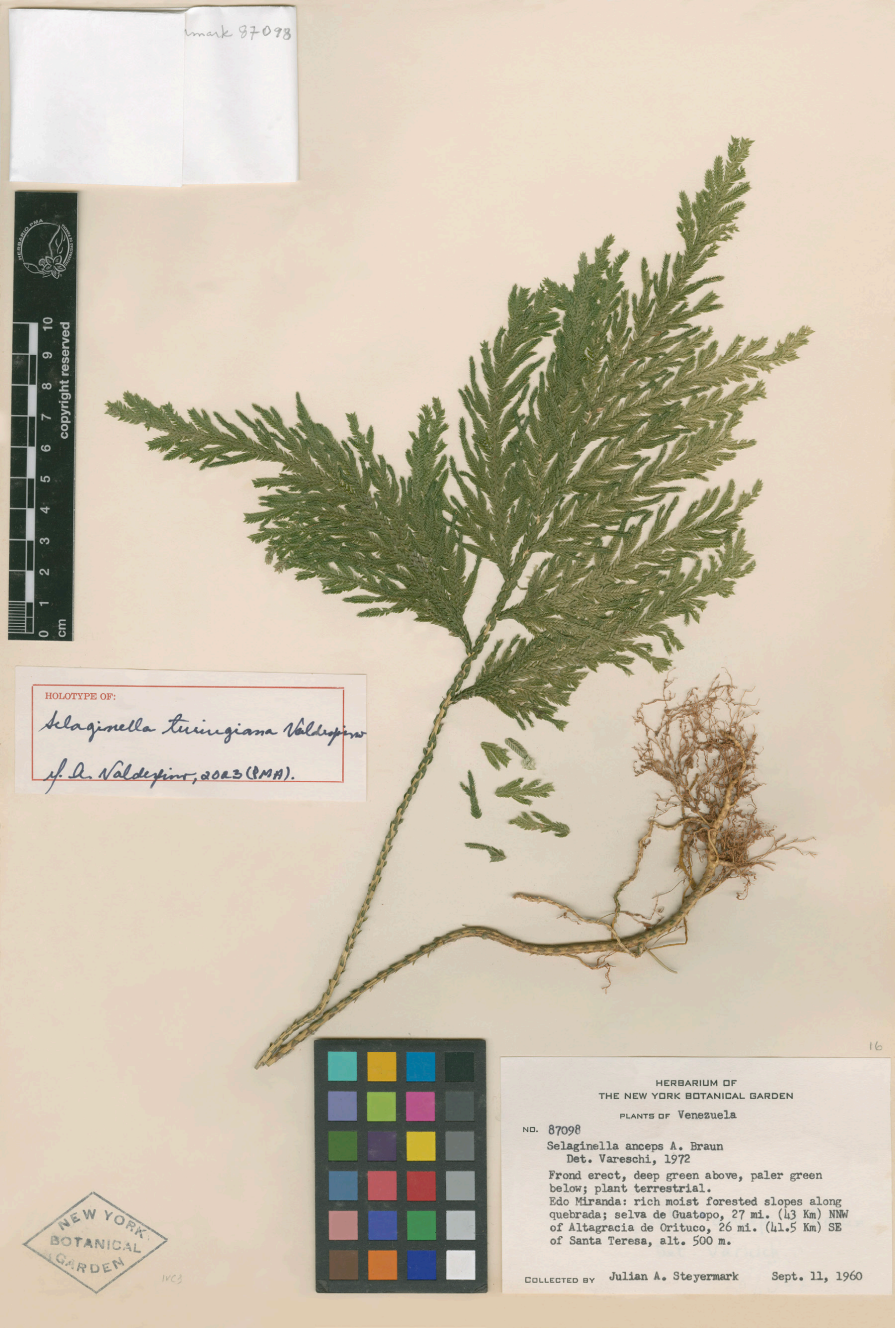
Holotype of *Selaginella turingiana* Valdespino. Digitized image courtesy of the herbarium of the University of Panama, Panama (PMA), from a loaned specimen from the herbarium of the New York Botanical Garden, *J.A. Steyermark 87098*, NY.

**Figure 43.**
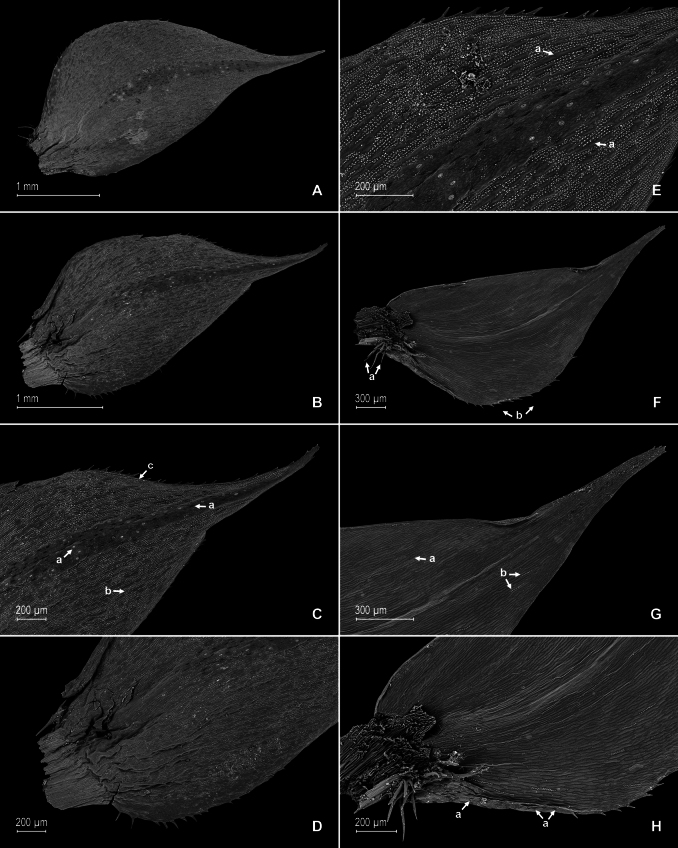
*Selaginella turingiana* Valdespino. **A**. Median leaf from the stem after the first branching, upper surface; **B**. Median leaf from the stem after the first branching, upper surface; **C**. Close-up of the middle and distal portions of the median leaf, upper surface (same leaf as in **B**); note stomata along the midrib (a), elongate, straight-walled, and papillate idioblasts throughout the leaf lamina (b) and the margins (c); **D**. Close-up of proximal portion and base of the median leaf, upper surface (same leaf as in **B**); note stomata and idioblasts on the leaf surface and margins of the lamina as in **C**; **E**. Close-up of the middle and distal portions of the median leaf, upper surface (same leaf as in **B**); note stomata, cell features on the leaf surface and margins of the lamina as in **C**, and sparsely intercalated, elongate, sinuate-walled, and laevigate cells (a); **F**. Median leaf, lower surface; note the outer base tufted with long hairs (a) and the outer margin dentate along the proximal 1/2–2/3 (b); **G**. Close-up of the distal portion and apex of the median leaf, lower surface (same leaf as in **F**); note elongate, sinuate-walled, and laevigate epidermal cells (a) and sparsely distributed, elongate, sinuate-walled, and papillate epidermal cells (b); **H**. Close-up of the proximal portion and base of the median leaf, lower surface (same leaf as in **F**); note the outer base as described in **F** and epidermal cells as described in **G**, and submarginal to marginal stomata along the proximal portion of the outer half of the leaf lamina near the outer margin (a). **A–H** from the holotype, *J.A. Steyermark 87098*, NY.

**Figure 44.**
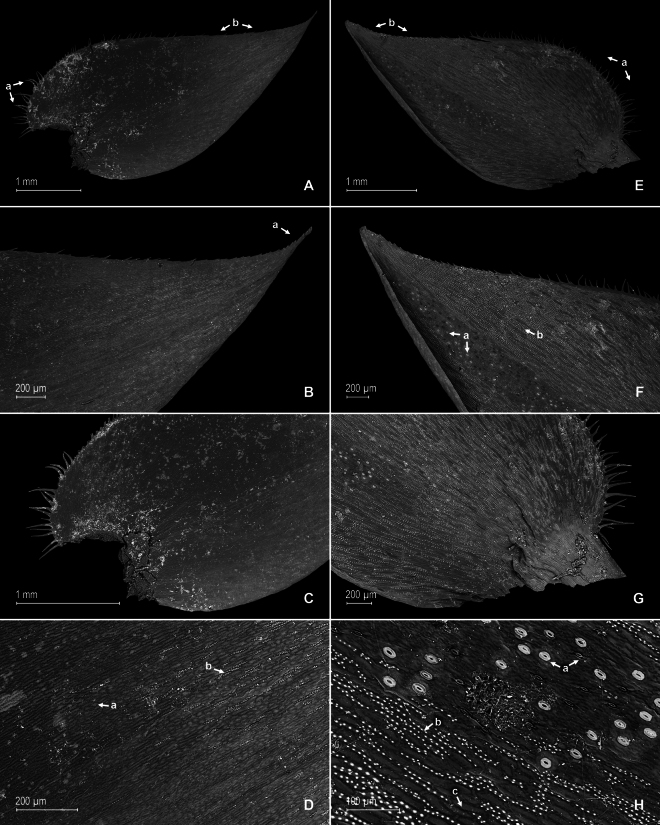
*Selaginella turingiana* Valdespino. **A**. Lateral leaf from the stem after the first branching; upper surface; note the acroscopic margin long- to short-ciliate along proximal 1/2–2/3 (a), otherwise dentate to denticulate distally (b); **B**. Close-up of the distal portion and apex of the lateral leaf, upper surface (same leaf as in **A**); note the acroscopic margin as in **A** and long-attenuate apex (a); **C**. Close-up of the proximal portion and base of the lateral leaf, upper surface (same leaf as in **A**); note the proximal, acroscopic margin as described in **A**; **D**. Close-up of the epidermis of the lateral leaf, upper surface; note narrowly quadrangular, sinuate-walled, and laevigate epidermal cells (a) and sparsely distributed, elongate, straight-walled, and papillate idioblasts (b); **E**. Lateral leaf from the stem after the first branching, lower surface; note the acroscopic margin long- to short-ciliate along proximal 1/2–2/3 (a), otherwise dentate to denticulate distally (b); **F**. Close-up of the distal portion and apex of the lateral leaf, lower surface (same leaf as in **E**); note stomata along the midrib (a), and mostly elongate, straight-walled, and papillate idioblasts (b), the acroscopic margin as in **E**, and long-attenuate apex as in **B**; **G**. Close-up of the proximal portion of the lateral leaf, lower surface (same leaf as in **E**); note the acroscopic and basiscopic margins as described in **E**, and stomata and epidermal cells as described in **F**; **H**. Close-up of the mid-section of the lateral leaf lamina, lower surface (same leaf as in **E**); note stomata along the midrib (a) and elongate, straight-walled, and papillate idioblasts (b), intermixed with elongate, slightly sinuate-walled, and laevigate cells (c). **A–H** from the holotype, *J.A. Steyermark 87098*, NY.

**Figure 45.**
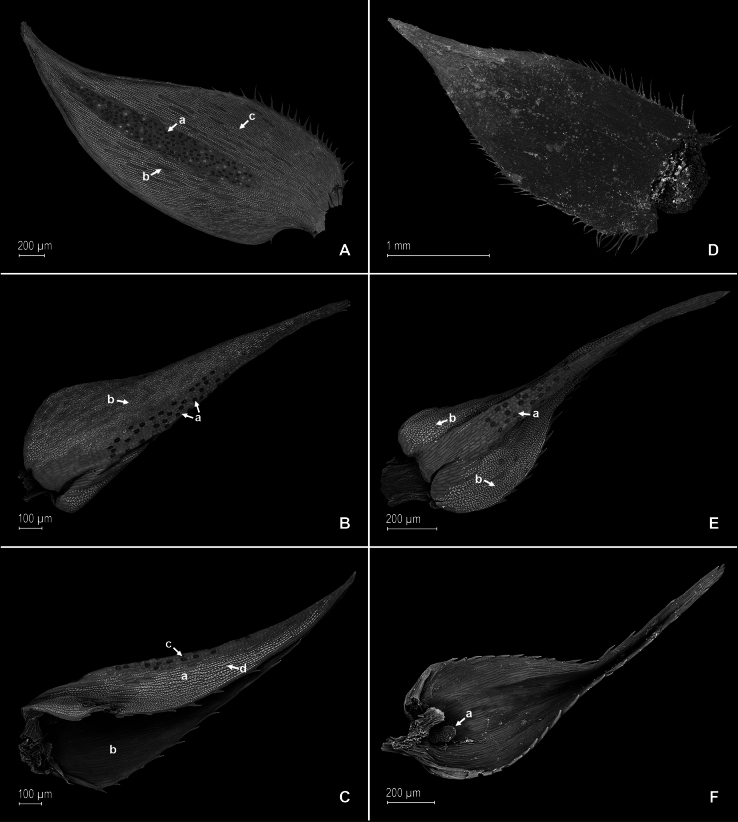
*Selaginella turingiana* Valdespino. **A**. Lateral leaf from the distal branches of the stem after the first branching, lower surface; note stomata along the midrib (a), elongate, slightly sinuate-walled, and laevigate cells (b), and elongate, straight-walled, and papillate idioblasts (c); **B**. Dorsal sporophylls, upper surface; note stomata along the midrib (a) and elongate, straight-walled, and papillate idioblasts (b); **C**. Dorsal sporophylls, half that imbricates with the ventral sporophylls, upper surface (a), and portion of the lower surface (b); note stomata along the midrib (c) and elongate, straight-walled, and papillate idioblasts on the upper surface (d); **D**. Axillary leaf, upper surface; **E**. Ventral sporophyll, upper surface; note stomata along the midrib (a) and elongate, straight-walled, and papillate idioblasts covering the sporophyll lamina (b); **F**. Ventral sporophyll, lower surface; note the ligule (a). **A–F** from the holotype, *J.A. Steyermark 87098*, NY.

**Figure 46.**
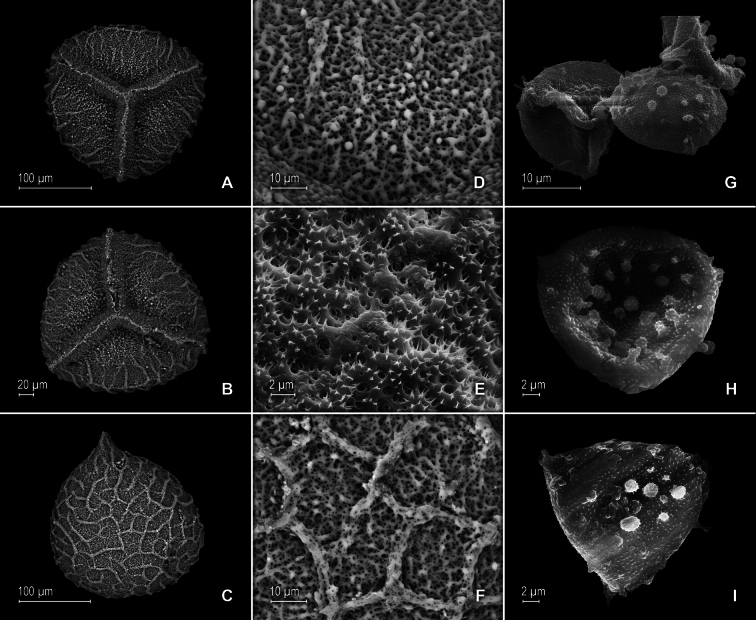
*Selaginella turingiana* Valdespino. **A**. Megaspore, proximal face; **B**. Megaspore, proximal face; **C**. Megaspore, distal face; **D**. Close-up of the megaspore, proximal face (same spore as in **A**); **E**. Close-up of the megaspore, proximal face (same spore as in **B**); **F**. Close-up of the megaspore, distal face (same spore as in **C**); **G**. Microspores, proximal and equatorial-distal faces; **H**. Microspore, distal face; **I**. Microspore, distal face. **A–I** from the holotype, *J.A. Steyermark 87098*, NY.

**Figure 47.**
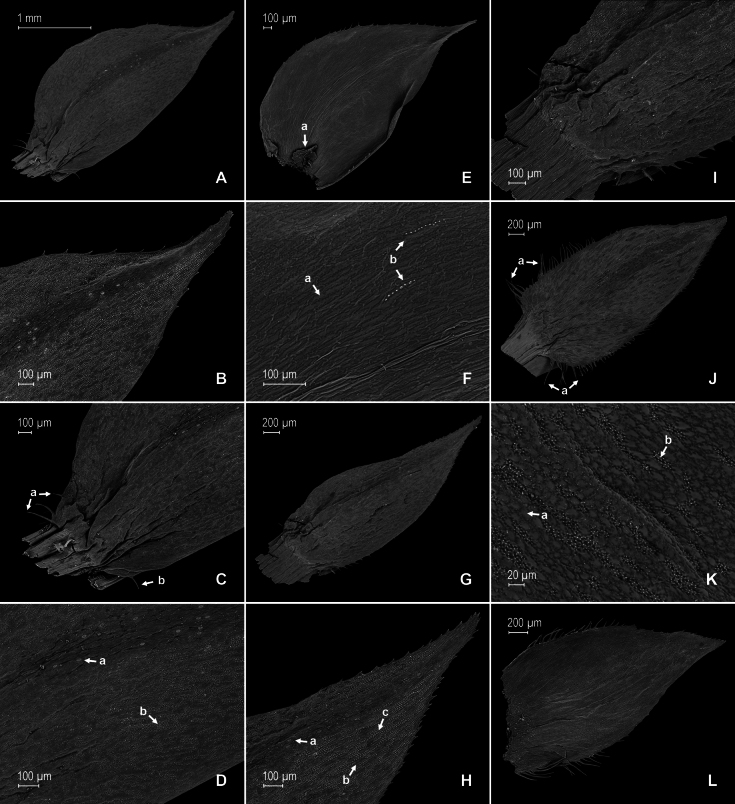
*Selaginella turingiana* Valdespino. **A**. Median leaf from the stem after the first branching, upper surface; **B**. Close-up of distal portion and apex of the median leaf, upper surface (same leaf as in **A**); **C**. Close-up of proximal portion and base of the median leaf, upper surface (same leaf as in **A**); note the outer base tufted with a few, long cilia (a) and proximal portion of the inner margin with a long cilium (b); **D**. Close-up of mid-section and inner half of the median leaf, upper surface (same leaf as in **A**); note stomata along the midrib (a), elongate, straight-walled, and papillate idioblasts (b); **E**. Median leaf from the stem after the first branching, lower surface; note the ligule (a); **F**. Close-up of mid-section of the median leaf from the stem after the first branching, lower surface (same leaf as in **E**); note elongate, sinuate-walled, and laevigate cells (a) and elongate, straight-walled, and papillate idioblasts (b); **G**. Median leaf from the distal portion of the stem after the first branching; upper surface; **H**. Close-up of distal portion and apex of the median leaf, upper surface (same leaf as in **G**); note stomata along the midrib (a), elongate, sinuate-walled, and laevigate cells (b), and elongate, straight-walled, and papillate cells (c); **I**. Close-up of proximal portion and base of the median leaf, upper surface (same leaf as in **G**); note cell features of the leaf lamina as described in **G**; **J**. Axillary leaf, lower surface; note stomata, cell features of the leaf lamina as described in **G**, and ciliate margins (a); **K**. Close-up of a lateral leaf, upper surface; note rectangular to quadrangular, sinuate-walled, and laevigate cells (a) and elongate, straight-walled, and papillate idioblasts (b); **L**. Leaf from the stem before branching, lower surface. **A–L** from a paratype, *H. Wagener 442*, B.

**Figure 48.**
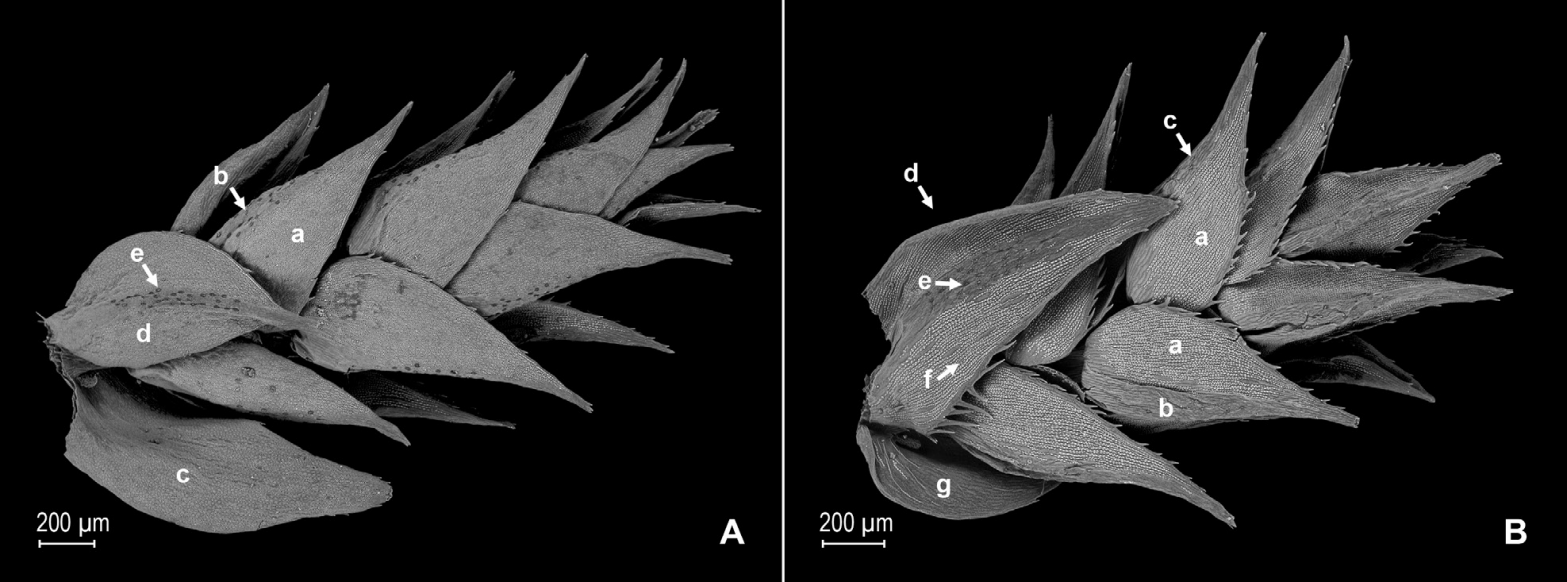
*Selaginella turingiana* Valdespino. **A**. Strobilus, upper view; note dorsal sporophyll (a), stomata along the midrib (b), lateral leaf (c), and median leaf (d) with stomata along the midrib (e); **B**. Strobilus, lower surface; note the inner half (a), outer half (b), and stomata along the midrib (c) of the ventral sporophylls, lateral leaf (d) with stomata along the midrib (e), elongate, straight-walled, and papillate idioblasts (f), and median leaf (g). **A** and **B** from a paratype, *H. Wagener 442*, B.

Figs 42, 43, 44, 45, 46, 47, 48

#### Diagnosis.

*Selaginella turingiana* differs from *S. altheae* Valdespino by its lateral leaves on the main stems, after fully heteromorphic, strongly ascending and parallel to the main stems, almost at 180° up to the ninth branches (vs. strongly ascending to slightly spreading at 45° after the first stem branches), with bases glabrous (vs. ciliate with 1–5 cilia, each 0.1 to 0.2 mm long), and the median leaves on main stems with the outer bases tufted with 4–12 short (vs. [4–]6–20 long) hairs, each 0.1–0.3 (vs. 0.4 or 0.5) mm long.

#### Type.

**Venezuela** • **Miranda**: Selva de Guatopo, 27 mi (43 km) NNW of Altagracia de Orituco, 26 mi (41.5 km) SE of Santa Teresa, [ca. 10°4'34.9"N, 66°24'59.9"W], alt. 500 m, 11 Sep. 1960, *J.A. Steyermark 87098* (holotype: NY!; isotypes: US! [Herb. No. 2419590], VEN! [Herb. No. 52176]).

#### Description.

***Plants*** terrestrial. ***Stems*** erect, stramineous, 24.0–51.0 cm in height, 1.0–3.0 mm diam. on main stems before first branches, non-articulate, not flagelliform, stoloniferous, 3- or 4-branched, the terminal portion of the stem similar in shape to the lateral branches (i.e., conform). ***Rhizophores*** axillary, ventral, dorsal, and lateral, borne on the lower-most part of the stems and throughout stolons, 0.3–1.0 mm diam. ***Leaves*** seemingly monomorphic and strongly appressed to the main stems shortly before the first branches (i.e., on proximal 10–26 cm of the stems or 1.0–4.0 cm below the first branches) and becoming fully heteromorphic (of three kinds of leaves: median, lateral, and axillary) on distal portion of the main stems (i.e., 1.0–4.0 cm below the first branches), coriaceous, those on the main stems before fully heteromorphic, triangular-lanceolate or narrowly deltate with prominently raised and truncate bases, the inner bases with a knob tufted with 2–6 short hairs, each 0.2 or 0.3 mm long, the outer bases rounded and glabrous; margins narrowly hyaline, the inner margins short-ciliate along proximal 1/2, otherwise denticulate distally, the outer margins entire throughout or entire along proximal 2/3 and denticulate along distal 1/3 or occasionally long-ciliate along proximal 1/3–1/2 and distally entire with each cilium 0.1–0.3 mm long; apices attenuate to long-attenuate, each (0.2) 0.4–0.8 mm long; upper surfaces dull to shiny light-green, striate or striate-corrugate, the lower surfaces shiny green to silvery green, striate with long idioblasts. ***Lateral leaves*** on the main stems distant, strongly ascending, parallel to the main stems, at ca. 180° to the stems up to the ninth branches and ovate-falcate in shape, whereas those along the distal portion of the stems becoming broadly-ovate, ovate-triangular to ovate-deltate, 2.5–3.7 × 1.0–2.0 mm; bases truncate to slightly rounded, glabrous, the acroscopic bases strongly overlapping stems, rounded, the basiscopic bases free from the stems, geniculate; acroscopic margins on the upper surfaces greenish and composed of rectangular cells or greenish along proximal 3/4, otherwise narrowly hyaline, bordered by a band of elongate idioblasts, the idioblasts papillate, the acroscopic margins on the lower surfaces bordered by greenish, rectangular to elongate, laevigate cells, short- to long-ciliate along proximal 1/2, otherwise dentate to denticulate along distal 1/2, the basiscopic margins greenish on both surfaces, entire throughout or denticulate along distal 1/8; apices gradually long-attenuate, each 0.1 or 0.2 mm long, entire at tips or often tipped by 2–5 short teeth; upper surfaces consisting of irregularly shaped, somewhat rectangular, sinuate- to straight-walled cells (often difficult to distinguish because of waxy deposits), with some of these on distal 1/2–3/4 of leaf lamina papillate, papillae usually in two rows or seldom in one row on each cell lumen, without stomata, the lower surfaces consisting of few elongate, sinuate-walled, and laevigate cells and of mostly elongate, straight-walled, and papillate idioblasts, papillae 8–15 in one or two rows on each cell lumen, with stomata in 2–7 rows along distal 2/3 of central-most portion of the midribs. ***Median leaves*** distant to slightly imbricate, ascending, broadly ovate to ovate-elliptic, ovate-triangular or ovate-deltate, 2.0–3.5 × 1.3–2.0 mm; bases truncate to truncate-oblique, without auricles, the inner bases rounded and glabrous, the outer bases truncate, often with a rounded knob tufted with 4–10 short or long hairs; the inner margins on the upper and the lower surfaces throughout their length, extended, greenish, continuously bordered by quadrangular cells, entire to denticulate along proximal 1/4, otherwise entire, the outer margins on the upper and the lower surfaces along proximal 1/4 incurved/folded inward, otherwise extended distally, continuously bordered by quadrangular cells, along distal 3/4 narrowly hyaline, continuously bordered by a band 1 or 2 cells wide of idioblasts, the idioblasts elongate, straight-walled and papillate, sparsely short- to long-ciliate along proximal 1/8, sparsely denticulate to short-ciliate along the central and distal 5/8, or entire along the distal 5/8; apices long-attenuate or long-aristate, each arista (0.3–) 0.5–1.0 mm long, tipped by 2–5 short teeth; upper surfaces corrugate, composed of elongate, straight-walled, and papillate idioblasts, the papillae 5–18, mostly in two rows on each cell lumen, intermixed with few, elongate, sinuate-walled, and laevigate cells, with stomata in 1–5 rows on the midribs along distal 2/3–3/4 and few submarginally, along basiscopic 1/4 of outer margins, the lower surfaces mostly comprising elongate, sinuate-walled, and laevigate cells and a few sparse, elongate, straight-walled, and papillate idioblasts distributed on the outer leaf half, with papillae in a single row, without stomata. ***Axillary leaves*** lanceolate, triangular or deltate, 2.0–3.1 × 0.9–1.6 mm; bases truncate or central portion truncate and with an inner, small knob or lobe, prominently raised, glabrous; margins long-ciliate along proximal 1/2, then short-ciliate for up to 1/4 of the proximal portion, otherwise denticulate on remaining distal 1/4; apices long-attenuate, each 0.3–0.5 mm long, tipped by 1–5 short teeth; the upper and lower surfaces as in the lateral leaves. ***Strobili*** terminal on main stem and branch tips, quadrangular, 0.3–1.4 cm long. ***Sporophylls*** monomorphic, without a laminar flap but with distal 1/2 strongly folded, each with a well-developed and glabrous keel along the midrib, ovate-lanceolate to lanceolate, 1.0–1.3 × 0.4–0.8 mm (the upper sporophylls slightly shorter and narrower); bases rounded; margins greenish, 1 or 2 cells wide, with the cells elongate, slightly sinuate-walled and laevigate, parallel to the margins, denticulate throughout on the dorsal sporophylls and short-ciliate along proximal 1/2 and dentate to denticulate on distal 1/2 on the ventral sporophylls; apices long-attenuate (the dorsal sporophylls slightly less attenuate than the ventral sporophylls by about 0.1 mm) to acuminate, each 0.3–0.7 mm long, tipped by 1 or 2 short teeth; ***dorsal sporophylls*** with the upper surfaces green, comprised of rectangular to elongate, sinuate-walled, and laevigate cells, except for the lamina half that imbricates with the ventral sporophylls, where it is silvery green, comprised of elongate, straight-walled, and papillate idioblasts, with stomata along the midribs, the lower surfaces silvery-green, composed of elongate, straight-walled, and laevigate cells; ***ventral sporophylls*** with the upper surfaces light or silvery-green, mostly composed of elongate, straight-walled, and papillate idioblasts with elongate, sinuate-walled, and laevigate cells along the midrib section and bases, with stomata along the midribs, the lower surfaces silvery-green, comprised of elongate, sinuate-walled, and laevigate cells, without stomata. ***Megasporangia*** in two ventral rows; ***megaspores*** white, the proximal faces rugulate to rugulate-reticulate, the reticulae ill-defined, open, and marginal, delimited by low ridges, with a slightly developed or absent equatorial flange, the microstructure laevigate or densely echinate, perforate to foveolate, the distal faces reticulate, each reticulum closed or open and delimited by low ridges, the microstructure laevigate, echinate, and perforate to foveolate, 180–230 µm diam. ***Microsporangia*** in two dorsal rows; ***microspores*** light orange, the proximal faces rugulate, the microstructure echinate, the distal faces capitate or baculate (by capita breaking off), with each caput or baculum, as well as the rest of the surface, having echinate microstructure, 17–22 µm diam.

#### Habitat and distribution.

*Selaginella turingiana* is a terrestrial species that grows in humid evergreen, premontane to montane tropical rainforests along the Coastal Mountain Range of Venezuela at elevations of 400–1219 m in the states of Aragua, La Guaira, Miranda, and Yaracuy.

#### Eponym.

This fern-like species is named after Alan M. Turing (1912–1954), a polymath genius who contributed significantly to various fields, including theoretical biology. He worked on developing a mathematical model linking plant phyllotaxis to Fibonacci numbers, a topic deserving more focus in *Selaginella* research. His work and inspiring statements, such as *“We can see only a short distance ahead, but we can see plenty there that needs to be done – Alan Turing”*, show he was far ahead of his time.

#### Conservation assessment.

*Selaginella turingiana* is known from multiple collections in the Cordillera de la Costa of northern Venezuela (including Miranda, La Guaira, and Yaracuy). GeoCAT estimates an extent of occurrence (EOO) of 9,085 km^2^ and an area of occupancy (AOO) of 40 km^2^. Although the AOO falls within the threshold for Endangered under criterion B2, the EOO corresponds to Vulnerable under criterion B1. Given the number of known records and localities and considering ongoing habitat degradation and environmental pressures documented for northern Venezuela and the Venezuelan Caribbean Basin, including effects associated with deforestation and pollution (Valjarević 2025), a continuing decline in habitat quality can be inferred. Accordingly, *S. turingiana* is here preliminarily assessed as Vulnerable (VU) under IUCN criterion B (IUCN Standards and Petitions Committee 2022).

#### Additional specimens examined (paratypes).

**Venezuela** • **Aragua**: Maracay [Maracay], *C. Vogl s.n*. (US). • **La Guaira-Miranda**: Cordillera de la Costa, NE de Guatire, Fila Juan Torres-Fila Las Perdices, Río Guayabal hacia el pueblo Guayabal, 10°31'N, 66°20'W, 19–22 Oct. 1993, *W. Meier 3385* (UC, VEN). • **La Guaira**: [parroquia Macuto, ca. 10°36'24"N, 66°53'33"W], *C.J.W. Schiede 493* (B), alt. 4000 ft [1219 m], Sep. 1849, *H. Wagener 442* (B-3 sheets). • **Miranda**: Parque Nacional Guatopo, S end of La Macanilla trail. 32 km (by air) NW of Altagracia de Orituco, 10°07'N, 66°31'W, alt. 600 m, 27 Aug. 1979, *M. Nee 17796* (NY, VEN), • E entrance near Santa Teresa [del Tuy], 12 Feb. 1973, [ca. 10°13'N, 66°38'W, 160 m], *T.B. Croat 21667* (MO), • Parque Nacional de Guatopo, Río Santa Cruz, between Santa Teresa and Altagracia de Orituco, 14.5 km from Los Alpes, 12 km from Ranchería Mi Querencia, alt. 520 m, 23 Nov. 1961, *J.A. Steyermark 89988* (GH, US, VEN); • carretera Sta. Teresa-Altagracia de Orituco, alt. 600–700 m, Jun. 1953, *L. Aristeguieta 1765* (US, VEN); • Mpio. Zamora, Macizo del Ávila, NE de Salmerón, between Río Chuspita and Río Salmerón, ruta of Mandarina, Agua Amarilla, 10°30'N, 66°24'W, alt. 800–1000 m, 2 Nov. 2008, *W. Meier & J. Jordan 15408* (UC-digital image, VEN). • **Yaracuy**: Cordillera de la Costa, límites del Dist. Nirgua-Dist. San Felipe, NE of Nirgua, Montaña El Zapatero, subida al cerro por el lado SE desde La Alegría, 10°14'N, 68°37'30"W, alt. 1000–1200 m, 24 Mar. 2005, *W. Meier et al. 11355* (NY, UC-digital image), • Río San Felipe, Valle del Río Yaracuy, Reserva Ecológica Privada Guáquira, on road to the mountain, S of the first river after station, 10°17'30"N, 68°38'30"W, alt. 500–700 m, 28 Aug. 2005, *W. Meier 11661* (UC-digital image); • Dist. San Felipe, Cordillera de la Costa, SE of San Felipe, Montaña El Zapatero, Río Yaracuy, Reserva Privada Guáquira, 10°17'30"N, 68°37'30"W, alt. 400–700 m, 21 Nov. 2004, *W. Meier 10585* (NY, UC-digital image, VEN); • Límite Dist. San Felipe-Dist. Nirgua, Cordillera de la Costa, NE of Nirgua, Montaña El Zapatero, La Alegría, hill separating the watershed of Río Terrón, 10°13'N, 68°37'W, alt. 950–990 m, 1 Jan. 2011, *W. Meier 1700* (UC-digital image). • **Without specific locality**: *N. Funck & L.J. Schlim s.n*. (B); 1845, *N. Funck & L.J. Schlim 57* (B); 1845–1846, *N. Funck & L.J. Schlim 57* (B, BM).

#### Discussion.

*Selaginella turingiana* is characterized by its frondose, fern-like, erect stems with stolons at the base, dorsal, ventral, and seemingly lateral rhizophores on the main stems, each 0.3–1.0 mm diam. The leaves along the main stems shortly before the first branches are seemingly monomorphic, ovate-triangular or ovate-deltate, throughout proximal 10–26 cm or 1.0–7.0 cm below the first branches with truncate bases, with the inner and the outer margins denticulate, except for the inner margins short-ciliate along the proximal 1/8, otherwise denticulate on both margins. Its lateral leaves on main stems strongly ascending, parallel to the main stems at almost 180° up to the ninth branches, ovate-falcate, and the acroscopic margins short- to slightly long-ciliate along the proximal 1/2 and the basiscopic margins entire throughout. It is also distinguished by its median leaves broadly ovate to ovate-elliptic, ovate-triangular or ovate-deltate with truncate bases, with the outer bases distinctly tufted with 4–10 short or long hairs, each 0.1–0.3 mm long, the outer margins narrowly hyaline along distal 3/4, and the apices long-acuminate or long-aristate, each (0.3–) 0.5–1.0 mm long. It is further characterized by its sporophylls with hyaline margins that are entire to denticulate margins. In addition, *S. turingiana* has the proximal faces of the megaspores rugulate to rugulate-reticulate with the microstructure laevigate or densely echinate, perforate to foveolate, while the distal faces are reticulate with each lumen closed or open and delimited by low ridges, and the microstructure of ridges and lumen laevigate, echinate, and perforate to foveolate. It is further characterized by its microspore proximal faces rugulate with the microstructure echinate, and the distal faces capitate or baculate (by capita breaking off), with each caput or baculum, as well as the rest of the surface, having echinate microstructure.

*Selaginella turingiana* belongs to the morphologically defined “*S. flabellata* group” and, within it, is morphologically close to *S. altheae*, *S. anceps* (C. Presl) C. Presl, *S. flabellata*, and *S. lechleri* Hieron. Most of the specimens mentioned here were previously identified as one of the species within the “*S. flabellata* group” mentioned above. *Selaginella turingiana* is similar to *S. altheae* in having geniculate basiscopic lateral leaf bases; however, the diagnostic characters set them apart. Additionally, its median leaf margins are distinctly hyaline (vs. greenish to narrowly hyaline), with the inner margins sparsely long-ciliate along the proximal 1/4, then becoming short-ciliate up to the proximal 1/2, and scarcely denticulate to entire along the distal 1/2 (vs. short-ciliate along proximal 2/3 and denticulate along distal 1/3), with long-acuminate or long-aristate (vs. acute to slightly acuminate) apices, each (0.3–) 0.5–1.0 (vs. ca. 0.1) mm long, and the sporophylls margins hyaline (vs. greenish), entire to denticulate throughout (vs. short-ciliate along the proximal 1/4–1/2 and denticulate distally). Furthermore, *S. turingiana* has megaspores with the proximal faces with smooth or occasionally densely echinate, perforate, and foveolate (vs. sparsely echinate, granulate, and perforate) microstructure, and distal faces closely reticulate with each lumen microstructure smooth, reticulate, perforate, and foveolate (vs. mostly open to somewhat closely reticulate with each lumen microstructure sparse and minutely echinate and perforate). Moreover, *S. turingiana* is found growing in premontane to montane forests at 400–1219 m in the Coastal Range, a northeastern extension of the Andes, in the states of Aragua, La Guaira, Miranda, Yaracuy, and the Federal District, Venezuela. In contrast, *S. altheae* is found in lowland and premontane forests at elevations of 140 to 450 m in the state of Amazonas, Venezuela. Accordingly, populations of *S. turingiana* are biogeographically separated from those of *S. altheae* by the vast tropical and subtropical grassland, savanna, and shrubland biome of Los Llanos in Venezuela.

*Selaginella turingiana* is also morphologically similar to the Lesser Antilles species, *S. flabellata* in having leaves on the main stems seemingly monomorphic below the first branches, leaf upper surfaces corrugate, leaves when fully heteromorphic along the main stem with lateral leaves ovate-falcate with the acroscopic margins short to slightly long-ciliate along proximal 1/2, and median leaves broadly ovate to ovate-elliptic with the outer bases tufted with long cilia. Specimens of *S. flabellata* that were reported from Venezuela at the varietal level as *S. flabellata* var. latifrons are here assigned to the newly elevated taxon at the species level, *S. anemosyra*.

*Selaginella turingiana* differs from typical *S. flabellata* by its leaves on the main stem becoming distinctly heteromorphic 6.0–7.0 (vs. 1.0–4.0) cm below the first branches of the stems, with the lateral leaves strongly ascending and parallel to the main stem in almost a 180° angle up to the ninth (vs. ascending to spreading in a 45° angle). Its median leaves below and above the first branches ovate-elliptic or elliptic (vs. lanceolate to ovate-lanceolate below the first branches and above it ovate to broadly ovate) with the outer bases oblique and prominent (vs. not prominent), those above the first branches with long-attenuate to long-aristate (vs. acute to short-attenuate) apices, each 1/4–1/3 (vs. less than 1/16) the length of the leaf lamina or 0.3 to 0.4 (vs. 0.1 or less) mm long. *Selaginella turingiana* differs further from *S. flabellata* by the oblique and prominent outer bases of its median leaves (vs. not prominent), each with an incipient (vs. lacking an) auricle, which is tufted with 2 or 3 (vs. 4–12) long cilia, each 0.1 or 0.2 (vs. 0.2–0.4) mm long. It is also distinct from *S. flabellata* by its lateral leaves ovate-lanceolate to ovate-triangular (vs. ovate to ovate-deltate), with the acroscopic margins short-ciliate along the proximal 3/4 (vs. 1/2) of the leaf laminae, and with long-attenuate (vs. acute) apices. Finally, it differs further from typical *S. flabellata* by its median leaf inner margins straight and entire (vs. straight to curved and short-ciliate along the proximal 1/4–1/3), with the outer half of the lamina with 5–7 (vs. 3) rows of submarginal and marginal stomata along the proximal 1/3, and rugulate-reticulate (vs. rugulate) megaspores with the proximal faces with (vs. without) a slightly developed equatorial flange.

*Selaginella turingiana* is easily distinguished from *S. anemosyra* by its median leaves below and above the first branches ovate to ovate-elliptic, or ovate-deltate (vs. ovate-orbicular), with the inner and outer halves almost equal in width (vs. outer halves 1/4 wider than inner halves), the outer bases truncate (vs. rounded), and with an incipient (vs. without a) knob tufted with 2 or 3 cilia, and long-attenuate to long-aristate (vs. apiculate) apices, 1/4–1/3 (vs. 1/8 or less) the length of the lamina. It is additionally distinct from the latter by its ovate-lanceolate to ovate-triangular (vs. oblong) lateral leaves with gradually long-attenuate (vs. falcate and acute) apices.

An incomplete specimen (i.e., *C. Vogl s.n*., US) lacks the upper branch segment on the main stem after the second branches and is tentatively assigned to *S. turingiana*. It displays dark green upper leaf surfaces and short, tapering median leaf apices, each measuring 0.1 or 0.2 mm long, features similar to those of *S. lechleri*. However, both the collection locality and other morphological traits are consistent with the geographic range and morphological characteristics of *S. turingiana*.

Finally, *Selaginella anceps* was reported from Venezuela (Smith 1985; Mostacero 2008); however, some specimens identified as such (e.g., *Bro. Gines 4199*, US!; *J.A. Steyermark 89988*, US!; *C. Vogl s.n*., US!) are here assigned to other taxa. For example, *J.A. Steyermark 89988* and *C. Vogl s.n*., both at US, correspond to *S. turingiana*, while *Bro. Gines 4199* at US is actually *S. viticulosa* Klotzsch. Likewise, other specimens identified as *S. anceps* from the Amazon region near the Neblina mountain, such as *Beitel & Buck 85064* (NY!, W-digital image!), *V.A. Funk & R.L. Liesner 6148* (NY!), and *V.A. Funk 6419* (NY!), were included in *S. altheae* by Valdespino (2017c). Accordingly, *S. anceps* is formally excluded from Venezuela; specimens previously identified as such from that country, specifically from the state of Amazonas, are now considered *S. altheae*, while those from Aragua, La Guaira-Miranda, Miranda, and Yaracuy are included here as *S. turingiana*. Currently, *S. anceps* is known only to occur in Costa Rica and Panama in Central America, and in Colombia, Ecuador, Peru, Bolivia, and Brazil in western South America.

##### New distribution record

### Selaginella mazaruniensis

Taxon classificationPlantaeSelaginellalesSelaginellaceae

Jenman

0EB5470A-DB65-54B5-8FB4-DFCC9AE9C7F3

Selaginella mazaruniensis Jenman, Gard. Chron. ser. 3, 22: 210. 1897 [as S. mazaruniense]. Type. Guyana, Demerara [Essequibo], Mazaruni River, Dec. 1896, *G.S. Jenman s.n*. (lectotype: designated by Góes-Neto et al. [2017: 2], NY! [NY00029566]; isolectotypes: B-image! [B 200123095-p.p., frag.], BM-image! [BM000936511, frag.], NY! [NY00029567]), figs 1–4 in Góes-Neto et al. (2017: 2–6).

#### Additional specimens examined.

**Venezuela** • **Amazonas**: **Amazonas**: Rio Negro: Cerro de la Neblina, along the river that flows out of Canyon Grande of Cerro de la Neblina, down river from base camp, across river from Neblina base camp on trail leading in a generally N-NE direction, 00°49'50"N, 66°05'45"W, alt. 140 m, 28 Feb. 1984, *V.A. Funk 6415* (MO, NY, VEN), *6430* (MO, NY, TEX).

#### Discussion.

*Selaginella mazaruniensis* was originally thought to be confined to the sandstone area of Guyana (Alston et al. 1981: 255); however, Góes-Neto et al. (2017: 2) reported it in the State of Amazonas, Brazil. It is here reported for the first time from Cerro de la Neblina, Amazonas, Venezuela.

*Selaginella mazaruniensis* grows as a terrestrial or epipetric plant on rocks in wet areas. It is characterized by its erect, fern-like habit with upright, stoloniferous stems reaching up to 30 cm in height, and the stem bases covered by red to reddish-brown, scale-like leaves that gradually become greenish-brown to green and seemingly monomorphic up to the proximal 1/2 of the stems, beyond which they become clearly heteromorphic. Immediately below the first branches, both median and lateral leaves are distinctly different morphologically (i.e., heteromorphic), with the upper surfaces corrugated. Additionally, it has ventral or ventro-axillary and dorsal rhizophores limited to the lowermost part of the stems. *Selaginella mazaruniensis* is also characterized by its median leaves with truncate to slightly oblique bases, with the inner leaf half twice as wide as the outer leaf half up to at least the first or second branches, and often up to the fourth branches, with narrowly hyaline and dentate or very short-ciliate margins, and with long-attenuate apices forming an acumen. It also has oblong lateral leaves, which are somewhat ascending or distinctly perpendicular to the stems after the second or third branches, with the bases truncate and attached to the stems, with the margins sparsely dentate or entire, with the acroscopic margins narrowly hyaline and the basiscopic margins greenish, and obtuse to broadly acute apices. Furthermore, in fully developed plants, the stems in dry collections are somewhat flattened, and the leafy portion of the plant tends to appear fan-like.

*Selaginella mazaruniensis* was originally reported as occurring in Venezuela by Vareschi (1969), who cited some specimens with the collector(s)’ name(s) (e.g., *Killip* and *Cardona*), but without a collection number. However, Smith (1985) excluded it from this country and suggested that specimens ascribed by Vareschi (1969) as *S. mazaruniensis* were actually *S. parkeri* (Hook. & Grev.) Spring. Mostacero (2008) followed Smith (1985) by excluding *S. mazaruniensis* from Venezuela and also followed Smith (1995), considering *S. mazaruniensis* restricted to Guyana. At the US herbarium, there are two collections by Killip from the same area cited by Vareschi in 1969 (i.e., *Killip 37400* & *Killip 37845*), which definitely correspond to an articulate species and are very likely *S. parkeri* (Hook. & Grev.) Spring. Accordingly, the specimens cited here are the first confirmed evidence that *S. mazaruniensis* occurs in Venezuela. However, duplicates or associated material of the same collections at US are identified as different species: *V.A. Funk 6415* as *S. palmiformis* and *V.A. Funk 6430* as *S. parkeri*. This discrepancy may reflect a specimen sorting error. Consequently, further study of these collections is needed to resolve discrepancies among herbarium determinations.

*Selaginella mazaruniensis* is morphologically similar to *S. amazonica* but can be distinguished by its lateral leaves on the main stems below the first branches being somewhat ascending to distinctly perpendicular (vs. distinctly ascending), oblong-ovate or oblong (vs. ovate-deltate to broadly ovate), and above the first branches mostly distant (vs. increasingly imbricate), with acroscopic margins hyaline (vs. greenish-hyaline), and entire or finely dentate to serrate (vs. serrate).

*Selaginella mazaruniensis* can also be mistaken for *S. palmiformis*, from which it is distinguished by its usually 2- or 3-pinnate (vs. 1-pinnate) branches and the lateral leaves with the acroscopic margins that are entire or finely dentate to serrate (vs. serrate to dentate at least along the proximal 1/2–2/3).

## Supplementary Material

XML Treatment for Selaginella anemosyra

XML Treatment for Selaginella cataniapensis

XML Treatment for Selaginella cultellifolia

XML Treatment for Selaginella guaramacalensis

XML Treatment for Selaginella liesneri

XML Treatment for Selaginella mawarinumensis

XML Treatment for Selaginella monoloba

XML Treatment for Selaginella mostaceroi

XML Treatment for Selaginella plagiochiloides

XML Treatment for Selaginella tricula

XML Treatment for Selaginella turingiana

XML Treatment for Selaginella mazaruniensis

## Additional information

### Artificial Intelligence (AI) use

The authors accept full responsibility for the content of the manuscript, including the disclosure of any use of AI.

No AI tools were used in the preparation of this manuscript.

### Author ORCIDs

I.A. Valdespino https://orcid.org/0000-0003-1004-8289

C.A. López https://orcid.org/0000-0003-1127-8721

J.I. Ceballos https://orcid.org/0000-0003-0556-9493

### Data availability

All of the data that support the findings of this study are available in the main text.
